# Dicyclopentaannelated Hexa‐*peri*‐hexabenzocoronenes with a Singlet Biradical Ground State

**DOI:** 10.1002/anie.202102932

**Published:** 2021-04-07

**Authors:** Qiang Chen, Martin Baumgarten, Manfred Wagner, Yunbin Hu, Ian Cheng‐Yi Hou, Akimitsu Narita, Klaus Müllen

**Affiliations:** ^1^ Synthetic Chemistry Max Planck Institute for Polymer Research Ackermannweg 10 55128 Mainz Germany; ^2^ Institute of Physical Chemistry Johannes Gutenberg-University Duesbergweg 10–14 55128 Mainz Germany; ^3^ Organic and Carbon Nanomaterials Unit Okinawa Institute of Science and Technology Graduate University Okinawa 904-0495 Japan; ^4^ Current address: Department of Chemistry University of Oxford Chemistry Research Laboratory Oxford OX1 3TA UK; ^5^ Current address: College of Chemistry and Chemical Engineering Central South University Changsha Hunan 410083 P. R. China

**Keywords:** dicyclopentaannelation, hexa-*peri*-hexabenzocoronene, low energy gap, not-fully benzenoid PAH, open-shell biradical

## Abstract

Synthesis of two dicyclopentaannelated hexa‐*peri*‐hexabenzocoronene (PHBC) regioisomers was carried out, using nonplanar oligoaryl precursors with fluorenyl groups: *m*PHBC **8** with two pentagons in the “*meta*”‐configuration was obtained as a stable molecule, while its structural isomer with the “*para*”‐configuration, *p*PHBC **16**, could be generated and characterized only in situ due to its high chemical reactivity. Both PHBCs exhibit low energy gaps, as reflected by UV‐vis‐NIR absorption and electrochemical measurements. They also show open‐shell singlet ground states according to electron paramagnetic resonance (EPR) measurements and density functional theory (DFT) calculations. The use of fully benzenoid HBC as a bridging moiety leads to significant singlet biradical characters (*y*_0_) of 0.72 and 0.96 for *m*PHBC **8** and *p*PHBC **16**, respectively, due to the strong rearomatization tendency of the HBC π‐system; these values are among the highest for planar carbon‐centered biradical molecules. The incorporation of fully unsaturated pentagons strongly perturbs the aromaticity of the parent HBC and makes the constituted benzene rings less aromatic or antiaromatic. These results illustrate the high impact of cyclopentaannelation on the electronic structures of fully benzenoid polycyclic aromatic hydrocarbons (PAHs) and open up a new avenue towards open‐shell PAHs with prominent singlet biradical characters.

## Graphical abstract

Incorporation of two fully unsaturated pentagons to the bay positions of hexa‐*peri*‐hexabenzocoronene (HBC) core gives access to two dicyclopentaannelated HBC regioisomers: *m*PHBC and *p*PHBC. Both compounds show decreased energy gaps compared with the parent HBC and exhibit singlet biradical ground state with prominent biradical character.

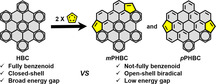

Polycyclic aromatic hydrocarbons (PAHs) with open‐shell electronic structures have drawn tremendous attention owing to their unique optical, magnetic and electronic properties, which make them promising candidates for use in organic (opto)electronic and spintronic devices.^[1]^ In addition to introducing extended zigzag edges, such as in anthenes,^[2]^ periacences^[3]^ and zethrenes,[^1f^, ^4^] annelation of fully unsaturated pentagons onto PAHs has proven to be another effective way to induce an open‐shell nature. To this end, several types of cyclopentannelated PAHs (PPAHs) have been constructed by fusing two indeno groups onto small PAHs, including naphthalene,^[5]^ anthracene,^[6]^ pyrene,^[7]^ perylene,^[8]^ bischrysene^[9]^ and corannulene.^[10]^ Furthermore, the addition of two methine bridges to the bay regions of PAHs has also been carried out to construct PPAHs (Scheme 1 A).^[11]^ However, in all of these examples, the two fully unsaturated pentagons (methine radical centers) are fused to not‐fully benzenoid PAHs, and most of them exhibit closed‐shell or open‐shell ground states with relatively small singlet biradical characters (*y*_0_<0.7). The incorporation of two unsaturated pentagons into fully benzenoid aromatic hydrocarbons is expected to generate PPAHs with a prominent open‐shell character, since larger numbers of Clar's aromatic π‐sextets exist in the open‐shell forms than in the closed‐shell forms. This strategy, however, has rarely been implemented to date, except for the indenofluorene family.[^1a^, ^12^]

**Scheme 1:**
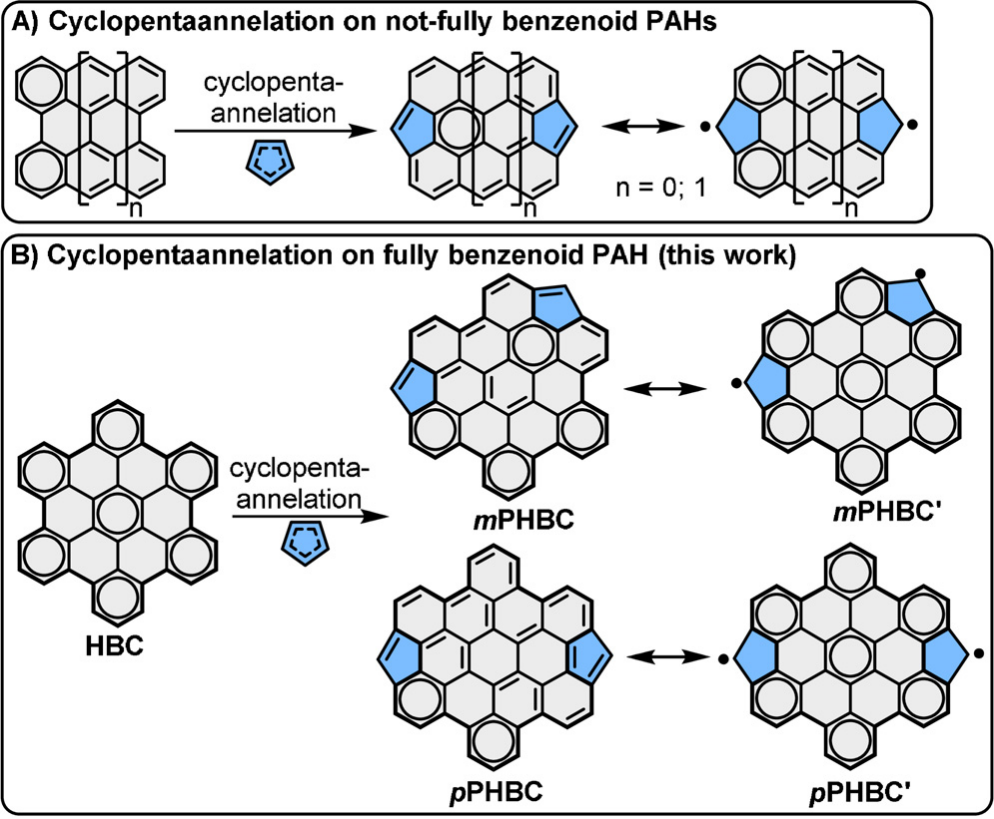
A) Representative examples of cyclopentaannelated, not‐fully benzenoid PAHs, i.e., dicyclopentaannelated perylene (*n*=0)^[11b]^ and dicyclopentaannelated bisanthene (*n*=1);^[11a]^ and B) dicyclopentaannelation on fully benzenoid HBC, giving access to *m*PHBC and *p*PHBC and their resonance between the Kekulé structure and open‐shell biradical form.

Hexa‐*peri*‐hexabenzocoronene (HBC) is a classic example of a fully benzenoid PAH,^[13]^ which has also been seen as a “superbenzene” due to its *D*_6*h*_ symmetry and close relation to benzene. In its ground state, HBC can be described as incorporating seven separated benzene rings, according to Clar's aromatic π‐sextet rule.^[14]^ The special electronic structure and high stability of HBC make it an attractive molecular model for studying how radicals delocalize their spin density and interact with each other through large conjugated π‐systems. For example, Wu's group investigated the synthesis of HBC‐quinone derivatives, which exhibited open‐shell biradical to multiradical features.^[15]^ In these molecules, the radical centers (oxygen atoms) are not directly involved in the π‐conjugated backbone. In comparison, incorporation of the radical centers into HBC could facilitate intramolecular radical conjugation and provide promising model molecules to investigate structure‐biradical property relationships. One question is how the regioisomerism influences the exchange interaction between radicals, but this remains unanswered.

Herein, we report our synthetic approach towards two pentagon‐fused HBC regioisomers with two five‐membered rings located on the bay regions in the “*meta”*‐configuration (*m*PHBC) and “*para”*‐configuration (*p*PHBC) (Scheme 1 B). Three and five more Clar's π‐sextets are gained in the open‐shell biradical forms of *m*PHBC and *p*PHBC, respectively, compared with their corresponding fully conjugated Kekulé structures, reflecting their preferences to exist as energetically more favorable biradicals. The relevant features, such as the low energy gap, open‐shell character and antiaromaticity, are comprehensively studied by UV‐vis‐NIR absorption and electrochemical experiments, variable‐temperature nuclear magnetic resonance (NMR) and electron paramagnetic resonance (EPR) measurements, and density functional theory (DFT) calculations.

To synthesize *m*PHBC **8** (Scheme 2 A), we first prepared 2,3‐dibromo‐1,4‐bis(trimethylsilyl)benzene (**2**) from 1,2‐dibromobenzene (**1**) by lithiation with lithium diisopropylamide (LDA), followed by nucleophilic substitution with trimethylsilyl chloride in 22 % yield. Then, twofold Suzuki coupling of **2** with 4‐*tert*‐butylphenylboronic acid provided terphenyl **3** in 77 % yield. Bromination of **3** with Br_2_ in a 1:1 (v/v) mixture of dichloromethane and methanol gave dibromoterphenyl **4** in 91 % yield. Next, Miyaura borylation on **4** afforded diborylated terphenyl **5** in 55 % yield. After Suzuki coupling with 4‐bromo‐9*H*‐fluoren‐9‐one, the key intermediate diketone **6** was obtained in 91 % yield. Subsequently, **6** was treated with mesityllithium (MesLi), followed by reduction with BF_3_⋅OEt_2_/Et_3_SiH to furnish bisfluorene **7** in 57 % yield over two steps. The cyclization of **7** was first attempted with ferric chloride (FeCl_3_) through a commonly used method for HBC synthesis,^[16]^ but only partial cyclization (three bonds were closed) was suggested by matrix‐assisted laser desorption/ionization time‐of‐flight mass spectrometry (MALDI‐TOF MS) analysis (see Figure S1). Increasing the reaction time and equivalents of FeCl_3_ had no effect on the result. Using 2,3‐dichloro‐5,6‐dicyano‐1,4‐benzoquinone (DDQ) as the oxidant in the presence of either methanesulfonic acid (MSA) or trifluoromethanesulfonic acid (TFMSA)^[17]^ gave complex reaction mixtures. Remarkably, using DDQ as an oxidant in the presence of MSA followed by the addition of TFMSA successfully provided the target compound *m*PHBC **8** (Figure S2), albeit in only 5 % yield after removing the major oxidized byproducts by silica gel chromatography. Although high‐quality crystals suitable for the single‐crystal X‐ray analysis could not be obtained, mainly resulting in the decomposition, the formation of **8** could be validated by NMR (vide infra) in addition to the MALDI‐TOF MS analysis.

**Scheme 2:**
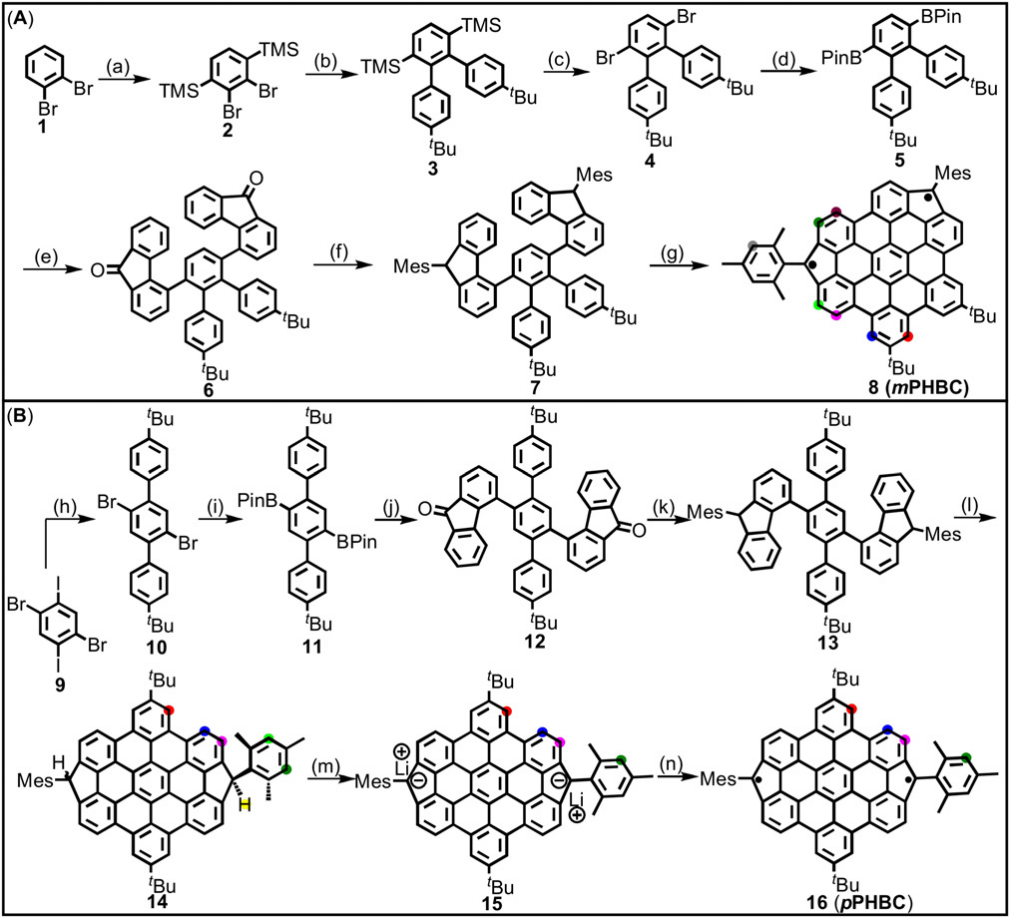
Synthesis of *m*PHBC **8** (A) and *p*PHBC **16** (B). Reagents and conditions: a) (1) LDA, THF, −78 °C; (2) TMSCl, r.t., 5 h, 22 % yield; b) 4‐*tert*‐butylphenylboronic acid, Pd(dppf)Cl_2_⋅CH_2_Cl_2_, K_3_PO_4_, DMF, 100 °C, 24 h, 77 % yield; c) Br_2_, DCM/MeOH, 25 °C, overnight, 91 % yield; d) (Bpin)_2_, Pd(dppf)Cl_2_⋅CH_2_Cl_2_, XPhos, K_2_CO_3_, DMF, 110 °C, 24 h, 55 % yield; e) 4‐bromo‐9*H*‐fluoren‐9‐one, Pd_2_(dba)_3_⋅CHCl_3_, XPhos, K_2_CO_3_, 1,4‐dioxane/H_2_O, 100 °C, 24 h, 91 % yield; f) (1) MesLi, THF, −78 °C to r.t., overnight; (2) BF_3_⋅OEt_2_, Et_3_SiH, DCM, r.t., overnight, 57 % yield; g) DDQ, MSA, r.t., 1 h, then TFMSA, r.t., 1 h, 5 % yield; h) 4‐*tert*‐butylphenylboronic acid, Pd(PPh_3_)_4_, K_2_CO_3_, 1,4‐dioxane/H_2_O, 90 °C, 24 h, 68 % yield; i) (1) *t*‐BuLi, THF, −78 °C, 2.5 h; (2) 2‐isopropoxy‐4,4,5,5‐tetramethyl‐1,3,2‐dioxaborolane, −78 °C to r.t., overnight, 29 % yield; j) 4‐bromo‐9*H*‐fluoren‐9‐one, Pd_2_(dba)_3_⋅CHCl_3_, XPhos, K_2_CO_3_, 1,4‐dioxane/H_2_O, 100 °C, 24 h, 79 % yield; k) (1) MesLi, THF, −78 °C to r.t., overnight; (2) BF_3_⋅OEt_2_, Et_3_SiH, DCM, r.t., 12 h, 66 % yield; l) FeCl_3_, DCM/MeNO_2_, r.t., 0.5 h, 86 % yield; m) *n*‐BuLi, THF, r.t.; n) I_2,_ r.t. LDA=lithium diisopropylamide, TMS=trimethylsilyl, dppf=1,1′‐bis(di‐phenylphosphino)ferrocene, dba=dibenzylideneacetone, Mes=mesityl, DDQ=2,3‐dichloro‐5,6‐dicyanobenzoquinone, MSA=methanesulfonic acid, TFMSA=trifluoromethanesulfonic acid, Bpin=boronic acid pinacol ester, THF=tetrahydrofuran, DMF=*N*,*N*‐dimethylformamide, DCM=dichloromethane.

The synthesis of *p*PHBC **16** was attempted in a similar way, starting from 1,4‐dibromo‐2,5‐diiodobenzene (**9**) (Scheme 2 B). After Suzuki coupling of **9** with 4‐*tert*‐butylphenylboronic acid and borylation, terphenyl borate ester **11** was synthesized in 29 % yield. Then, Suzuki coupling with 4‐bromo‐9*H*‐fluoren‐9‐one provided diketone **12** in 79 % yield, which was treated with MesLi and reduced by BF_3_⋅OEt_2_/Et_3_SiH to provide **13** in 66 % yield. Different from the synthesis of *m*PHBC **8**, the treatment of **13** with DDQ as the oxidant and MSA as the acid, followed by the addition of TFMSA, resulted in messy reaction mixtures without assignable peaks in the MS spectrum. In this case, however, the use of FeCl_3_ provided intermediate HBC **14** in 86 % yield. Further dehydrogenation/aromatization was attempted using different conditions, including the application of DDQ or *p*‐chloranil as an oxidant or ^*t*^BuOK/DMF, which have been proven effective for the aromatization of similar precursors.^[18]^ All these conditions were unsuccessful, and only oxidized (i.e., oxygen containing) *p*PHBC could be detected by MALDI‐TOF MS (see Figures S4,S5). Since *p*PHBC **16** could not be isolated, presumably due to its high reactivity, we turned to an in situ generation and characterization of *p*PHBC **16**, employing a sequence of deprotonation with *n*‐BuLi and oxidation with iodine.

As demonstrated in Figure 1 A, the aromatic region of the ^1^H NMR spectrum of *m*PHBC **8** measured in CD_2_Cl_2_ at 273 K clearly shows four doublet and three singlet peaks, which could be fully assigned via two‐dimensional NMR (see Figures S30,S31). With increasing temperature, these sharp peaks broadened, decreased in intensity and finally disappeared at 373 K. We ascribe this observation to the presence of a thermally excited triplet state due to a small singlet‐triplet energy gap, as has been commonly observed in other open‐shell systems,[^3a^, ^9a^, ^11a^, ^19^] although partial thermal decomposition of **8** cannot be excluded. As illustrated in Figure 1 B, the ^1^H NMR spectrum of HBC derivative **14** in [D_8_]THF exhibits two sets of fully assignable peaks due to the existence of its *cis*‐/*trans*‐isomers. After the addition of 2.0 equiv. of *n*‐BuLi to this solution, the peaks belonging to the methine bridges disappeared, and the whole spectrum became simpler, in agreement with the highly symmetric structure of the resulting dianion **15**. In the following step, 2.0 equiv. of I_2_ were added to oxidize dianion **15**, which caused the disappearance of the sharp peaks. Even if the temperature was decreased to 213 K, no well‐resolved peak could be observed from the NMR spectrum. After the in situ synthesis, MALDI‐TOF MS analysis showed oxidized species of *p*PHBC **16** without any other side products, such as the addition of *n*‐butyl or iodo groups (see Figure S6), which supported the formation of **16** (vide infra for the further characterizations of the in situ generated **16**).

**Figure 1:**
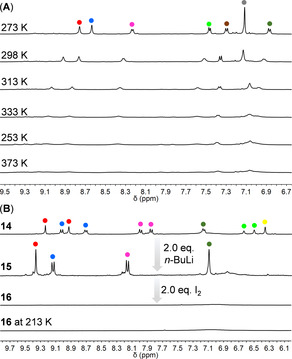
A) Aromatic region of variable‐temperature ^1^H NMR spectra of *m*PHBC **8** (500 MHz, 273 K and 298 K in CD_2_Cl_2_, 313–373 K in C_2_D_2_Cl_4_); B) aromatic region of ^1^H NMR spectra of HBC precursor **14**, dianion **15** (300 MHz) and *p*PHBC **16** at 298 K and 213 K (500 MHz, [D_8_]THF). The height of the solvent residual peaks was normalized for comparison.

The UV‐vis‐NIR absorption spectra (Figure 2 A) of *m*PHBC **8** and in situ generated *p*PHBC **16** exhibit intense absorption bands in the long wavelength region centered at 682 nm and 770 nm, respectively, together with weak tailing into the 800–1200 nm region. The longest absorption wavelengths at approximately 1075 nm (**8**) and 1150 nm (**16**) originate from the forbidden HOMO→LUMO transition according to TD‐DFT calculations (UB3LYP/6‐31G(d, p), HOMO→LUMO transitions of *m*PHBC **8** and *p*PHBC **16** are calculated at 1012 nm (*f*=0.0761) and 1025 nm (*f*=0.0598), respectively). These features are starkly different from the spectrum of pristine HBC with the main UV‐vis absorption wavelength below 400 nm (see Figure S7). The half‐life times of *m*PHBC **8** and *p*PHBC **16** in degassed solutions in the dark under an inert atmosphere were estimated to be 19 days and 7 h, respectively, from the decays of their absorbance at 682 nm and 770 nm (see Figures S9, S10, S14, S15). The cyclic voltammetry (CV) of *m*PHBC **8** (Figure 2 B) gave two reversible oxidation and reduction peaks, with half‐wave potentials at *E*_1/2_^ox^=0.42 V and 0.79 V and at *E*_1/2_^red^=−0.88 V and −1.16 V with reference to Fc/Fc^+^. The HOMO and LUMO energy levels were accordingly calculated to be −5.14 eV and −3.86 eV, respectively. The low LUMO energy level indicates that *m*PHBC **8** is potentially a good electron acceptor. Treating *m*PHBC **8** with hydrazine solution results in the formation of a reduction product containing two hydrogens more than *m*PHBC **8** (see Figure S3), which has not been accessible by cyclodehydrogenation of compound **7**.

**Figure 2:**
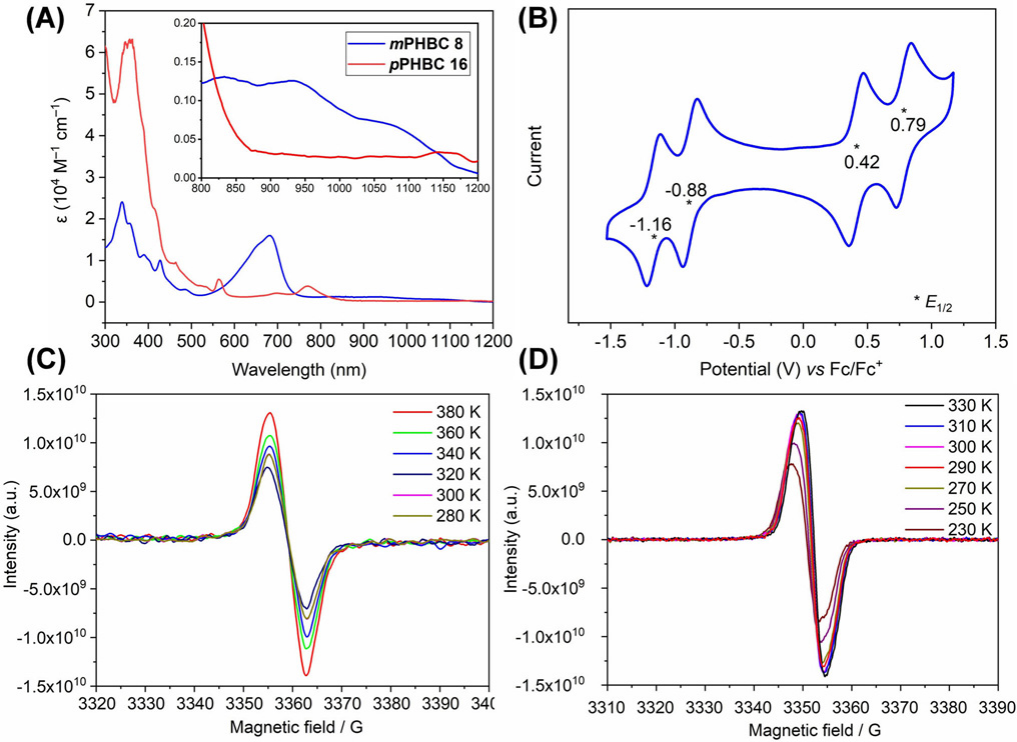
A) UV‐vis‐NIR absorption of *m*PHBC **8** in DCM and in situ generated *p*PHBC **16** in THF under an argon atmosphere with a concentration of 10^−5^ M; the inset shows the magnified long‐wavelength‐absorption region. B) Cyclic voltammogram of *m*PHBC **8** in dry *o*‐dichlorobenzene with 0.1 M *n*‐Bu_4_NPF_6_ as an electrolyte at room temperature with Fc/Fc^+^ as reference. C and D) Variable‐temperature EPR spectra of *m*PHBC **8** in the solid state and in situ generated *p*PHBC **16** in a THF solution.

EPR measurements were performed on *m*PHBC **8** in the solid state and in situ generated *p*PHBC **16** in a THF solution at different temperatures (Figure 2 C, D). Both *m*PHBC **8** and *p*PHBC **16** exhibit unresolved broad EPR signals. The intensity of the EPR signal increases at higher temperatures, which reflects the existence of thermally accessible triplet states for both **8** and **16**. DFT calculations were conducted at the B3LYP/6–31G(d, p) level of theory to further understand the electronic properties of the PHBCs. Using the occupation numbers of the spin‐unrestricted Hartree–Fock natural orbitals,^[20]^ *m*PHBC **8** appears to have a moderate singlet biradical character (*y*_0_) of 0.72, while *p*PHBC **16** possesses a much larger *y*_0_ value of 0.96. This can be rationalized by drawing resonance structures of PHBCs (Scheme 1 B). It appears that two more Clar's π‐sextets are destroyed for *p*PHBC **16** when preserving the fully conjugated Kekulé structure, which makes the biradical form of **16** energetically more favorable. Spin density distribution calculations indicate that unpaired electrons are mainly delocalized on the rim of PHBCs, with the methine bridges at the bay regions having the highest spin densities (Figure 3 A,B), while the spin densities of the adjacent carbons are relatively small. In addition, the calculations show that the singlet biradical forms of *m*PHBC **8** and *p*PHBC **16** have lower energies than those of both their closed‐shell and open‐shell triplet biradical forms (see Table S1). The singlet‐triplet energy gaps (Δ*E*_S‐T_) were calculated to be −1.80 kcal mol^−1^ and −0.18 kcal mol^−1^ for *m*PHBC **8** and *p*PHBC **16**, respectively, indicating a singlet biradical ground state of both molecules, which agrees with the variable‐temperature NMR and EPR measurements.

**Figure 3:**
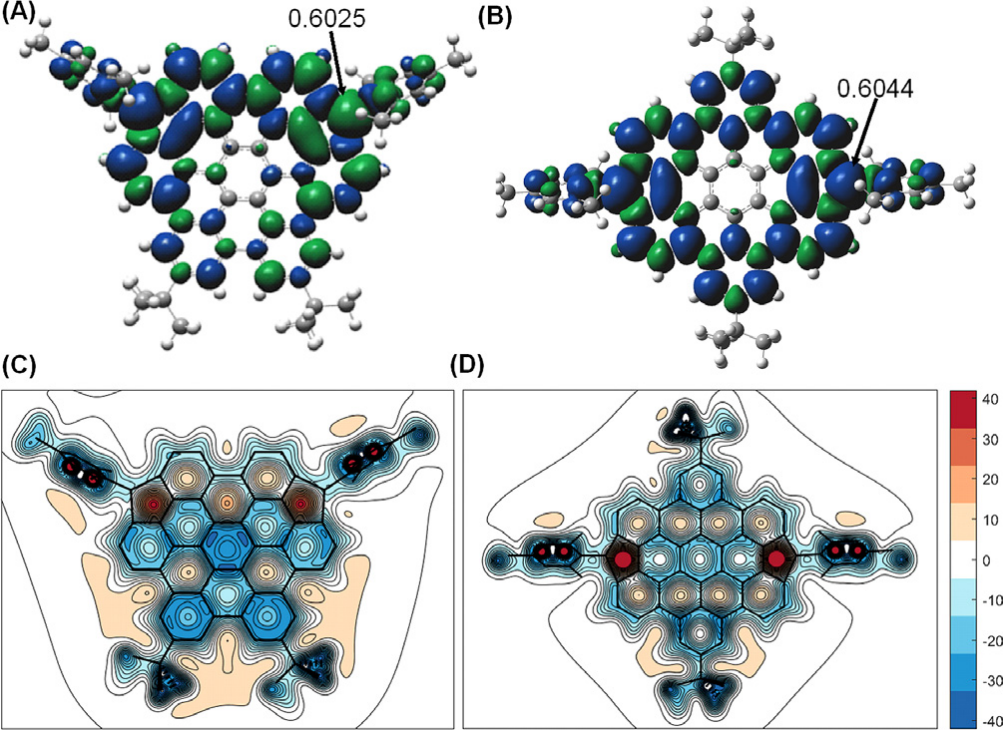
Spin density distribution of the triplet diradical state of A) *m*PHBC **8** and B) *p*PHBC **16**, the numbers indicate spin densities on these positions; XY NICS(1) maps calculated for C) *m*PHBC **8** and D) *p*PHBC **16**. GIAO shieldings (shown with negative values) were calculated at the B3LYP/6‐31G(d, p) level of theory 1 Å above the molecular plane.

The effects of cyclopentaannelation on the aromaticity of the HBC core in *m*PHBC **8** and *p*PHBC **16** were visualized by probing the gauge‐independent atomic orbital (GIAO) shielding 1 Å above the molecular planes (Figure 3 C,D). The resulting XY nucleus‐independent chemical shift (NICS) (1) maps reveal that in *m*PHBC **8**, the five‐membered ring zones are strongly deshielded (NICS(1)=39), which also causes deshielding of the adjacent two benzene rings (on top, NICS(1)=16) connecting these two five‐membered rings, while the other five benzene rings remain aromatic (NICS(1)=−9 to −22). This observation was experimentally confirmed by NMR measurements showing high‐field shifts of the protons (*δ*=7.32 ppm, 6.89 ppm) on the benzene rings fused to the five‐membered rings (Figure 1 A). The XY NICS(1) map of *p*PHBC **16** displays a different pattern. The deshielding effect is particularly large above two five‐membered rings (NICS(1)=53) and noticeably weaker above the other seven benzene rings (NICS(1)=−3 to 14), indicating more localized antiaromaticity of *p*PHBC.

In summary, we designed two dicyclopenta‐fused HBCs, i.e., *m*PHBC **8** and *p*PHBC **16**, and successfully obtained the former and in situ generated the latter. The synthetic routes involved the cyclodehydrogenation of adequately designed precursors with one pair of preinstalled fluorenyl groups as key steps. Both PHBC isomers exhibit remarkable singlet biradical ground‐state features, as proven by variable‐temperature NMR and EPR experiments, together with DFT calculations. The most characteristic features of the PHBCs are their low energy gaps, as indicated by the long wavelength optical absorption and CV measurements, and the disruption of the Kekulé structures of the parent HBC. The prominent singlet biradical character found for the PHBCs can presumably be ascribed to the strong tendency of the HBC core to locally sustain the fully benzenoid form, leading to partial localization of spin over the methine carbons. This design strategy may afford access to additional unique cyclopentaannelated PAHs based on other existing fully benzenoid PAHs that are abundant in the literature. These cyclopentaannelated PAHs could find potential applications in optoelectronics, spintronics and quantum information technology.

## Conflict of interest

The authors declare no conflict of interest.

## Supporting information

As a service to our authors and readers, this journal provides supporting information supplied by the authors. Such materials are peer reviewed and may be re‐organized for online delivery, but are not copy‐edited or typeset. Technical support issues arising from supporting information (other than missing files) should be addressed to the authors.

Supplementary
