# Supplementary material for: Dicyclopentaannelated Hexa‐peri‐hexabenzocoronenes with a Singlet Biradical Ground State
Source: Angew Chem Int Ed Engl. 2021 Apr 7;60(20):11300–4. doi: 10.1002/anie.202102932 (PMC8251543; doi:10.1002/anie.202102932)
Supplement: Supplementary file 1 — Supplementary [file ANIE-60-11300-s001.pdf]

Angewandte Chemie
Eine Zeitschrift der Gesellschaft Deutscher Chemiker

Supporting Information

Dicyclopentaannelated Hexa-*peri*-hexabenzocoronenes with a Singlet Biradical Ground State

Qiang Chen, Martin Baumgarten, Manfred Wagner, Yunbin Hu, Ian Cheng-Yi Hou, Akimitsu Narita, and Klaus Müllen**

anie_202102932_sm_miscellaneous_information.pdf

Table of Contents

Table of Contents S1
1. General Methods S2
2. Synthetic Details S3
3. Supplementary MS Spectra S8
4. Supplementary UV-vis Absorption Spectra S11
5. Theoretical Calculations S14
6. NMR Spectra S16
7. References S32
8. Cartesian Coordinates and Spin Densities S33

1. General Methods

All reactions working with air- or moisture-sensitive compounds were carried out under argon atmosphere using standard Schlenk line techniques. Unless otherwise noted, all starting materials were purchased from commercial sources and used without further purification. All other reagents were used as received. Thin layer chromatography (TLC) was done on silica gel coated aluminum sheets with F254 indicator and column chromatography separation was performed with silica gel (particle size 0.063 – 0.200 mm). Melting points were determined on a Büchi hot stage apparatus and were uncorrected. Nuclear Magnetic Resonance (NMR) spectra were recorded using Bruker DPX 300 and Bruker DPX 500 MHz NMR spectrometers. Chemical shifts (δ) were expressed in ppm relative to the residual of solvents (CD_2Cl_2, ^{1}H: 5.32 ppm, ^{13}C: 53.84 ppm; $C_2D_2Cl_4$, ^{1}H: 6.00 ppm, ^{13}C: 73.78 ppm; THF-d_8, ^{1}H: 3.58 ppm, ^{13}C: 67.57 ppm. Abbreviations: s = singlet, d = doublet, dd = double doublet, t = triplet, m = multiplet). Coupling constants (*J*) were recorded in Hertz. Accurate mass measurements were performed on a SYNAPT G2-Si high definition Q-TOF mass spectrometer (Waters Corp., Manchester, UK) by matrix-assisted laser desorption/ionization (MALDI). The instrument was operated at a resolution of 20 000 ($M/\Delta M$) and calibrated by clusters of red phosphorous in a mass range of 100 to 8000 Da. Data processing was done with MassLynx software V4.1. Or on a G6545A Q-ToF (Agilent GmbH, Waldbronn, Germany) with atmospheric pressure chemical ionization (APCI) mass spectrometer. UV-*vis* absorption spectra were recorded on a Perkin-Elmer Lambda 900 spectrometer at room temperature using a 10 mm quartz cell. Cyclic voltammetry (CV) measurements were performed on a GSTAT-12 in a three-electrode cell in *o*-dichlorobenzene solution of *n*-Bu_4NPF_6 (0.1 M) at a scan rate of 50 mV/s at room temperature. A silver wire, a Pt wire and a glassy carbon electrode were used as the reference electrode, the counter electrode, and the working electrode, respectively. EPR spectra were recorded in solid or diluted and oxygen-free solutions by using a Bruker Xband spectrometer ESP300 E, equipped with an NMR gauss meter (Bruker ER035), a frequency counter (Bruker ER 041 XK) and a variable temperature control continuous flow N2 cryostat (Bruker B-VT 2000).

2. Synthetic Details

(2,3-dibromo-1,4-phenylene)bis(trimethylsilane) (**2**)[1]

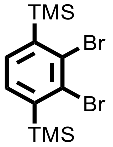

To a 250-mL round bottom flask charged with a stirring bar was added 20 mL of dry tetrahydrofuran. After cooling to –78 °C under argon atmosphere, *n*-butyllithium (82.4 mL, 0.13 mol, 1.6 M in *n*-hexane) and *N*,*N*-diisopropylamine (13.4 g, 0.132 mol) were added. After 15 min of stirring, the resulting mixture was slowly added to a solution of 1,2-dibromobenzene (**1**) (13.0 g, 55.3 mmol) and trimethylsilyl chloride (14.34 g, 132.0 mmol) in 30 mL of THF at –78 °C. The reaction mixture was stirred at this temperature for 5 h and quenched with 1 N sulfuric acid solution. The resulting mixture was diluted with water and extracted with diethyl ether (80 mL) for three times. The combined organic layers were dried over $MgSO_4$ and evaporated. The residue was dissolved in a mixture of acetone and methanol (1:1, 10 mL) and crystallized at –30 °C over several days to afford the title compound (4.7 g, 22%) as a colorless solid. All spectral data was in accordance with that reported in the literature.[1] Mp: 72.1 – 73.7 °C; ^{1}H NMR (300 MHz, CD_2Cl_2, 298 K) *δ* 7.35 (s, 2H), 0.39 (s, 18H); ^{13}C NMR (75 MHz, CD_2Cl_2, 298 K) *δ* 146.36, 134.61, 133.71, ‑0.19.

(4,4''-di-*tert*-butyl-[1,1':2',1''-terphenyl]-3',6'-diyl)bis(trimethylsilane) (**3**)[2]

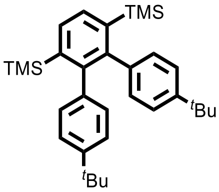

To a 250-mL Schlenk flask charged with a stirring bar was added (2,3-dibromo-1,4-phenylene)bis(trimethylsilane) (**2**) (6.0 g, 16 mmol), 4-*tert*-butylphenylboronic acid (11.2 g, 63.1 mmol), K_3PO_4 (26.8 g, 126 mmol), and Pd(dppf)Cl_2·CH_2Cl_2 (1.29 g, 1.58 mmol). After evacuation and back filling with argon for three times, dimethylformamide (80 mL) and water (10 mL) were added. The solution was further degassed by bubbling through argon for 10 min, and heated at 100 °C for 24 h. The black reaction mixture was cooled to room temperature and diluted with 300 mL of ethyl acetate. The organic phase was washed with water (50 mL) for 6 times and then brine (50 mL), dried over $MgSO_4$ and evaporated. The residue was purified by silica gel column chromatography (*n*-hexane:dichloromethane = 30:1) to give the title compound (6.0 g, 77% yield) as a white solid. All spectral data was in accordance with that reported in the literature.[2] Mp: 249.4 – 250.3 °C; ^{1}H NMR (300 MHz, CD_2Cl_2, 298 K) *δ* 7.59 (s, 2H), 7.07 (d, *J* = 8.2 Hz, 4H), 6.84 (d, *J* = 8.2 Hz, 4H), 1.21 (s, 19H), –0.08 (s, 19H); ^{13}C NMR (75 MHz, CD_2Cl_2, 298 K) *δ* 149.59, 148.12, 140.52, 140.04, 132.97, 131.18, 123.92, 34.73, 31.60, 0.68; HRMS (APCI) [M$^+$] Calculated for $C_{32}H_{46}Si_2$: 486.3133; Found 486.3138.

1,4-dibromo-2,3-di-(4-*tert*-butylphenyl)benzene (**4**)

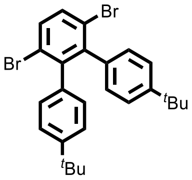

Br_2 (1.89 mL, 36.9 mmol) was added dropwise to a solution of (4,4''-di-*tert*-butyl-[1,1':2',1''-terphenyl]-3',6'-diyl)bis(trimethylsilane) (**3**) (6.9 g, 12 mmol) dissolved in a mixture of dichloromethane (200 mL) and methanol (100 mL). The reaction mixture was stirred at 25 °C for overnight. Saturated aqueous Na_2SO_3 solution (100 mL) was added to the reaction mixture and the aqueous phase was extracted with dichloromethane (100 mL) for three times. The combined organic layers were washed with brine (50 mL), dried over Na_2SO_4, and concentrated under reduced pressure. The crude was recrystallized from methanol to afford the title compound (5.6 g, 91% yield) as a white solid. Mp: 172.3 – 173.9 °C; ^{1}H NMR (300 MHz, CD_2Cl_2, 298 K) *δ* 7.54 (s, 1H), 7.14 (d, *J* = 8.3 Hz, 2H), 6.86 (d, *J* = 8.3 Hz, 2H), 1.22 (s, 9H); ^{13}C NMR (75 MHz, CD_2Cl_2, 298 K) *δ* 150.48, 144.87, 137.75, 133.09, 130.11, 124.66, 123.65, 34.84, 31.50; HRMS (APCI) [M$^+$] Calculated for $C_{26}H_{28}Br_2$: 498.0552; Found 498.0558.

2,3-di-(4-*tert*-butylphenyl)-1,4-bis(4,4,5,5-tetramethyl-1,3,2-dioxaborolan-2-yl)benzene (**5**)

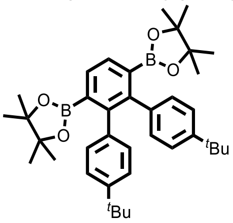

A 250-mL Schlenk flask equipped with a magnetic stirring bar was charged with 1,4-dibromo-2,3-di-(4-*tert*-butylphenyl)benzene (**4**) (5.6 g, 11 mmol), bis(pinacolato)diboron (8.5 g, 34 mmol), XPhos (0.534 g, 1.12 mmol), and K_2CO_3 (9.29 g, 67.2 mmol). The flask was evacuated and backfilled with argon for three times. 1,4-Dioxane (120 mL) was added and the resulting mixture was degassed by bubbling with argon for 15 min. Under argon atmosphere, Pd(dppf)Cl_2·CH_2Cl_2 (457 mg, 0.560 mmol) was added and the reaction mixture was stirred at 110 °C for 24 h. After cooling to room temperature, water (50 mL) was added, and the aqueous layer was extracted with dichloromethane (50 mL) for three times. The combined organic layers were washed with brine, dried over Na_2SO_4, and filtered. After removing the solvent in vacuo, the residue was purified by silica gel column chromatography (*n*-hexane:ethyl acetate = 20:1) to give the title compound (3.6 g, 55% yield) as a white solid. Mp: 257.3 – 258.8 °C; ^{1}H NMR (300 MHz, CD_2Cl_2, 298 K) δ 7.53 (s, 2H), 7.15 (d, J = 8.3 Hz, 4H), 6.93 (d, J = 8.3 Hz, 4H), 1.26 (s, 19H), 1.07 (s, 24H); ^{13}C NMR (75 MHz, CD_2Cl_2, 298 K) δ 149.39, 145.49, 140.06, 131.66, 130.62, 124.38, 84.03, 34.76, 31.66, 24.90; HRMS (APCI) [M+H$^+$] Calculated for $C_{38}H_{52}B_2O_4$: 595.4124; Found 595.4142.

4,4'-(4,4''-di-*tert*-butyl-[1,1':2',1''-terphenyl]-3',6'-diyl)bis(9*H*-fluoren-9-one) (**6**)

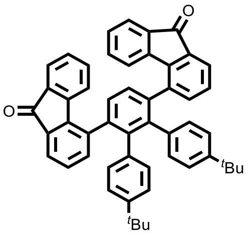

To a 100-mL Schlenk tube charged with a magnetic stirring bar was added 2,3-di-(4-*tert*-butylphenyl)-1,4-bis(4,4,5,5-tetramethyl-1,3,2-dioxaborolan-2-yl)benzene (**5**) (1.88 g, 3.16 mmol), 4-bromo-9*H*-fluoren-9-one (1.80 g, 6.95 mmol), $Pd_2(dba)_3$·$CHCl_3$ (289 mg, 0.316 mmol), XPhos (300 mg, 0.629 mmol) and K_2CO_3 (2.620 g, 18.96 mmol). The reaction tube was evacuated and backfilled with argon for three times before degassed 1,4-dioxane (40 mL) and H_2O (10 mL) were added. After bubbling with argon for 10 min, the Schlenk tube was then sealed and heated at 100 °C for 24 h. After cooling to room temperature, the reaction mixture was extracted with dichloromethane (50 mL) for three times. The combined organic fractions were washed with brine, dried over Na_2SO_4, and filtered, and the solvent was removed in vacuo. The residue was purified by silica gel column chromatography (*n*-hexane:ethyl acetate = 10:1) to give the title compound (2.01 g, 91%) as yellow solid. Mp: 326.7 – 328.5 °C; ^{1}H NMR (500 MHz, $C_2D_2Cl_4$, 413 K) δ 7.71 (d, J = 7.2 Hz, 2H), 7.65 (d, J = 7.4 Hz, 2H), 7.57 (d, J = 7.2 Hz, 2H), 7.41 (t, J = 7.5 Hz, 2H), 7.36 – 7.29 (m, 3H), 7.26 (d, J = 6.9 Hz, 1H), 7.24 – 7.13 (m, 3H), 6.98 (t, J = 9.8 Hz, 1H), 6.86 (d, J = 6.4 Hz, 4H), 6.76 (d, J = 8.2 Hz, 4H), 1.14 (s, 18H); ^{13}C NMR (126 MHz, $C_2D_2Cl_4$, 413 K) δ 192.69, 150.21, 144.68, 144.60, 142.01, 141.77, 140.44, 140.11, 138.00, 137.05, 137.00, 136.31, 134.77, 134.66, 133.94, 133.51, 132.18, 132.01, 128.98, 128.41, 128.12, 128.01, 124.44, 123.69, 122.69, 34.04, 30.86; HRMS (APCI) [M$^+$] Calculated for $C_{52}H_{42}O_2$: 698.3179; Found 698.3185.

4,4'-(4,4''-di-*tert*-butyl-[1,1':2',1''-terphenyl]-3',6'-diyl)bis(9-mesityl-9*H*-fluorene) (**7**)

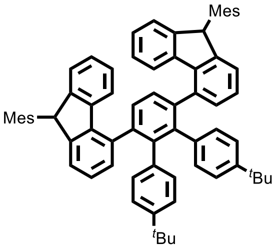

n-BuLi (15.6 mL, 25 mmol, 1.6 M in *n*-hexane) was added to a solution of 2-bromomesitylene (5.0 g, 25 mmol) in anhydrous tetrahydrofuran (30 mL) at –78 °C over a period of 30 min. After the addition, the mixture was stirred at this temperature for 2 h. Then, thus prepared solution of mesityllithium was added dropwise to a solution of 4,4'-(4,4''-di-*tert*-butyl-[1,1':2',1''-terphenyl]-3',6'-diyl)bis(9*H*-fluoren-9-one) (**6**) (2.0 g, 2.9 mmol) in tetrahydrofuran (100 mL) at –78 °C. After the addition, the temperature was increased to room temperature and the mixture was stirred overnight. The reaction was quenched by addition of water, and the mixture was then extracted with ethyl acetate, dried over Na_2SO_4 and evaporated. The residue was dissolved in dry dichloromethane (250 mL), to which BF_3·OEt_2 (4.3 g, 30 mmol) and Et_3SiH (3.6 g, 31 mmol) were added, and the resulting mixture was stirred at room temperature overnight. The reaction was quenched with aqueous Na_2CO_3 solution. The organic layer was separated and the water aqueous phase was extracted

with dichloromethane (100 mL) for three times. The organic phases were combined, washed with brine, dried over Na_2SO_4 and evaporated. The residue was purified by column chromatography (*n*-hexane:ethyl acetate = 10:1) and recrystallized from dichloromethane/methanol to give the title compound (1.5 g, 57% yield) as white solid. Mp: 284.1 – 285.7 °C; ^{1}H NMR (500 MHz, $C_2D_2Cl_4$, 413 K) δ 7.73 – 7.52 (m, 3H), 7.48 – 7.35 (m, 1H), 7.35 – 7.20 (m, 7H), 7.15 (tt, *J* = 9.3, 4.6 Hz, 2H), 7.09 (d, *J* = 7.8 Hz, 2H), 7.06 – 6.98 (m, 2H), 6.86 – 6.73 (m, 8H), 6.72 – 6.57 (m, 2H), 5.74 – 5.22 (m, 2H), 2.95 – 2.57 (m, 6H), 2.50 – 2.14 (m, 6H), 1.33 – 1.23 (d, *J* = 8.5 Hz, 2H), 1.13 (s, 18H), 0.94 – 0.75 (m, 4H); ^{13}C NMR (126 MHz, $C_2D_2Cl_4$, 413 K) δ 148.02, 147.88, 147.80, 147.31, 142.08, 141.93, 141.50, 141.39, 141.32, 140.16, 140.01, 138.66, 138.37, 137.93, 137.65, 137.50, 137.28, 136.56, 135.51, 134.10, 133.95, 130.48, 130.38, 130.33, 130.28, 129.43, 129.37, 128.68, 128.58, 128.53, 128.39, 126.42, 126.34, 126.24, 126.07, 125.67, 123.65, 123.55, 122.83, 122.54, 122.09, 121.96, 49.51, 33.67, 30.85, 20.94, 20.35, 18.50, 18.45, 18.28; HR (APCI) [M$^+$] Calculated for $C_{70}H_{66}$: 906.5159; Found 906.5217.

*m*PHBC **8**

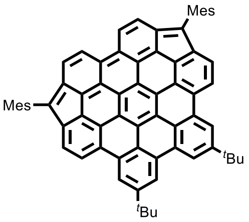

To a degassed solution of 4,4'-(4,4''-di-*tert*-butyl-[1,1':2',1''-terphenyl]-3',6'-diyl)bis(9-mesityl-9*H*-fluorene) (**7**) (50 mg, 0.055 mmol) and 2,3-dichloro-5,6-dicyano-1,4-benzoquinone (100 mg, 0.441 mmol) in dry dichloromethane (10 mL) was added methanesulfonic acid (1.0 mL), and the resulting mixture was stirred at room temperature for 1 h. Then, trifluoromethanesulfonic acid (1.0 mL) was added and the reaction mixture was continuously stirred for another 1 h. The mixture was poured into cold water and extracted with dichloromethane. The organic phase was separated, washed with brine, dried over Na_2SO_4 and passed through a short plug of silica gel. The resulting dark green solution was evaporated and residue was purified by silica gel column chromatography (dichloromethane:*n*-hexane = 1:3) to give the title compound (2.5 mg, 5% yield) as dark green solid. Mp: >400, decomposition; ^{1}H NMR (300 MHz, CD_2Cl_2, 298 K) δ 8.88 (s, 2H), 8.73 (s, 2H), 8.29 (d, *J* = 7.3 Hz, 2H), 7.48 (s, 2H), 7.32 (d, *J* = 9.3 Hz, 2H), 7.09 (s, 4H), 6.89 (d, *J* = 7.9 Hz, 2H), 2.42 (s, 21H), 1.71 (s, 17H); ^{13}C NMR (75 MHz, CD_2Cl_2) δ 150.69, 148.87, 138.21, 137.57, 137.22, 132.11, 131.41, 129.14, 121.36, 119.78, 36.20, 32.14, 21.64, 21.51; HRMS (MALDI-TOF) [M$^+$] Calculated for $C_{70}H_{52}$: 892.4064; Found 892.4037; UV/Vis (dichloromethane): λ_{max} (ε) = 682 nm (16008 $M^{-1}cm^{-1}$), 484 nm (3047 $M^{-1}cm^{-1}$), 427 nm (100478 $M^{-1}cm^{-1}$), 390 nm (11379 $M^{-1}cm^{-1}$), 357 nm (18942 $M^{-1}cm^{-1}$), 339 nm (24055 $M^{-1}cm^{-1}$).

1,4-dibromo-2,5-di-(4-*tert*-butylphenyl)benzene (**10**)[3]

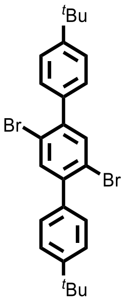

To a 250-mL Schlenk flask charged with a magnetic stirring bar was added 1,4-dibromo-2,5-diiodobenzene (5.00 g, 10.3 mmol), 4-*tert*-butylphenylboronic acid (3.56 g, 20.0 mmol), K_2CO_3 (8.54 g, 61.8 mmol) and $Pd(PPh_3)_4$ (581 mg, 0.503 mmol). The flask was evacuated and backfilled with argon for three times before 1,4-dioxane (100 mL) and water (25 mL) were added. The mixture was further degassed by bubbling with argon for 15 minutes. Then the reaction was heated at 90 °C for 24 h. After cooling to room temperature, the mixture was poured into water and extracted with dichloromethane for three times. The combined organic phases were washed by brine and dried over anhydrous $MgSO_4$. The solvent was removed under vacuum and the residue was purified by silica gel column chromatography (*n*-hexane) to give the title compound as a white solid (3.50 g, 68% yield). All spectral data was in accordance with that reported in the literature.[3] Mp: 261.2 – 262.1 °C; ^{1}H NMR (300 MHz, CD_2Cl_2, 298 K) δ 7.65 (s, 2H), 7.49 (d, *J* = 8.2 Hz, 4H), 7.40 (d, *J* = 8.2 Hz, 4H), 1.38 (s, 18H); ^{13}C NMR (75 MHz, CD_2Cl_2, 298 K) δ 151.81, 143.15, 137.10, 135.90, 129.52, 125.68, 121.83, 35.15, 31.64; HRMS (APCI) [M$^+$] Calculated for $C_{26}H_{28}Br_2$: 498.0552; Found 498.0554.

2,5-di-(4-*tert*-butylphenyl)-1,4-bis(4,4,5,5-tetramethyl-1,3,2-dioxaborolan-2-yl)benzene (**11**)

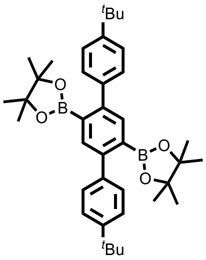

To a solution of 1,4-dibromo-2,5-di-(4-*tert*-butylphenyl)benzene (**10**) (2.0 g, 4.0 mmol) dissolved in anhydrous tetrahydrofuran (50 mL) was added dropwise *t*-BuLi (8.23 mL, 14 mmol, 1.7 M in *n*-hexane) at ‐78 °C under a nitrogen atmosphere. The reaction was then stirred at ‐78 °C for 2.5 h. 2-Isopropoxy-4,4,5,5-tetramethyl-1,3,2-dioxaborolane (3.72 g, 20.0 mmol) was then added slowly under argon atmosphere. The resulting solution was allowed to warm slowly to room temperature and stirred overnight. The reaction was quenched with deionized water (50 mL) and extracted with ethyl acetate (50 mL) for three times. The organic phases were combined, washed with brine, dried over Na_2SO_4 and evaporated. The residue was purified by silica gel column chromatography (*n*-hexane:ethyl acetate = 30:1) to give the title compound (700 mg, 29% yield) as white solid. Mp: 172.6 – 173.7 °C; ^{1}H NMR (300 MHz, CD_2Cl_2, 298 K) δ 7.68 (s, 1H), 7.43 (d, J = 8.3 Hz, 2H), 7.36 (d, J = 8.4 Hz, 2H), 1.37 (s, 9H), 1.21 (s, 12H); ^{13}C NMR (75 MHz, CD_2Cl_2, 298 K) δ 150.60, 145.28, 140.43, 135.27, 129.25, 125.38, 84.39, 34.96, 31.72, 24.97; HRMS (APCI) [M+H$^+$] Calculated for $C_{38}H_{52}B_2O_4$: 595.4124; Found 595.4136.

4,4'-(4,4''-di-*tert*-butyl-[1,1':4',1''-terphenyl]-2',5'-diyl)bis(9*H*-fluoren-9-one) (**12**)

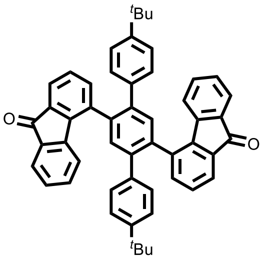

To a 50-mL Schlenk tube charged with a magnetic stirring bar was added 2,5-di-(4-*tert*-butylphenyl)-1,4-bis(4,4,5,5-tetramethyl-1,3,2-dioxaborolan-2-yl)benzene (**11**) (700 mg, 1.18 mmol), 4-bromo-9*H*-fluoren-9-one (763 mg, 2.94 mmol), and $Pd_2(dba)_3{\cdot}CHCl_3$ (216 mg, 0.236 mmol), XPhos (225 mg, 0.472 mmol) and K_2CO_3 (1.957 g, 14.16 mmol). Then the tube was evacuated and backfilled with argon for three times before degassed 1,4-dioxane (20 mL) and water (5 mL) were added. The Schlenk tube was then sealed and heated at 100 °C for 24 h. After cooling to room temperature, the mixture was extracted with ethyl acetate (50 mL) for three times. The combined organic fractions were washed with brine, dried over Na_2SO_4, filtered, and then dried in vacuo. The residue was purified by silica gel column chromatography (ethyl acetate/*n*-hexane/dichloromethane = 10:1:1) to give the title compound (650 mg, 79% yield) as yellow solid. Mp: 340.2 – 341.2 °C; ^{1}H NMR (500 MHz, $C_2D_2Cl_4$, 413 K) δ 7.77 – 7.69 (m, 4H), 7.67 (d, J = 7.1 Hz, 2H), 7.47 – 7.34 (m, 4H), 7.34 – 7.23 (m, 5H), 7.23 – 7.11 (m, 9H), 7.11 – 6.98 (m, 1H), 1.28 (s, 18H); ^{13}C NMR (126 MHz, $C_2D_2Cl_4$, 413 K) δ 192.69, 150.21, 144.67, 144.60, 142.01, 141.77, 140.44, 140.11, 138.00, 137.05, 137.00, 136.33, 134.77, 134.66, 133.94, 133.51, 132.18, 132.01, 128.98, 128.41, 128.12, 128.01, 124.44, 123.69, 122.69, 73.56, 34.04, 30.86; HRMS (APCI) [M$^+$] Calculated for $C_{52}H_{42}O_2$: 698.3179; Found 698.3189.

4,4'-(4,4''-di-*tert*-butyl-[1,1':4',1''-terphenyl]-2',5'-diyl)bis(9-mesityl-9*H*-fluorene) (**13**)

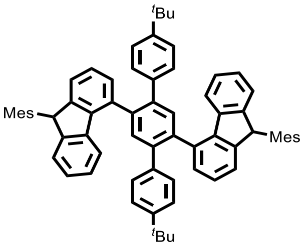

n-BuLi (5.5 mL, 8.8 mmol, 1.6 M in *n*-hexane) was added dropwise to a solution of 2-bromomesitylene (1.85 g, 9.29 mmol) in anhydrous tetrahydrofuran (30 mL) at –78 °C under argon atmosphere, over a period of 30 min. After addition, the mixture was stirred at this temperature for 2 h. Thus prepared solution of mesityllithium was added slowly to a solution of 4,4'-(4,4''-di-*tert*-butyl-[1,1':4',1''-terphenyl]-2',5'-diyl)bis(9*H*-fluoren-9-one) (**12**) (650 mg, 0.929 mmol) in tetrahydrofuran (30 mL) at –78 °C. After the addition, the mixture was stirred at room temperature overnight. The reaction was quenched by addition of saturated aqueous NH_4Cl solution, extracted with ethyl acetate for three times, dried over $MgSO_4$ and evaporated. The residue was dissolved in dry dichloromethane (120 mL), to which $BF_3{\cdot}OEt_2$ (1.32 g, 9.30 mmol) and Et_3SiH (1.08 g, 9.29 mmol) were added. The resulting mixture was stirred at room temperature for 12 h, and the reaction was quenched with saturated aqueous NH_4Cl solution. The organic layer was separated and the aqueous phase

was extracted with dichloromethane (100 mL) for three times. The organic phases were combined, washed with brine, dried over $MgSO_4$ and evaporated. The residue was purified by silica gel column chromatography (*n*-hexane:ethyl acetate = 10:1) and recrystallized from dichloromethane/methanol to give the title compound (560 mg, 66% yield) as white solid. Mp: 297.4 – 298.2 °C; ^{1}H NMR (500 MHz, $C_2D_2Cl_4$, 413 K) δ 7.80 – 7.68 (m, 2H), 7.58 (t, J = 8.3 Hz, 1H), 7.42 – 7.33 (m, 2H), 7.33 – 7.27 (m, 4H), 7.27 – 7.23 (m, 6H), 7.22 – 7.15 (m, 5H), 7.13 – 7.02 (m, 6H), 6.69 (d, J = 10.7 Hz, 2H), 5.65 – 5.42 (m, 2H), 2.87 – 2.62 (m, 6H), 2.36 (d, J = 1.2 Hz, 6H), 1.32 – 1.20 (m, 20H), 1.10 – 1.04 (m, 2H), 1.03 – 0.97 (m, 2H); ^{13}C NMR (126 MHz, $C_2D_2Cl_4$, 413 K) δ 149.27, 147.84, 147.70, 147.64, 141.11, 140.24, 140.12, 140.06, 139.88, 139.11, 139.03, 138.88, 138.77, 138.58, 137.79, 137.71, 137.50, 137.44, 137.35, 137.24, 136.99, 136.82, 135.61, 134.00, 133.93, 132.42, 132.32, 132.18, 132.04, 130.34, 129.46, 129.39, 129.13, 129.01, 128.50, 126.51, 126.36, 126.28, 126.04, 124.12, 123.96, 123.82, 123.62, 123.47, 123.45, 122.91, 122.84, 122.77, 122.44, 122.40, 49.63, 33.94, 30.98, 20.95, 20.37, 18.42; HRMS (MALDI-TOF) [M$^+$] Calculated for $C_{70}H_{66}$: 906.5159; Found 906.5130.

2,10-di-*tert*-butyl-6,14-dimesityl-6,14-dihydrodibenzo[ef,no]difluoreno[3,4,5,6-hijkl:3',4',5',6'-qrabc]coronene (**14**)

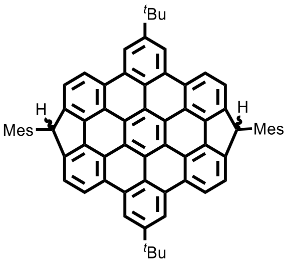

4,4'-(4,4''-Di-*tert*-butyl-[1,1':4',1''-terphenyl]-2',5'-diyl)bis(9-mesityl-9*H*-fluorene) (**13**) (20 mg, 0.022 mmol) was dissolved in unstabilised dichloromethane (commercially available) (12 mL) and degassed by bubbling with argon flow, which was saturated with dichloromethane vapor, for 10 min. $FeCl_3$ (95 mg, 0.59 mmol) dissolved in nitromethane (1 mL) was added to the above-prepared solution and the resulting mixture was stirred at room temperature for 30 min. Methanol (80 mL) was then added. The precipitate was collected by filtration and washed with methanol and *n*-hexane to give the title compound (17 mg, 86% yield) as light yellow powder. ^{1}H NMR (300 MHz, THF-d_8:CS_2 = 1:1, 298 K) δ 9.24 (s, 4H), 9.03 (d, J = 8.0 Hz, 4H), 8.93 (s, 4H), 8.71 (d, J = 7.3 Hz, 4H), 7.99 (d, J = 7.8 Hz, 4H), 7.85 (d, J = 7.8 Hz, 4H), 7.16 (d, J = 6.5 Hz, 4H), 6.64 (s, 2H), 6.50 (s, 2H), 6.36 (s, 4H), 3.20 (s, 6H), 3.00 (s, 6H), 2.33 (s, 6H), 2.23 (s, 6H), 1.89 (s, 18H), 1.84 (s, 18H), 1.04 (s, 6H), 0.61 (s, 6H). The powder was further separated by silica gel column chromatography (*n*-hexane:dichloromethane = 3:1) to give an isomer of the title compound (**14-I**): Mp: >400, decomposition; ^{1}H NMR (300 MHz, THF-d_8:CS_2 = 1:1, 298 K) δ 9.25 (s, 4H), 9.04 (d, J = 8.4 Hz, 4H), 8.00 (d, J = 7.9 Hz, 4H), 7.15 (s, 2H), 6.64 (s, 2H), 6.37 (s, 2H), 2.99 (s, 7H), 1.87 (s, 6H), 1.84 (s, 18H), 1.04 (s, 6H); HRMS (MALDI-TOF) [M$^+$] Calculated for $C_{70}H_{54}$: 894.4220; Found 894.4205 and another isomer **14-II**: Mp: >400, decomposition; ^{1}H NMR (300 MHz, THF-d_8:CS_2 = 1:1, 298 K) δ 8.81 (s, 4H), 8.58 (d, J = 7.8 Hz, 4H), 7.79 (d, J = 7.6 Hz, 4H), 7.20 (s, 2H), 6.47 (s, 2H), 6.32 (s, 2H), 3.30 (s, 6H), 2.29 (s, 7H), 1.92 (s, 19H), 0.48 (s, 6H); ^{13}C NMR (75 MHz, THF-d_8:CS_2 = 1:1, 298 K) δ 148.91, 144.69, 139.08, 138.28, 136.91, 136.33, 133.76, 131.37, 131.25, 130.09, 129.10, 125.60, 122.36, 121.83, 121.78, 120.26, 119.94, 119.36, 55.45, 36.55, 32.95, 23.27, 21.45, 18.88; HRMS (MALDI-TOF) [M$^+$] Calculated for $C_{70}H_{54}$: 894.4220; Found 894.4193.

In-situ generation of *p*PHBC **16**

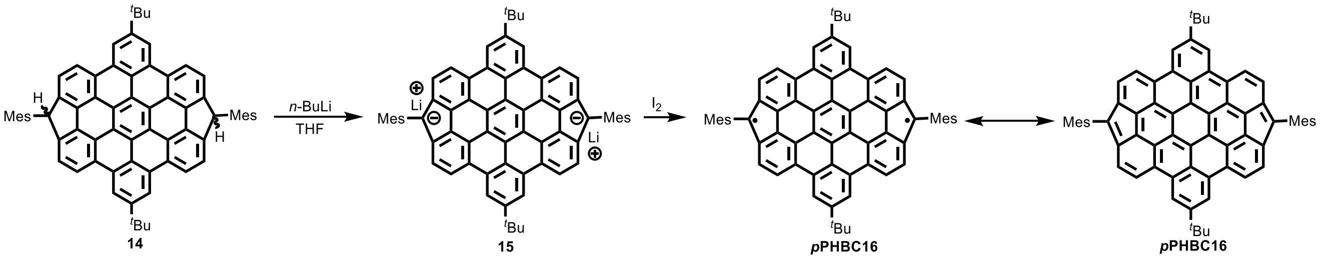

2,10-Di-*tert*-butyl-6,14-dimesityl-6,14-dihydrodibenzo[*ef*,*no*]difluoreno[3,4,5,6-*hijkl*:3',4',5',6'-*qrabc*]coronene (**13**) (4.0 mg, 0.0045 mmol) was placed in an NMR tube under argon protection and dry tetrahydrofuran-d_8 (0.6 mL) was added. At room temperature, *n*-BuLi (6.0 µL, 0.0096 mmol, 1.6 M in *n*-hexane) was added, generating a dark green solution of **15**. UV/Vis (tetrahydrofuran): λ_{max} (ε) = 692 nm (8286 M^{-1}cm^{-1}), 646 nm (7398 M^{-1}cm^{-1}), 535 nm (5808 M^{-1}cm^{-1}), 464 nm (20626 M^{-1}cm^{-1}), 444 nm (20070 M^{-1}cm^{-1}), 352 nm (57704 M^{-1}cm^{-1}); ^{1}H NMR (300 MHz, THF-d_8, 298 K) δ 9.36 (s, 4H), 9.14 (d, J = 8.3 Hz, 4H), 8.16 (d, J = 8.4 Hz, 4H), 7.09 (s, 4H), 2.47 (s, 18H), 1.94 (s, 18H); ^{13}C NMR (75 MHz, THF-d_8, 298 K) δ 138.80, 136.39, 134.22, 133.54, 132.68, 132.33, 132.11, 131.55, 128.75, 120.60, 120.41, 116.48, 112.82, 42.48, 37.30, 35.65, 32.72, 30.12. To the above-prepared mixture containing **15** was added a solution of I_2 (2.5 mg, 0.009 mmol) in tetrahydrofuran-d_8 (0.1 mL) to generate *p*PHBC **16** as indicated by the change of the color of the solution to brown. UV/Vis (tetrahydrofuran): λ_{max} (ε) = 770 nm (3960 M^{-1}cm^{-1}), 698 nm (2202 M^{-1}cm^{-1}), 565 nm (5598 M^{-1}cm^{-1}), 463 nm (9788 M^{-1}cm^{-1}), 419 nm (21576 M^{-1}cm^{-1}).

3. Supplementary MS Spectra

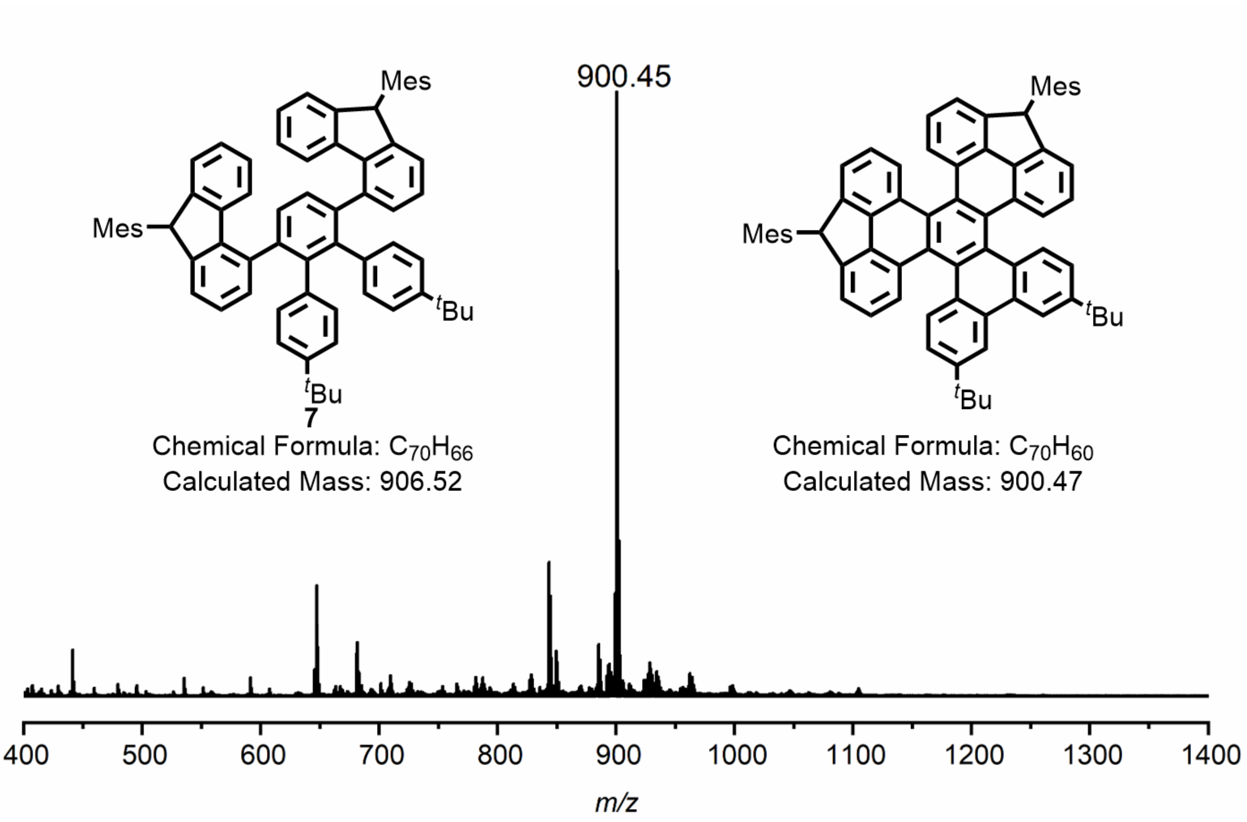

Figure S1. MALDI-TOF MS spectrum after oxidation of **7** with $FeCl_3$ (26 eq.) in dichloromethane at room temperature.

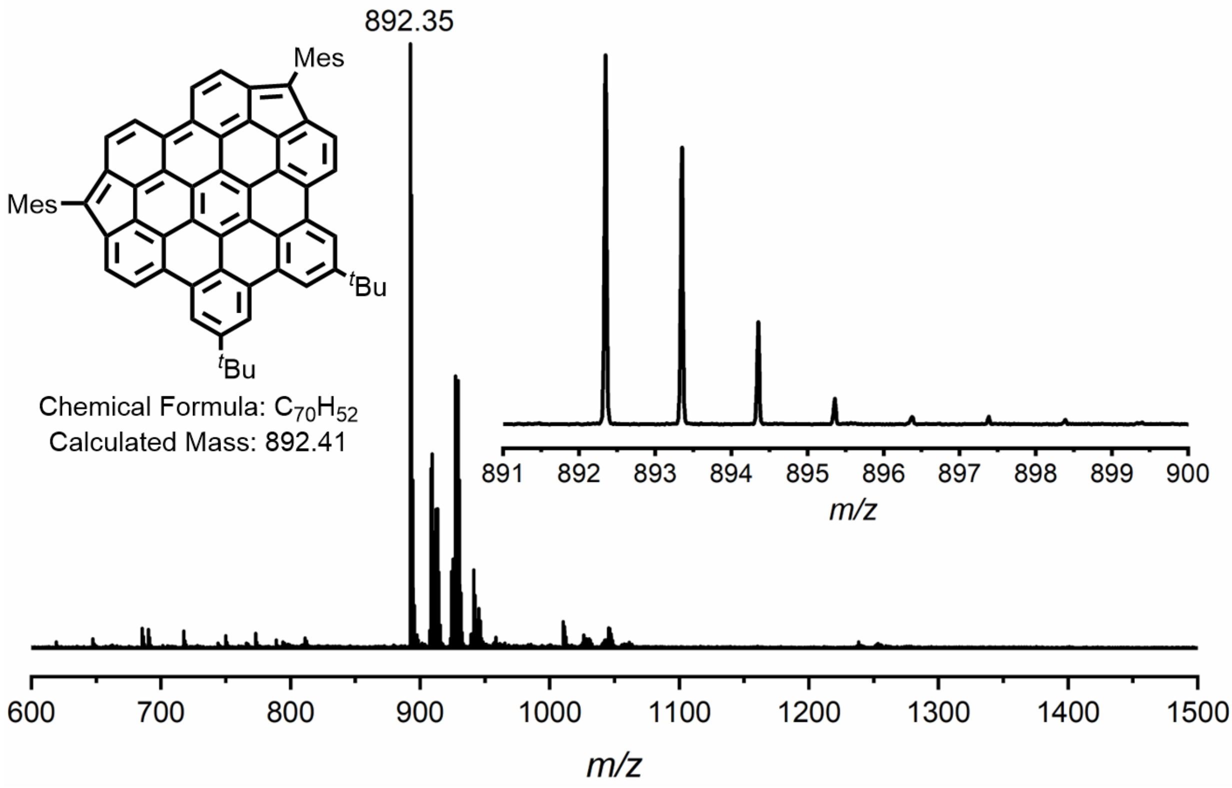

Figure S2. MALDI-TOF MS spectrum of reaction mixture after oxidation of **7** with DDQ/MSA in dichloromethane followed by addition of TFMSA; inset shows experimental isotopic distribution pattern of product.

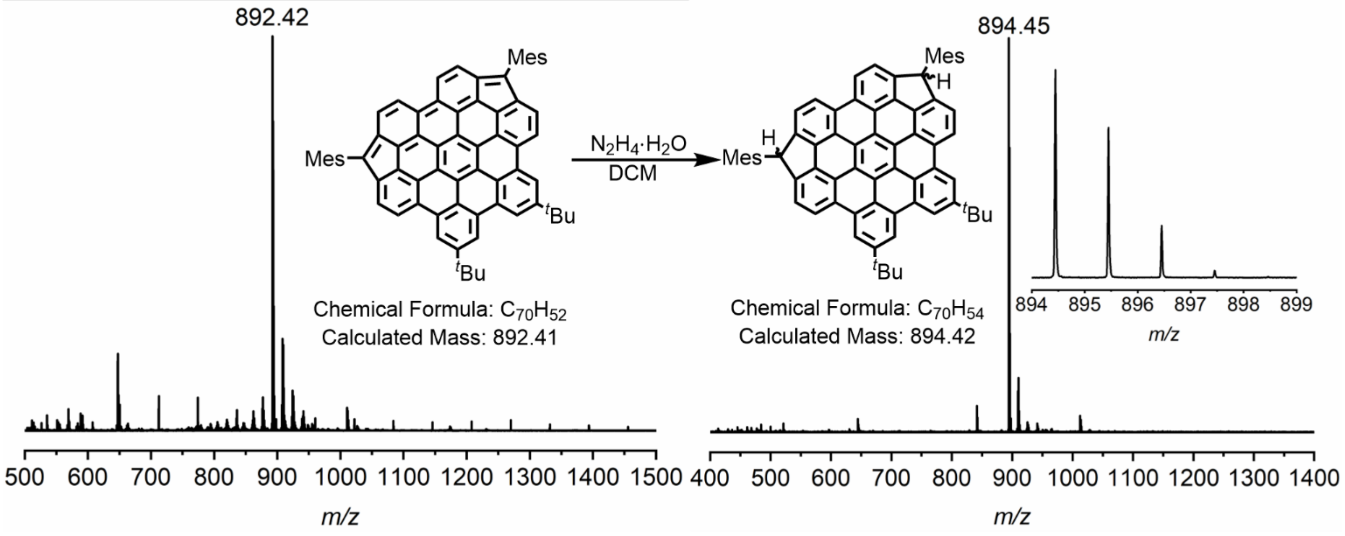

Figure S3. MALDI-TOF MS spectrum of *m*PHBC **8** before (left) and after (right) reducing with excess hydrazine solution in tetrahydrofuran; inset shows experimental isotopic distribution pattern of dehydrogenated *m*PHBC.

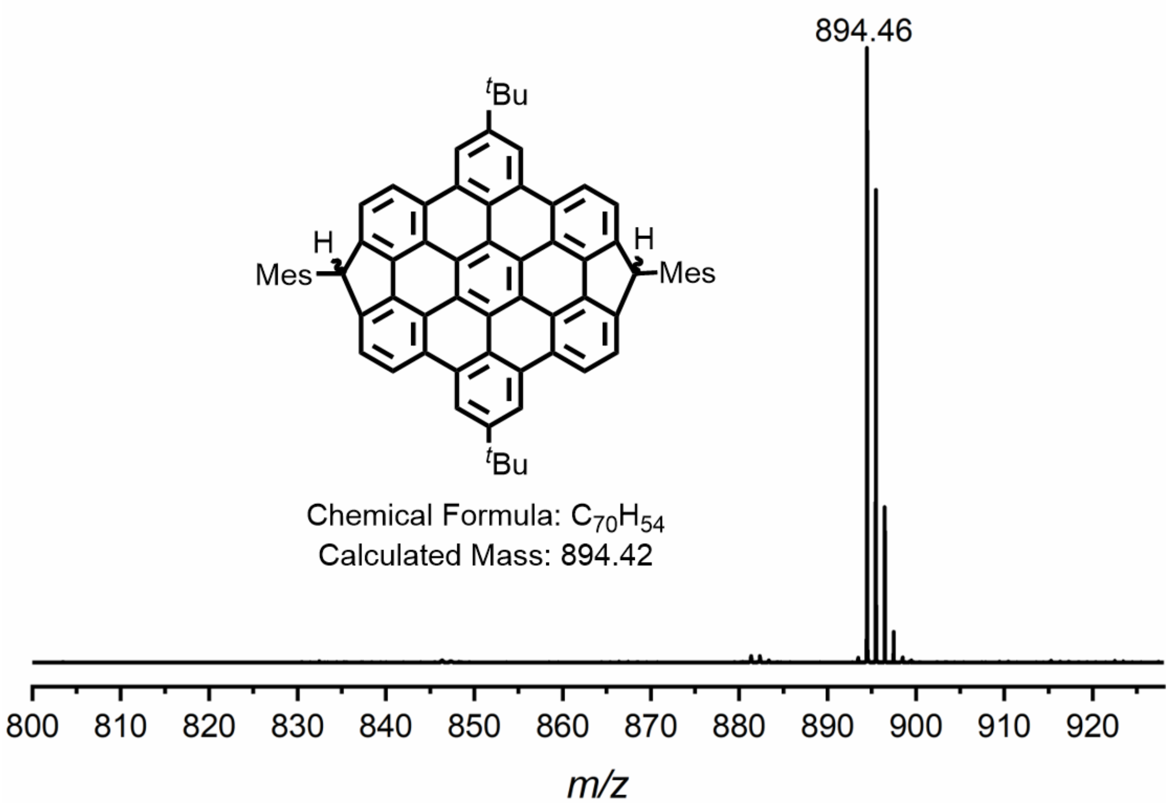

Figure S4. MALDI-TOF MS spectrum of 2,10-di-*tert*-butyl-6,14-dimesityl-6,14-dihydrodibenzo[*ef*,*no*]difluoreno[3,4,5,6-hijkl:3',4',5',6'-qrabc]coronene (**14**).

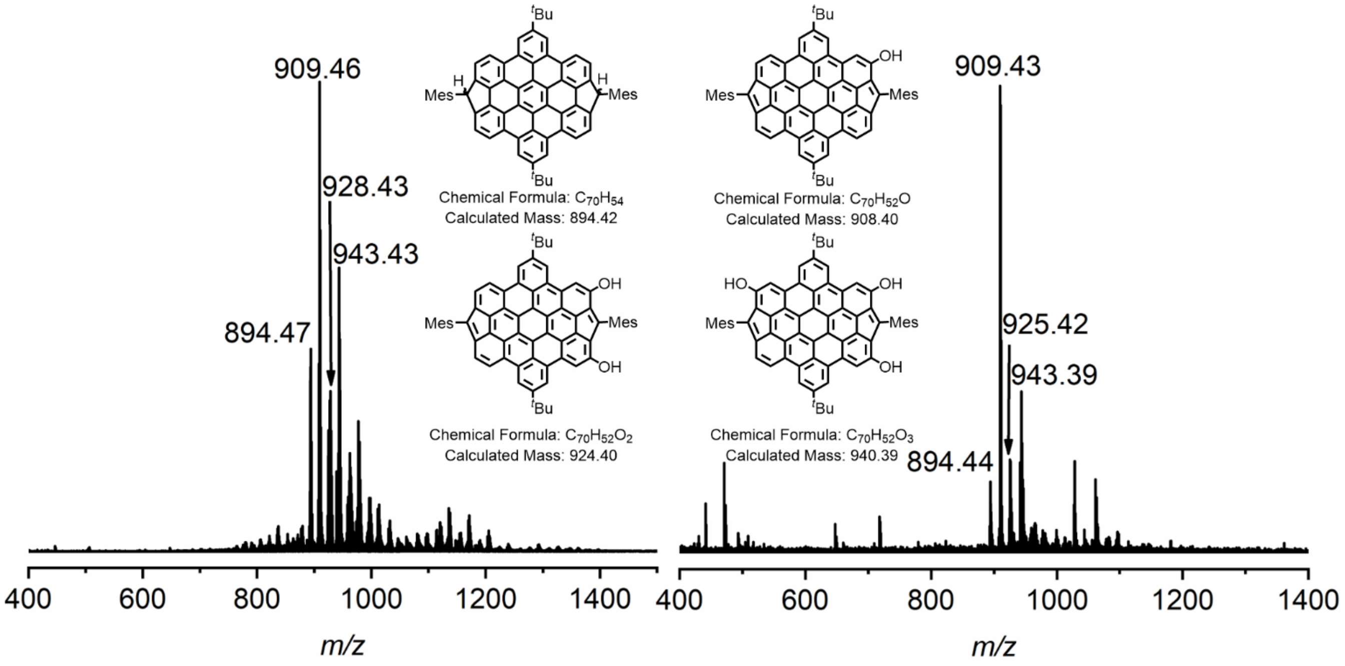

Figure S5. MALDI-TOF MS spectrum after the oxidation of **14** with DDQ (left) and DMF/^{t}BuOK (right).

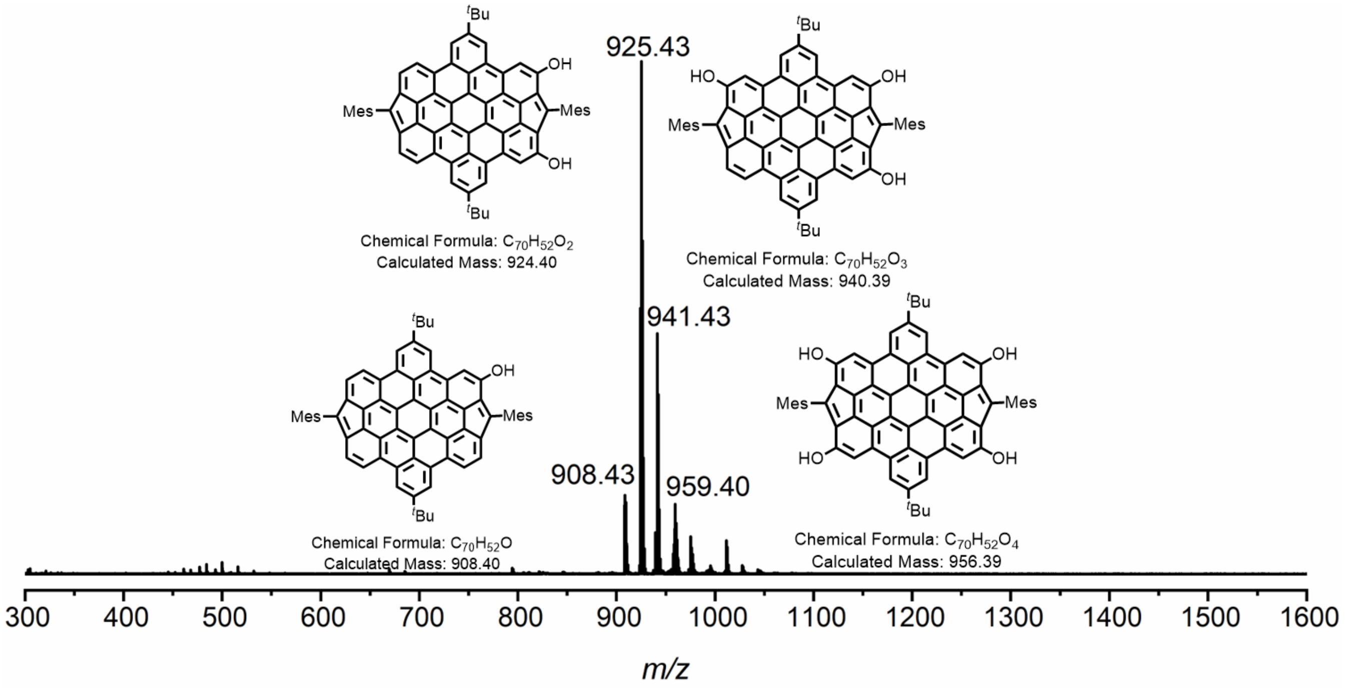

Figure S6. MALDI-TOF MS spectrum measured after *in situ* deprotonation and oxidation of **14**, showing formation of oxidized species of *p*PHBC **16**.

4. Supplementary UV-vis Absorption Spectra

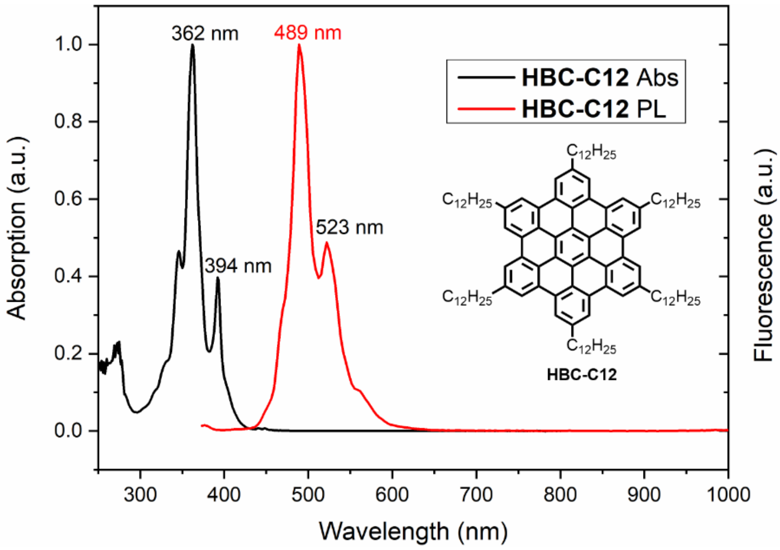

Figure S7. UV-vis absorption and fluorescence spectra of **HBC-C12** in toluene at a concentration of 10^{-5} M.

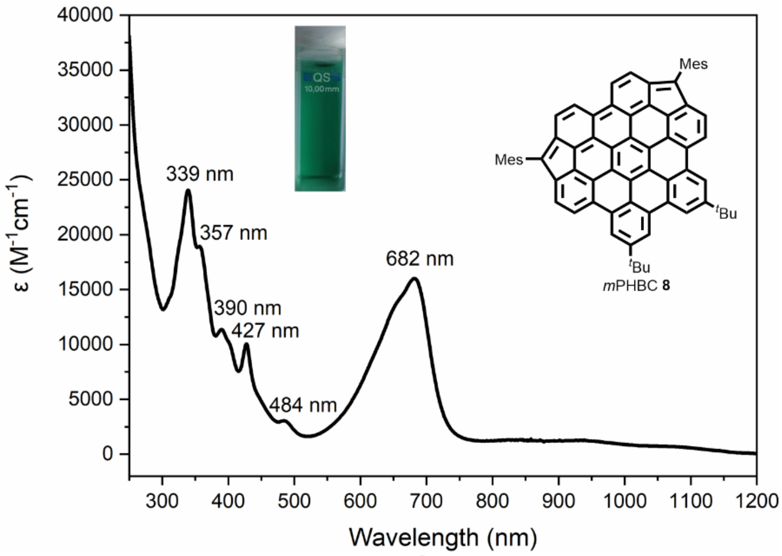

Figure S8. UV-vis absorption spectra of *m*PHBC **8** in dichlormethane (c = 3.64 × 10^{-5} M); inset: photograph of the solution.

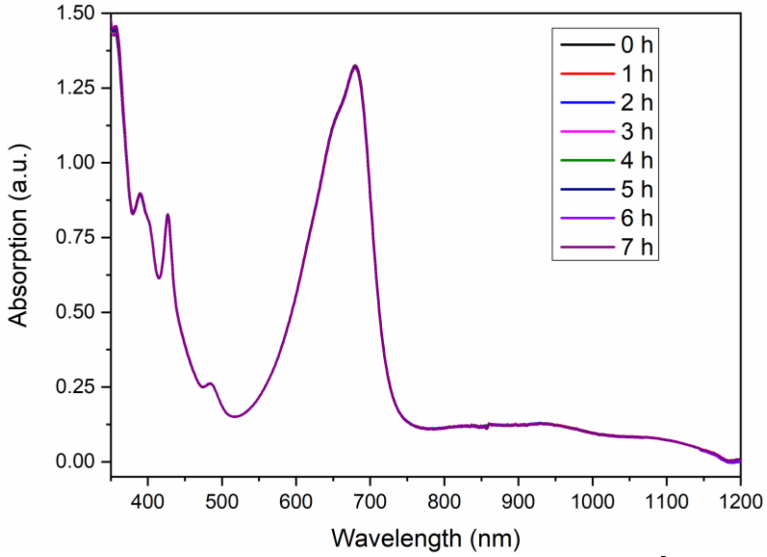

Figure S9. Time-dependent UV-vis absorption spectra of a solution of *m*PHBC **8** in dichloromethane (c = 2.97 × 10^{-5} M) under nitrogen and in dark.

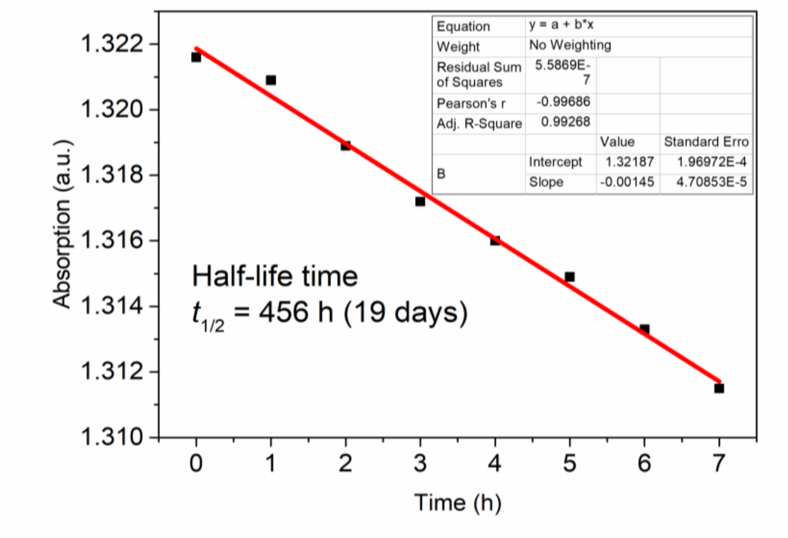

Figure S10. Changes of the absorption intensity at 682 nm with time. The half-life time was estimated according to the zero-order kinetics to be 456 h (19 days).

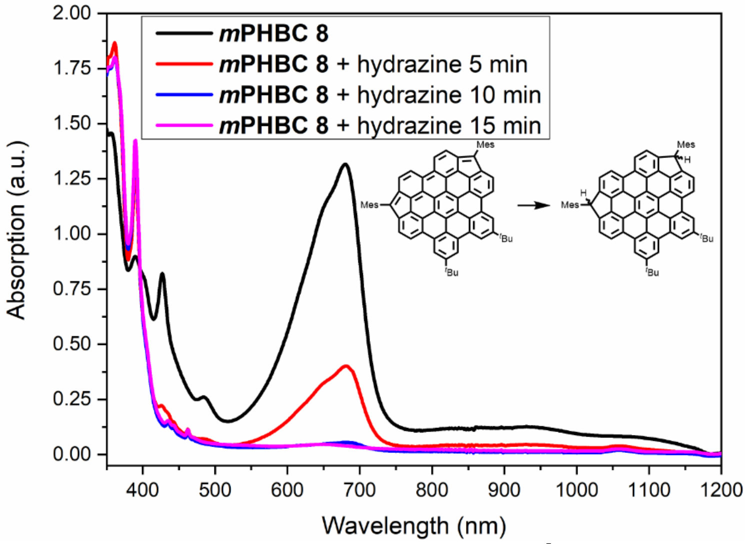

Figure S11. UV-vis absorption spectral changes of *m*PHBC **8** solution in dichloromethane (c = 2.97 × 10^{-5} M) (5, 10, and 15 minutes) after the addition of hydrazine (tetrahydrofuran solution).

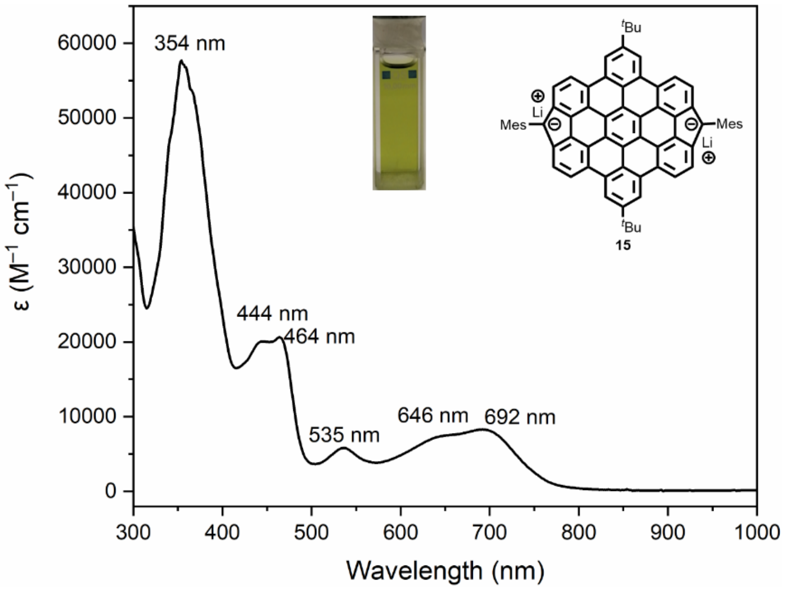

Figure S12. UV-vis absorption spectrum of dianion **15** in tetrahydrofuran (c = 5.0 × 10^{-5} M); inset: photograph of the solution.

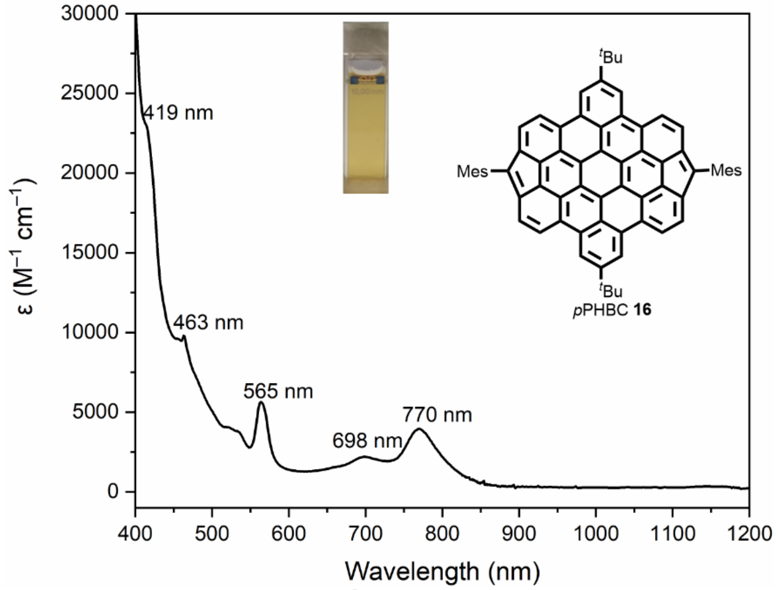

Figure S13. UV-vis absorption of *p*PHBC **16** in tetrahydrofuran (c = 5.0 × 10^{-5} M); inset: photograph of the solution.

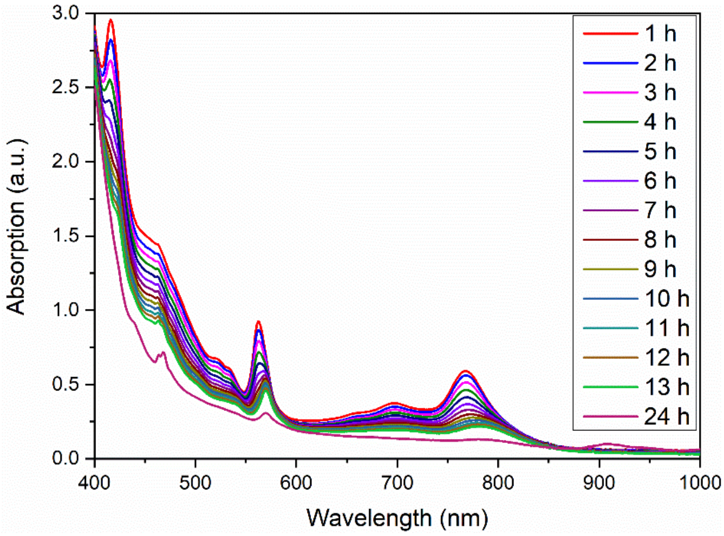

Figure S14. Time-dependent UV-vis absorption of *in situ* generated *p*PHBC **16**.

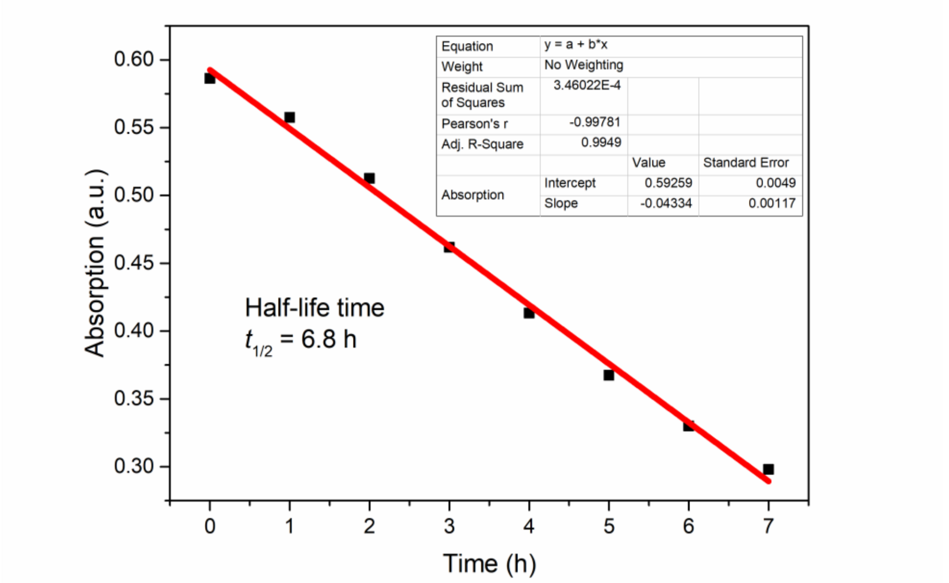

Figure S15. Uv-vis absorption intensity changes of *in situ* generated *p*PHBC **16** in tetrahydrofuran (c = 5.0 × 10^{-5} M) at 770 nm with time. The half-life time was estimated according to the zero-order kinetics to be 6.8 h.

5. Theoretical Calculations

All calculations were performed with Gaussian 09, Revision D.01 using the 6-31G(d,p) basis set.[4] Geometrical optimizations were performed using the broken-symmetry unrestricted B3LYP method for singlet states with the keyword guess=mix and the unrestricted B3LYP method for triplet states. Time-dependent density functional theory (TD-DFT) calculations were performed on the broken-symmetry unrestricted B3LYP method for singlet states. Diradical characters (y_0) were calculated using the spin unrestricted UHF method based on the occupation numbers of natural orbitals (UNO) (equations 1 and 2, where T is the orbital overlap between the highest occupied natural orbital (HONO) and the lowest unoccupied natural orbital (LUNO) and n is the occupation numbers of UNO).[5] Nucleus-independent chemical shift (NICS) analysis was performed by the GIAO-UB3LYP/6-31G(d,p) method.[6]

$$y_0 = 1 - \frac{2T}{1+T^2} \quad (1)$$
$$T = \frac{n_{HONO} - n_{LUNO}}{2} \quad (2)$$

Table S1. Calculated (UB3LYP/6-31G(d,p)) relative energy, singlet-triplet gap ($\Delta E_{S\text{-}T}$) of the closed-shell singlet (CS), open-shell singlet (OS), and open-shell triplet (OT) states of *m*PHBC **8** and *p*PHBC **16**.

	CS (kcal/mol)	OS (kcal/mol)	OT (kcal/mol)	$\Delta E_{S\text{-}T}$ (kcal/mol)
*m*PHBC **8**	0	–0.00063	1.80	–1.80
*p*PHBC **16***	0	–3.02	–2.84	–0.18

*Energy were calculated at UBLYP/6-31G(d,p) level of theory.

Table S2. Selected TD-DFT (UB3LYP/6-31G(d,p)) calculated energies, oscillator strength and compositions of major electronic transitions of singlet state of *m*PHBC **8** (A: α spin; B: β spin).

Wavelength (nm)	Osc. Strength (f)	Major contributions
1728.97	0.0019	HOMO(A)->LUMO(A) (50%), HOMO(B)->LUMO(B) (50%)
1011.54	0.0761	H-1(A)->LUMO(A) (10%), HOMO(A)->LUMO(A) (35%), HOMO(B)->LUMO(B) (35%) H-2(A)->LUMO(A) (6%), H-2(B)->LUMO(B) (5%), H-1(B)->LUMO(B) (7%)
958.59	0.002	H-1(A)->LUMO(A) (43%), H-1(B)->LUMO(B) (44%) H-2(A)->LUMO(A) (4%), H-2(B)->LUMO(B) (4%)
820.87	0.0038	H-2(A)->LUMO(A) (35%), H-1(A)->LUMO(A) (10%), H-2(B)->LUMO(B) (35%), H-1(B)->LUMO(B) (11%)
743.48	0.0859	H-3(A)->LUMO(A) (34%), H-3(B)->LUMO(B) (37%) H-1(A)->LUMO(A) (8%), HOMO(A)->LUMO(A) (5%), H-1(B)->LUMO(B) (7%), HOMO(B)->LUMO(B) (5%)
719.83	0.0003	H-2(A)->LUMO(A) (46%), H-2(B)->LUMO(B) (42%) H-1(A)->LUMO(A) (4%), H-1(B)->LUMO(B) (5%)
659.84	0.3905	H-1(A)->LUMO(A) (21%), H-3(B)->LUMO(B) (12%), H-2(B)->LUMO(B) (10%), H-1(B)->LUMO(B) (23%) H-3(A)->LUMO(A) (9%), H-2(A)->LUMO(A) (5%), HOMO(A)->LUMO(A) (8%), HOMO(B)->LUMO(B) (8%)
619.70	0.104	H-3(A)->LUMO(A) (50%), H-3(B)->LUMO(B) (42%) HOMO(A)->L+1(A) (2%), HOMO(B)->L+1(B) (2%)

Table S3. Selected TD-DFT (B3LYP/6-31G(d,p)) calculated energies, oscillator strength and compositions of major electronic transitions of *p*PHBC dianion **15**.

Wavelength (nm)	Osc. Strength (f)	Major contributions
587.99	0.0000	H-1->LUMO (99%)
565.23	0.2107	HOMO->LUMO (91%) H-2->L+1 (5%)
542.22	0.2095	HOMO->L+1 (91%) H-2->LUMO (7%)
505.48	0.0	H-1->L+1 (98%)

445.44	0.0	HOMO->L+2 (92%) H-3->LUMO (2%), H-1->L+3 (3%)
440.80	0.0317	H-2->LUMO (59%), H-1->L+2 (31%) HOMO->L+1 (6%)
435.06	1.3013	H-2->LUMO (20%), H-1->L+2 (51%), HOMO->L+3 (22%)

Table S4. Selected TD-DFT (UB3LYP/6-31G(d,p)) calculated energies, oscillator strength and compositions of major electronic transitions of singlet state of *p*PHBC **16** (A: α spin; B: β spin).

Wavelength (nm)	Osc. Strength (f)	Major contributions
1025.43	0.0598	HOMO(A)->LUMO(A) (48%), HOMO(B)->LUMO(B) (47%)
1019.61	0.0	H-2(A)->LUMO(A) (10%), H-1(A)->LUMO(A) (36%), H-2(B)->LUMO(B) (10%), H-1(B)->LUMO(B) (36%) HOMO(A)->LUMO(A) (3%), HOMO(B)->LUMO(B) (3%)
982.75	0.0	HOMO(A)->LUMO(A) (46%), HOMO(B)->LUMO(B) (47%) H-1(A)->LUMO(A) (2%), H-1(B)->LUMO(B) (3%)
982.05	0.0008	H-1(A)->LUMO(A) (41%), H-1(B)->LUMO(B) (40%) H-2(A)->LUMO(A) (8%), H-2(B)->LUMO(B) (8%)
721.34	0.0	H-2(A)->LUMO(A) (35%), H-1(A)->LUMO(A) (11%), H-2(B)->LUMO(B) (35%), H-1(B)->LUMO(B) (11%) HOMO(A)->L+1(A) (2%), HOMO(B)->L+1(B) (2%)
670.66	0.0411	H-2(A)->LUMO(A) (38%), H-2(B)->LUMO(B) (38%) H-1(A)->LUMO(A) (8%), H-1(B)->LUMO(B) (8%)
619.95	0.0785	H-3(A)->LUMO(A) (45%), H-3(B)->LUMO(B) (45%)
617.36	0.0	H-3(A)->LUMO(A) (46%), H-3(B)->LUMO(B) (46%)

6. NMR Spectra

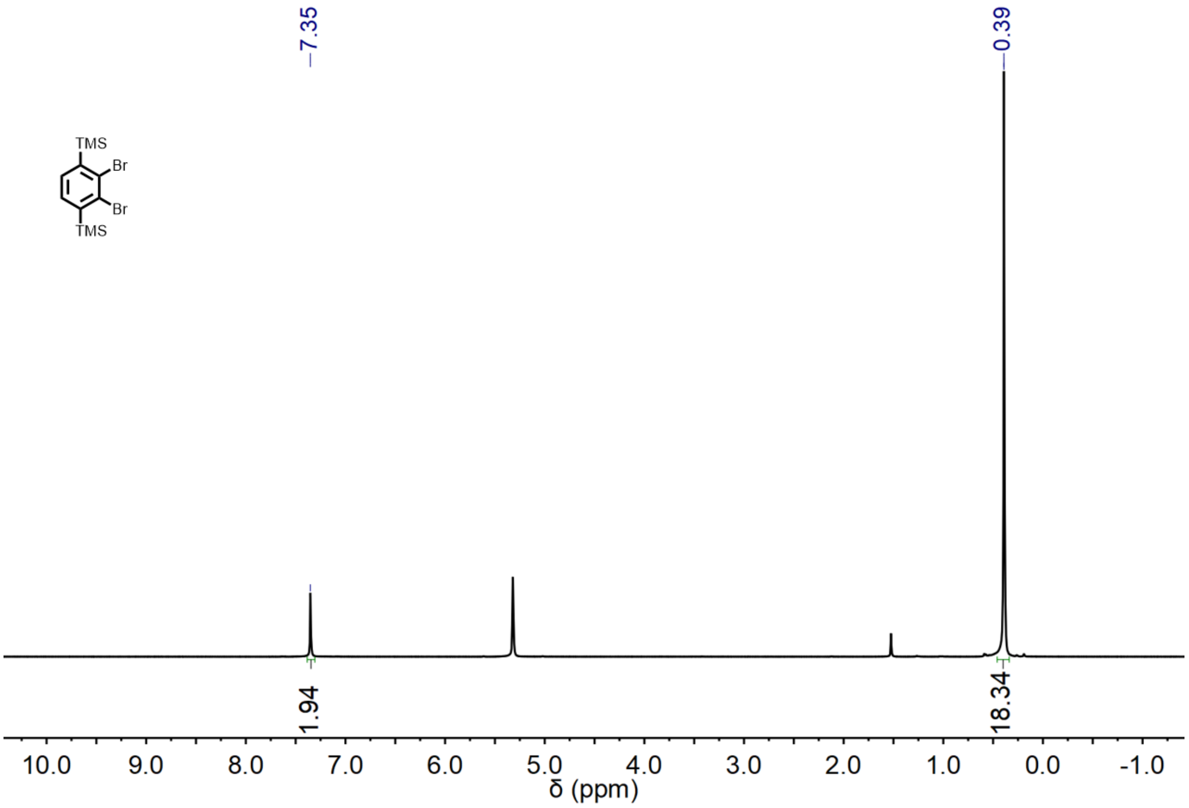

Figure S16. ^{1}H NMR spectrum of (2,3-dibromo-1,4-phenylene)bis(trimethylsilane) (**2**) (300 MHz, CD_2Cl_2, 298 K).

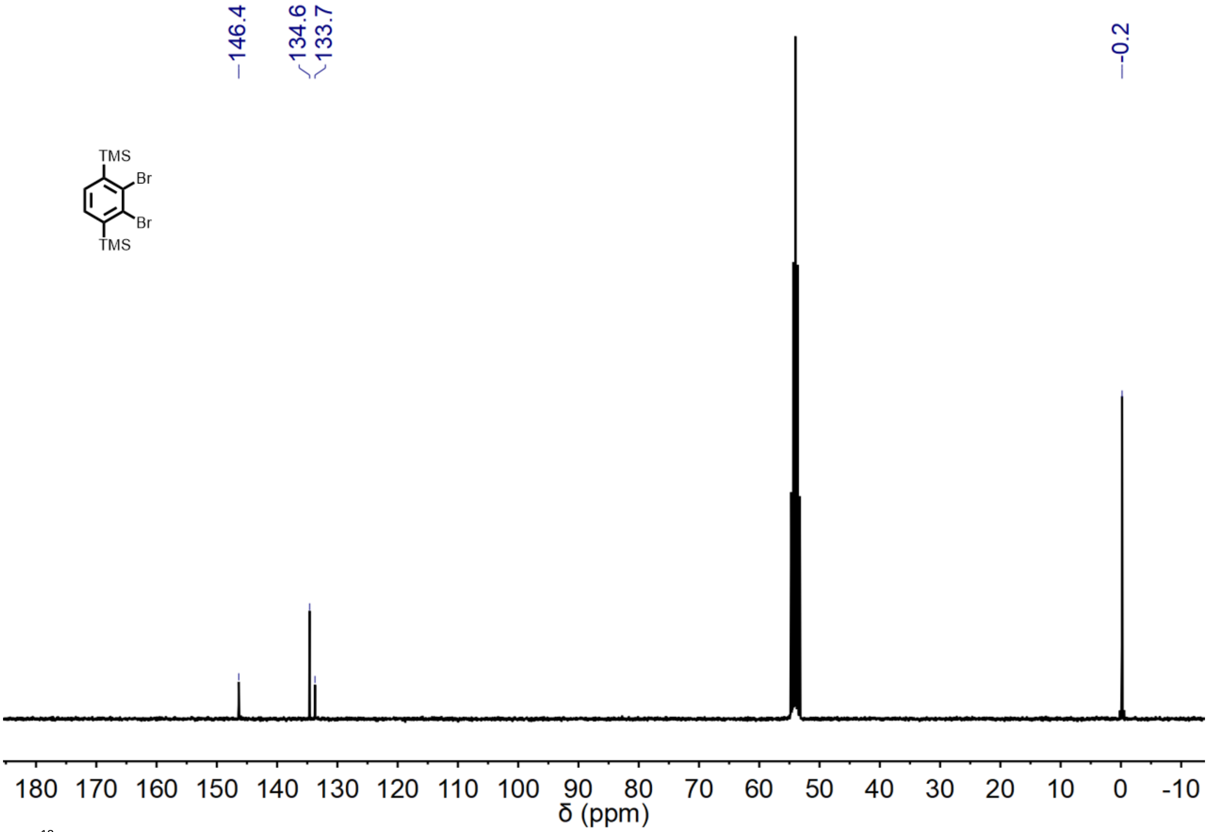

Figure S17. ^{13}C NMR spectrum of (2,3-dibromo-1,4-phenylene)bis(trimethylsilane) (**2**) (75 MHz, CD_2Cl_2, 298 K).

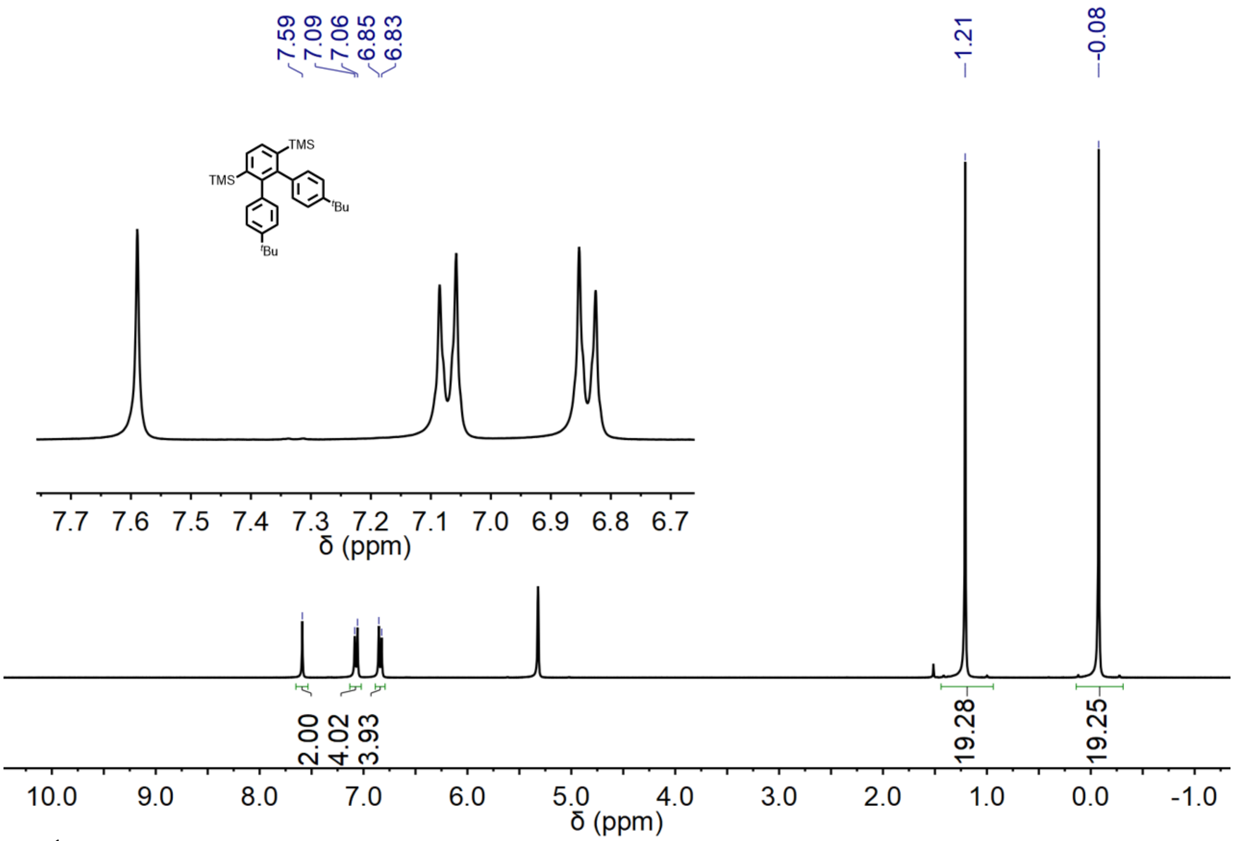

Figure S18. ^{1}H NMR spectrum of (4,4''-di-*tert*-butyl-[1,1':2',1''-terphenyl]-3',6'-diyl)bis(trimethylsilane) (**3**) (300 MHz, CD_2Cl_2, 298 K).

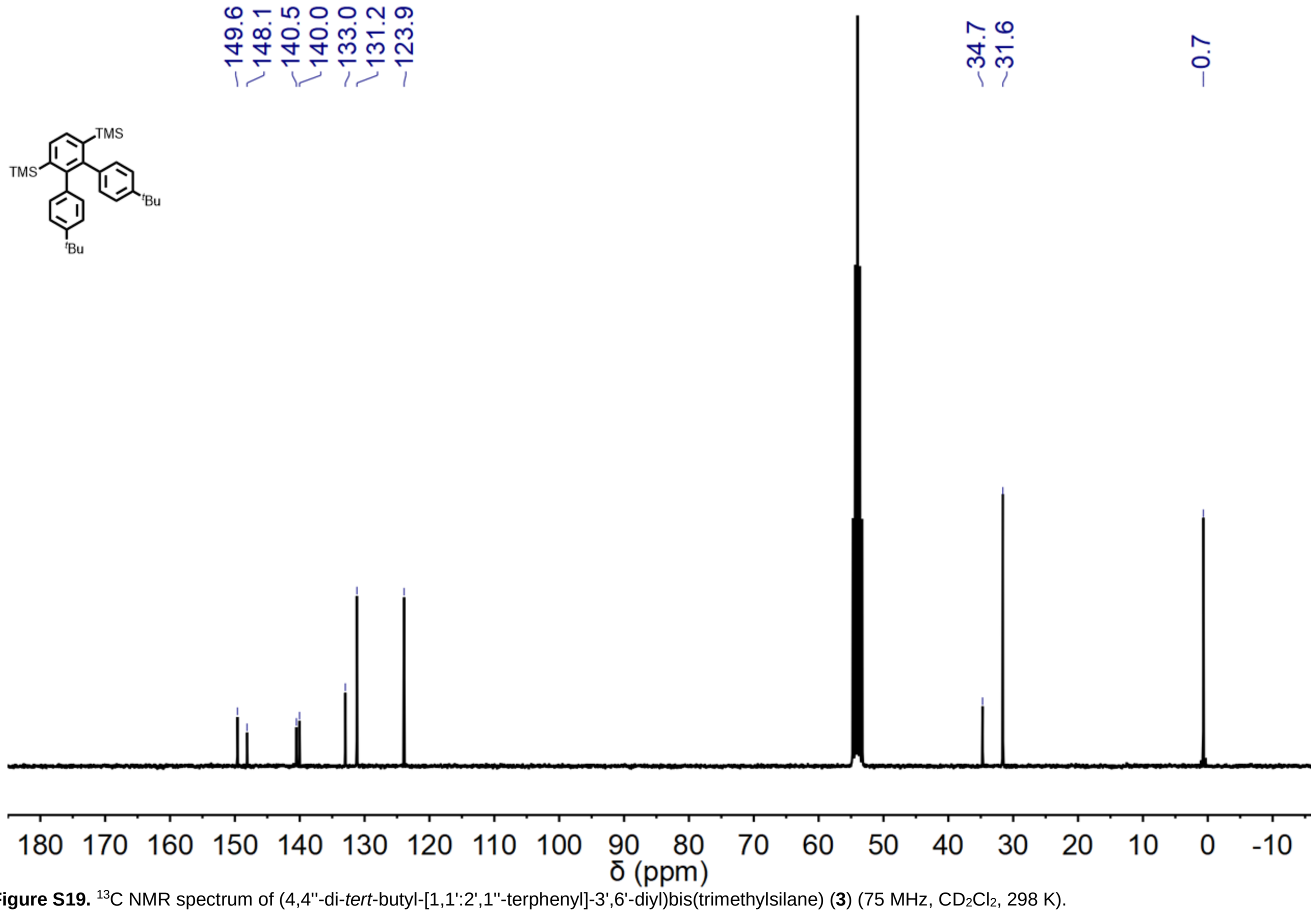

Figure S19. ^{13}C NMR spectrum of (4,4''-di-*tert*-butyl-[1,1':2',1''-terphenyl]-3',6'-diyl)bis(trimethylsilane) (**3**) (75 MHz, CD_2Cl_2, 298 K).

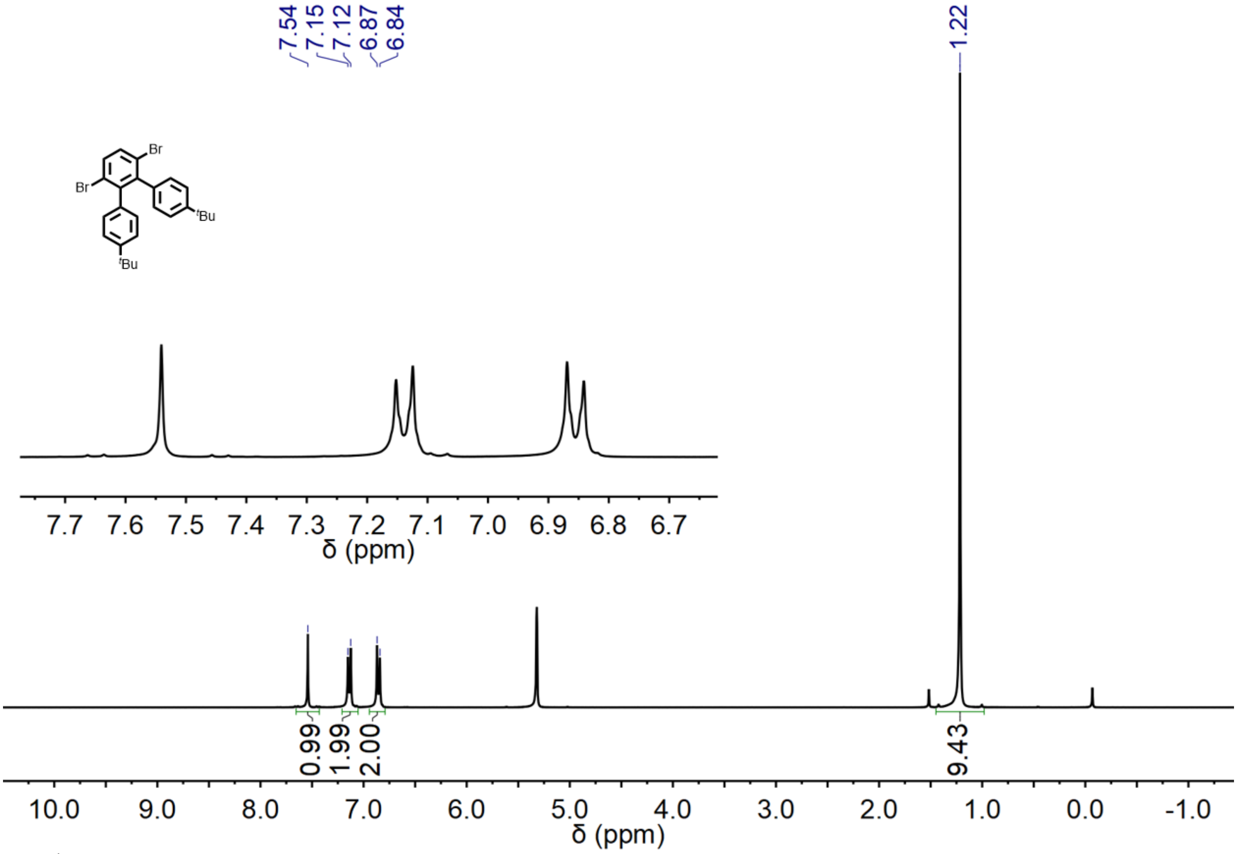

Figure S20. ^{1}H NMR spectrum of 1,4-dibromo-2,3-di-(4-tert-butylphenyl)benzene (**4**) (300 MHz, CD_2Cl_2, 298 K).

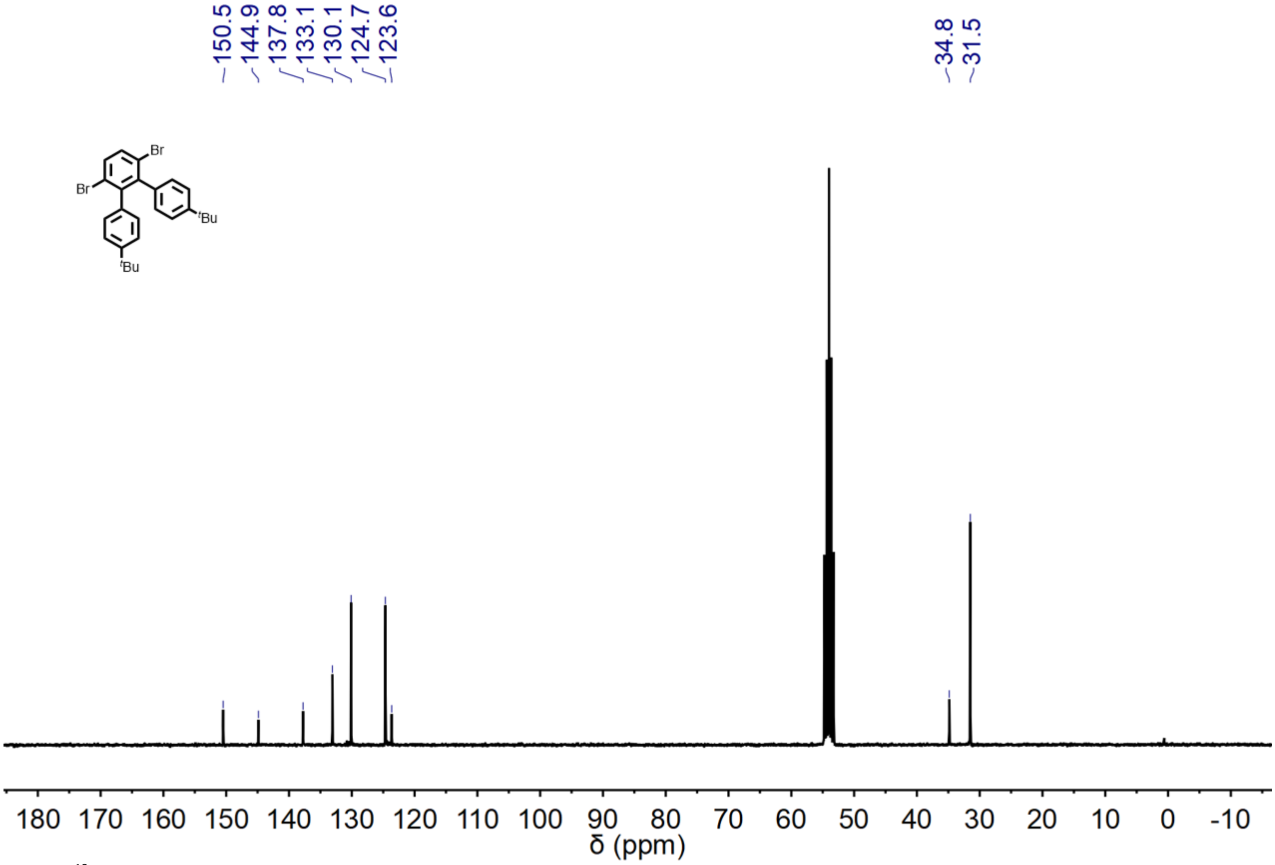

Figure S21. ^{13}C NMR spectrum of 1,4-dibromo-2,3-di-(4-*tert*-butylphenyl)benzene (**4**) (75 MHz, CD_2Cl_2, 298 K).

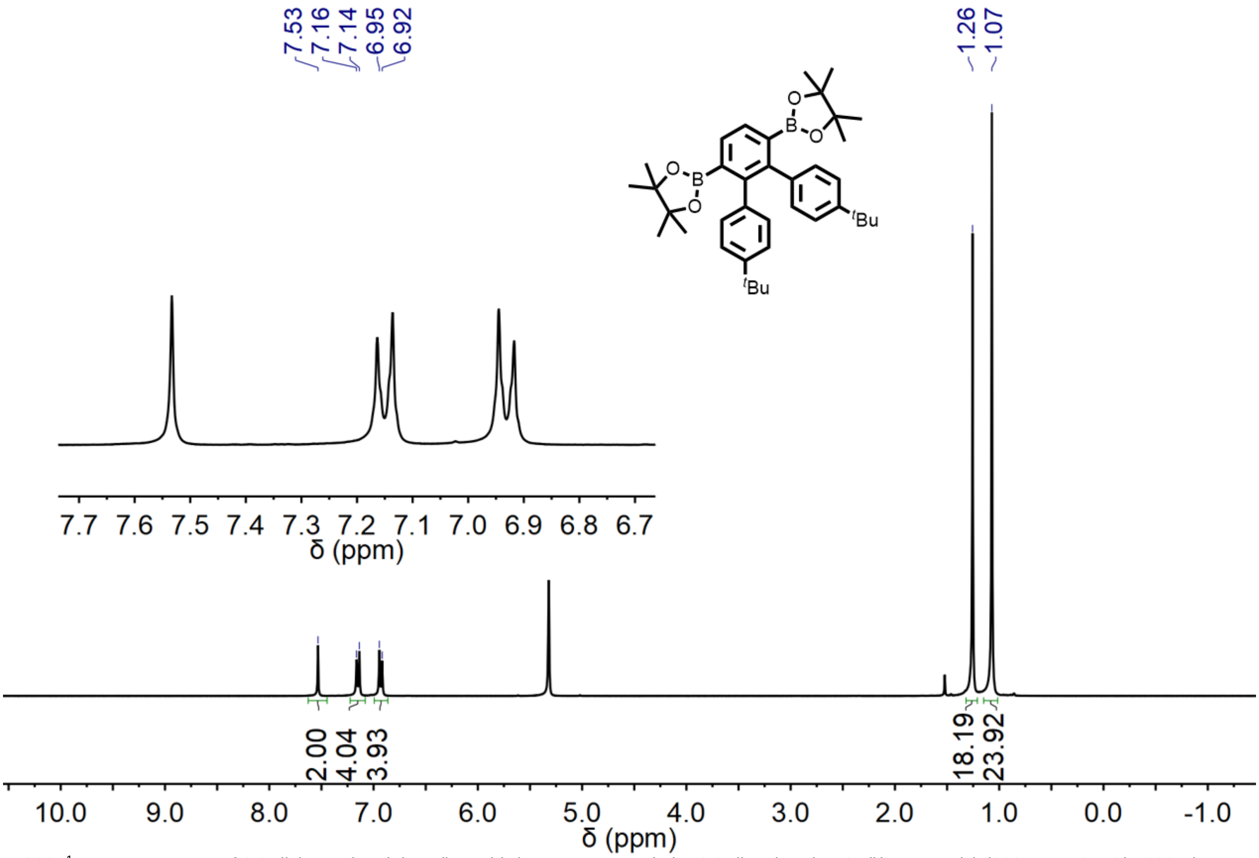

Figure S22. ^{1}H NMR spectrum of 2,3-di-(4-*tert*-butylphenyl)-1,4-bis(4,4,5,5-tetramethyl-1,3,2-dioxaborolan-2-yl)benzene (**5**) (300 MHz, CD_2Cl_2, 298 K).

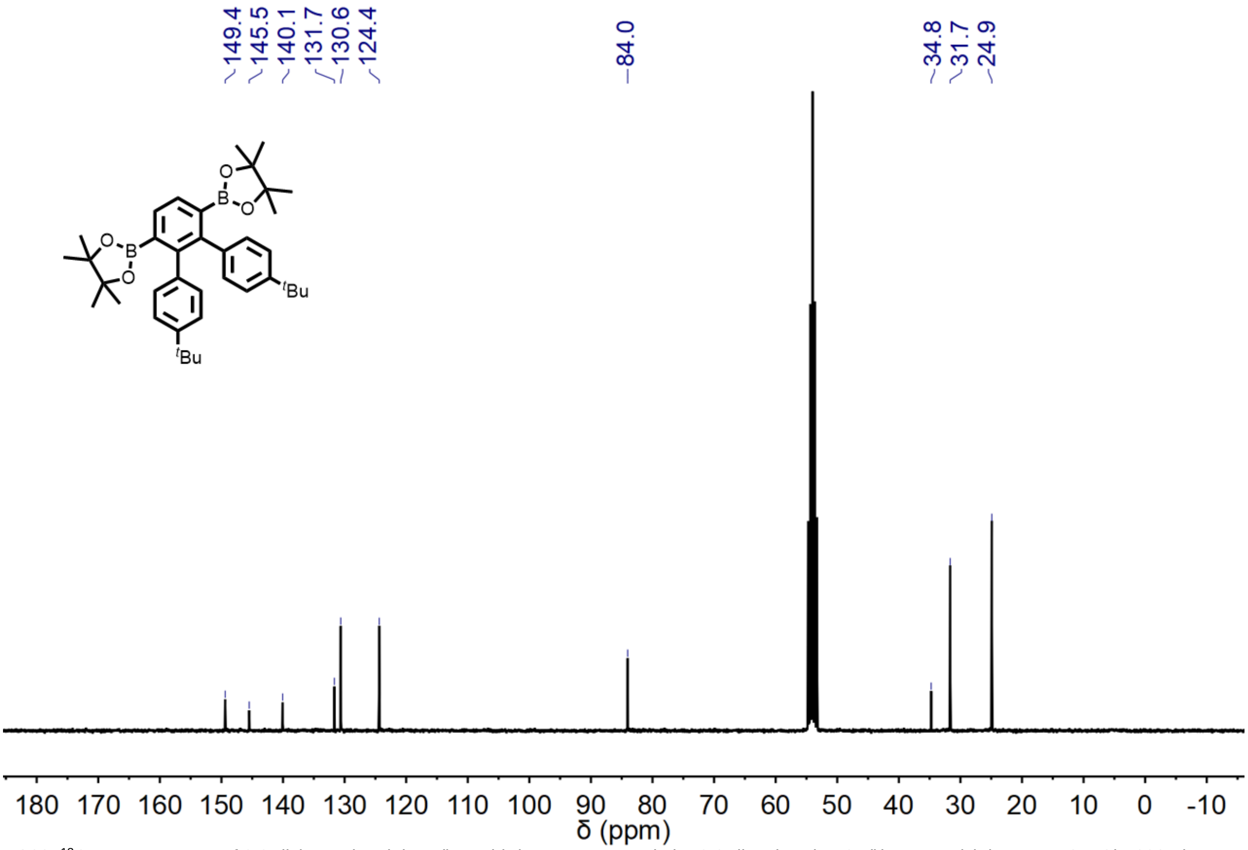

Figure S23. ^{13}C NMR spectrum of 2,3-di-(4-*tert*-butylphenyl)-1,4-bis(4,4,5,5-tetramethyl-1,3,2-dioxaborolan-2-yl)benzene (**5**) (75 MHz, CD_2Cl_2, 298 K).

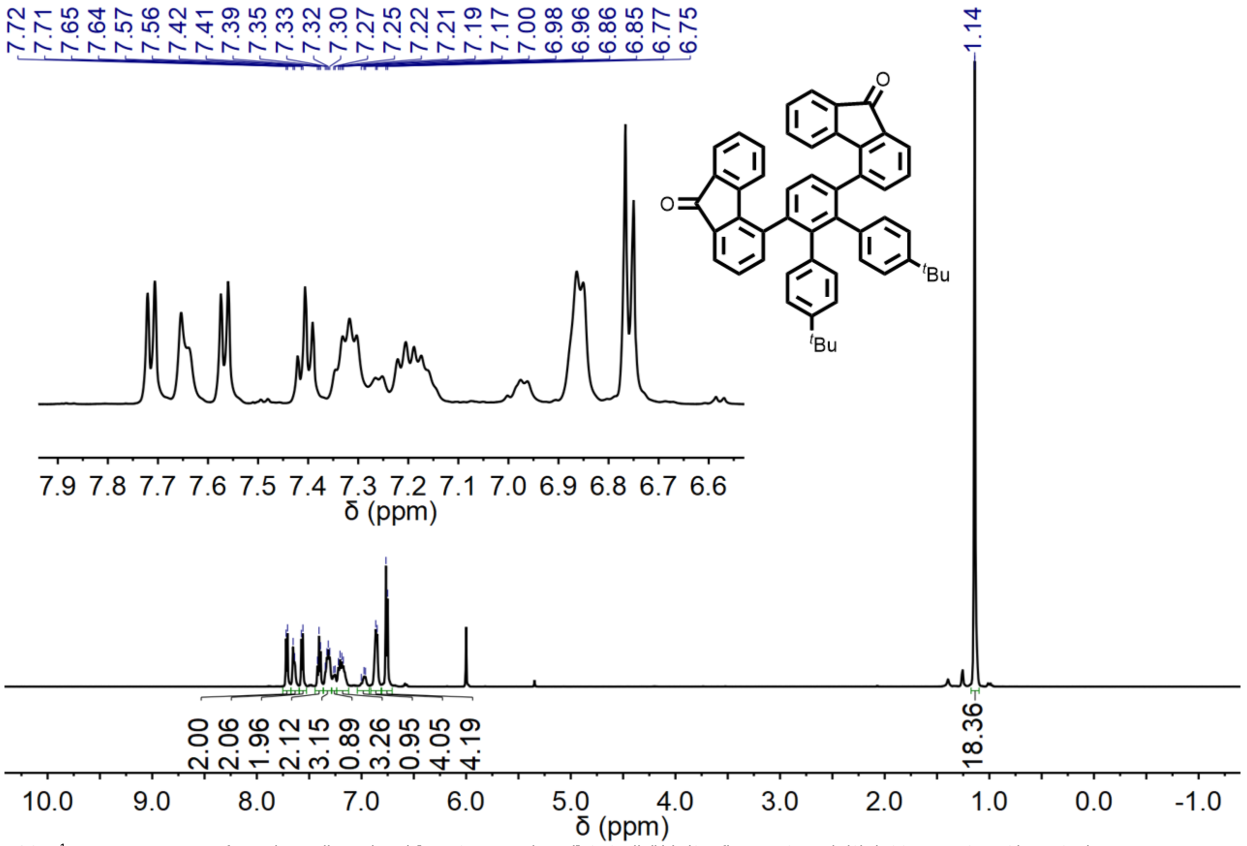

Figure S24. ^{1}H NMR spectrum of 4,4'-(4,4''-di-*tert*-butyl-[1,1':2',1''-terphenyl]-3',6'-diyl)bis(9H-fluoren-9-one) (**6**) (500 MHz, $C_2D_2Cl_4$, 413 K).

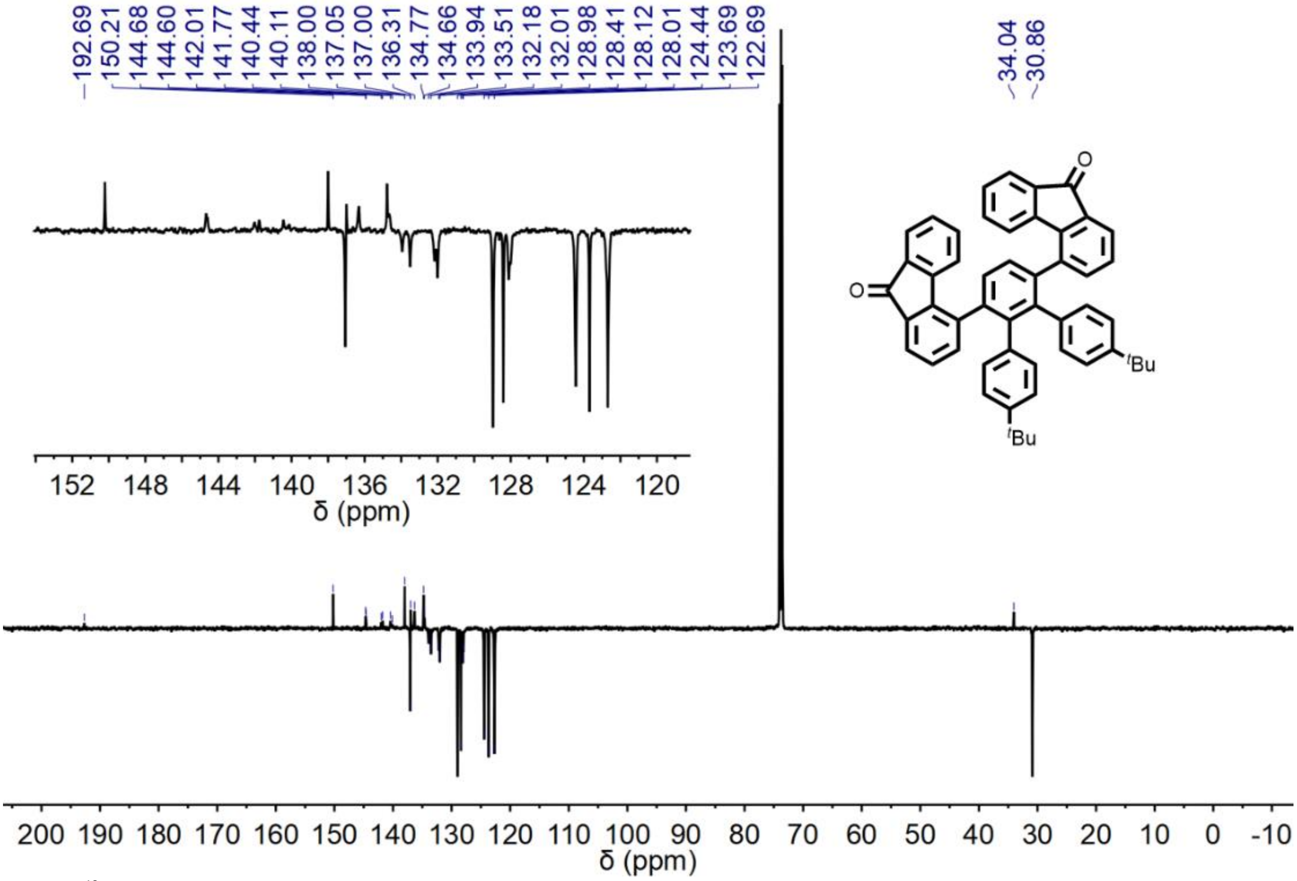

Figure S25. ^{13}C NMR spectrum of 4,4'-(4,4''-di-*tert*-butyl-[1,1':2',1''-terphenyl]-3',6'-diyl)bis(9H-fluoren-9-one) (**6**) (125 MHz, $C_2D_2Cl_4$, 413 K).

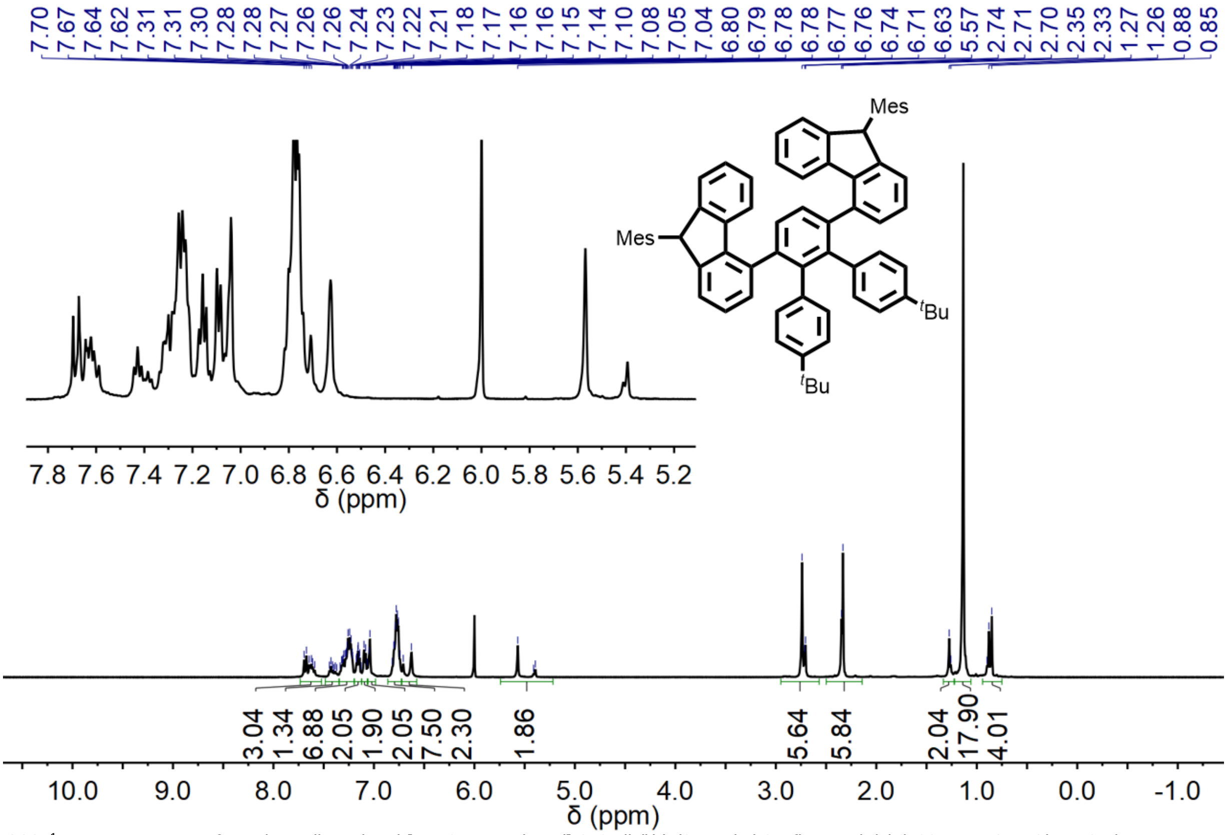

Figure S26. ^{1}H NMR spectrum of 4,4'-(4,4''-di-*tert*-butyl-[1,1':2',1''-terphenyl]-3',6'-diyl)bis(9-mesityl-9H-fluorene) (**7**) (500 MHz, $C_2D_2Cl_4$, 413 K).

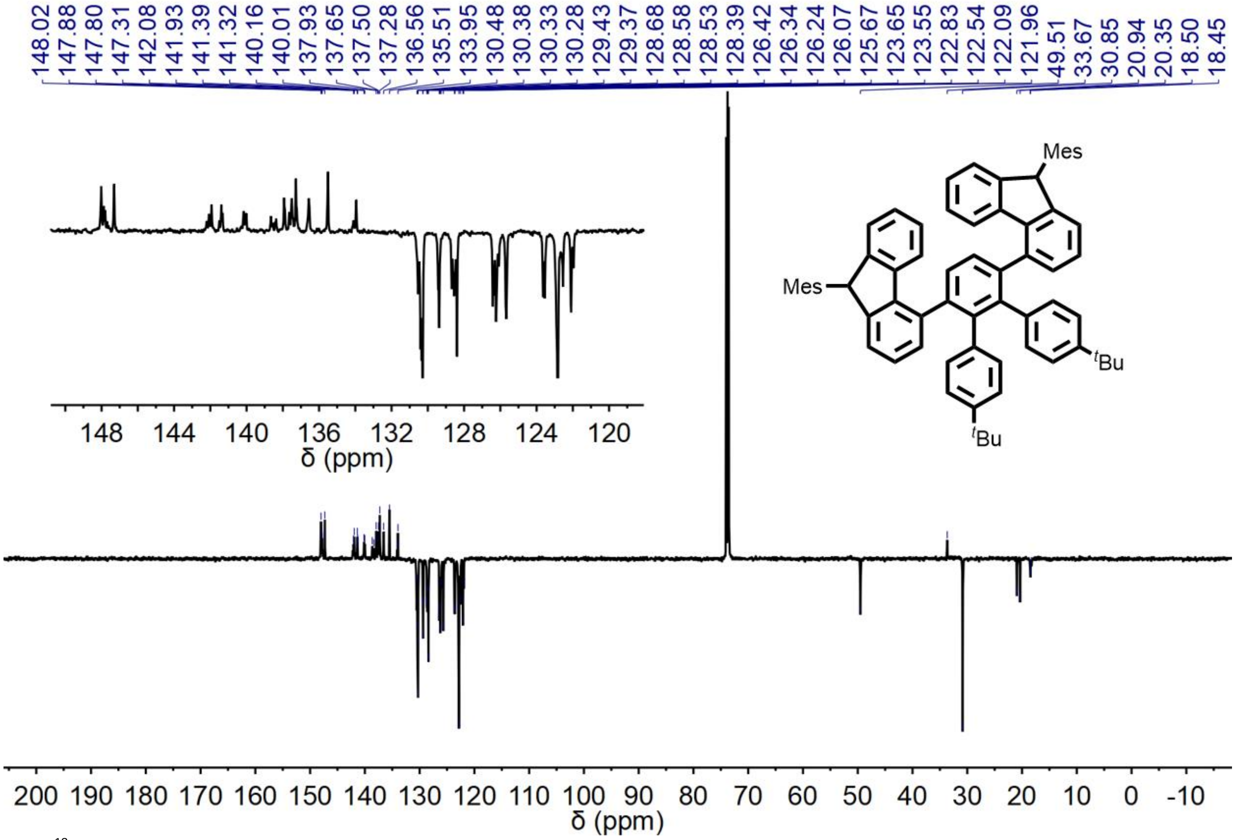

Figure S27. ^{13}C NMR spectrum of 4,4'-(4,4''-di-*tert*-butyl-[1,1':2',1''-terphenyl]-3',6'-diyl)bis(9-mesityl-9H-fluorene) (**7**) (125 MHz, $C_2D_2Cl_4$, 413 K).

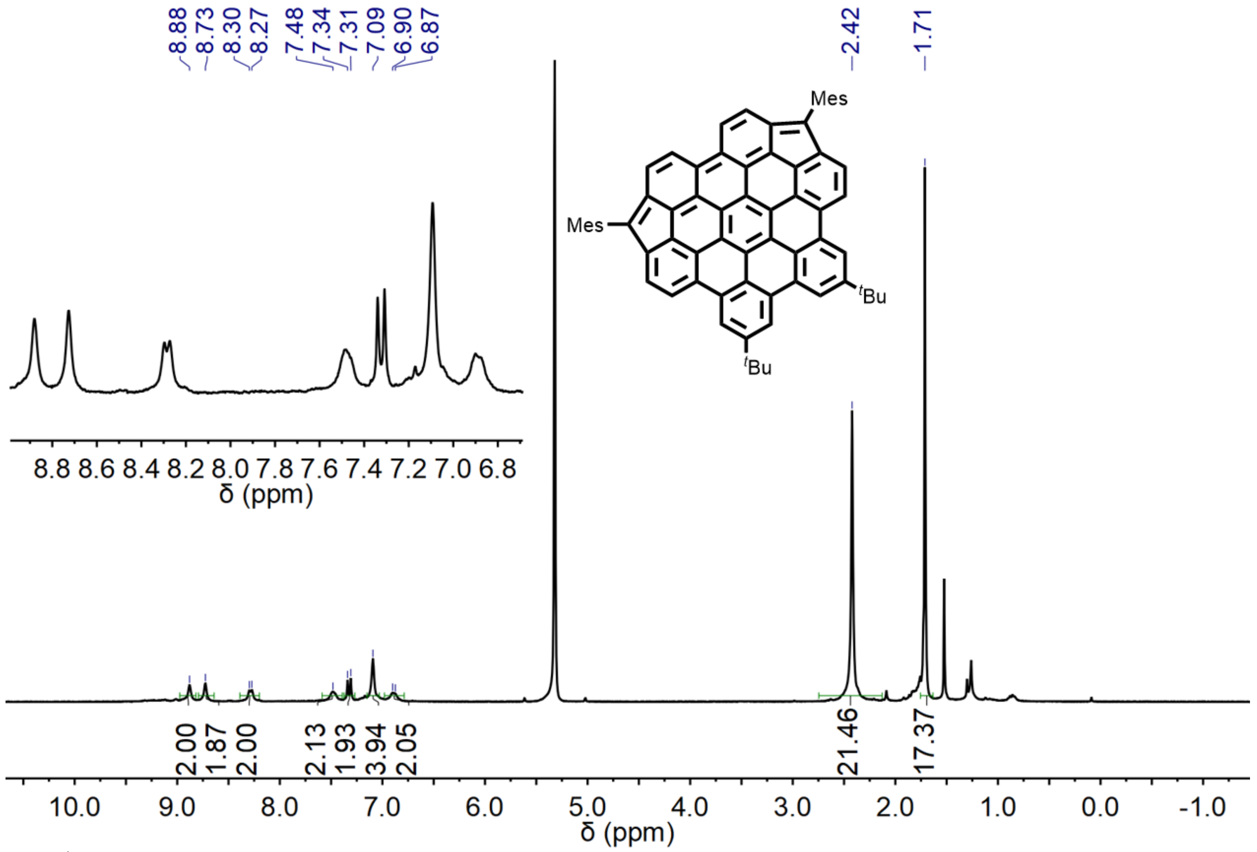

Figure S28. ^{1}H NMR spectrum of *m*PHBC **8** (300 MHz, CD_2Cl_2, 298 K).

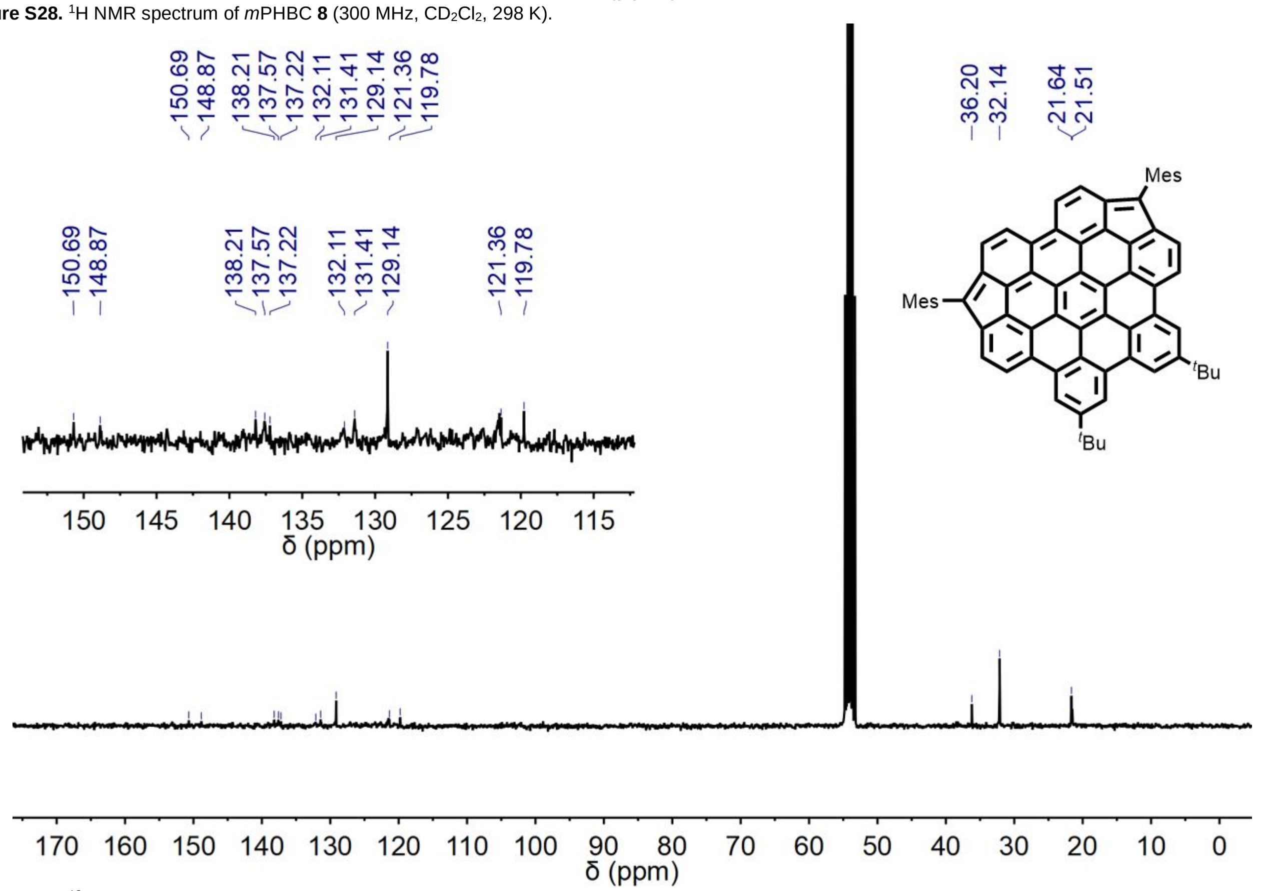

Figure S29. ^{13}C NMR spectrum of *m*PHBC **8** (75 MHz, CD_2Cl_2, 298 K).

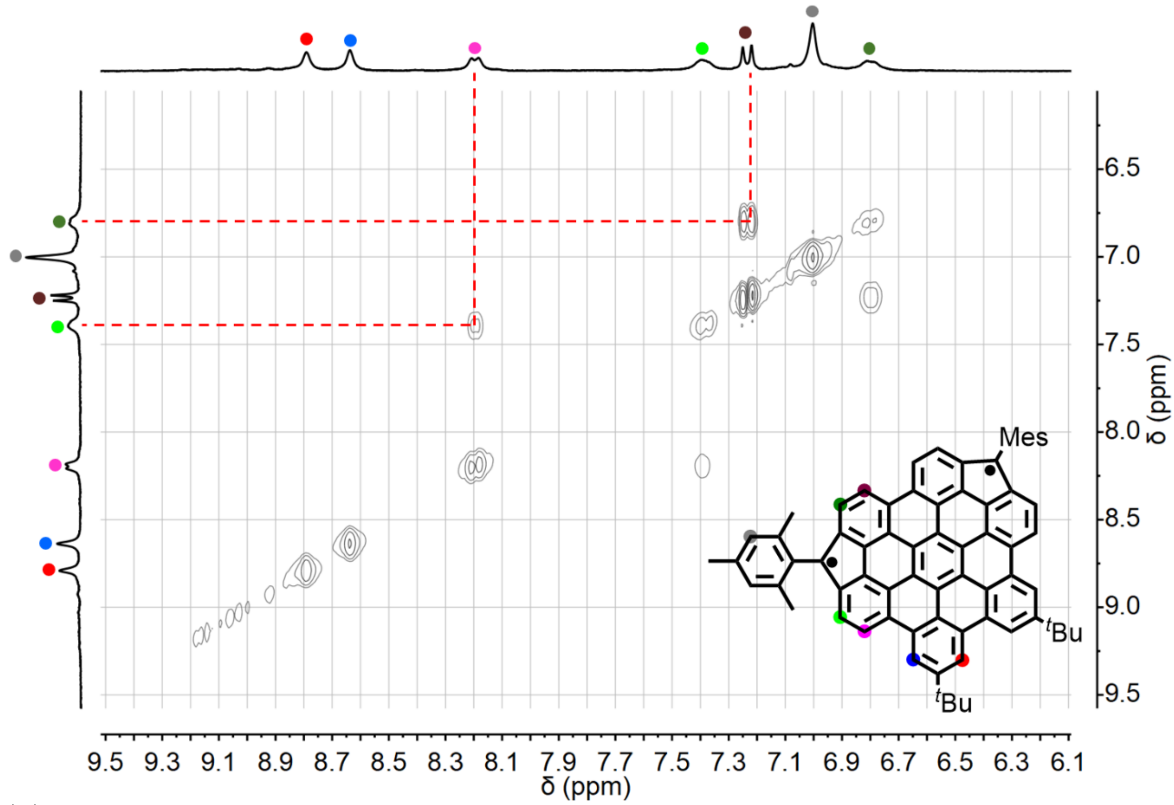

Figure S30. ^{1}H,^{1}H-COSY NMR spectrum of *m*PHBC **8** (300 MHz, CD_2Cl_2, 298 K).

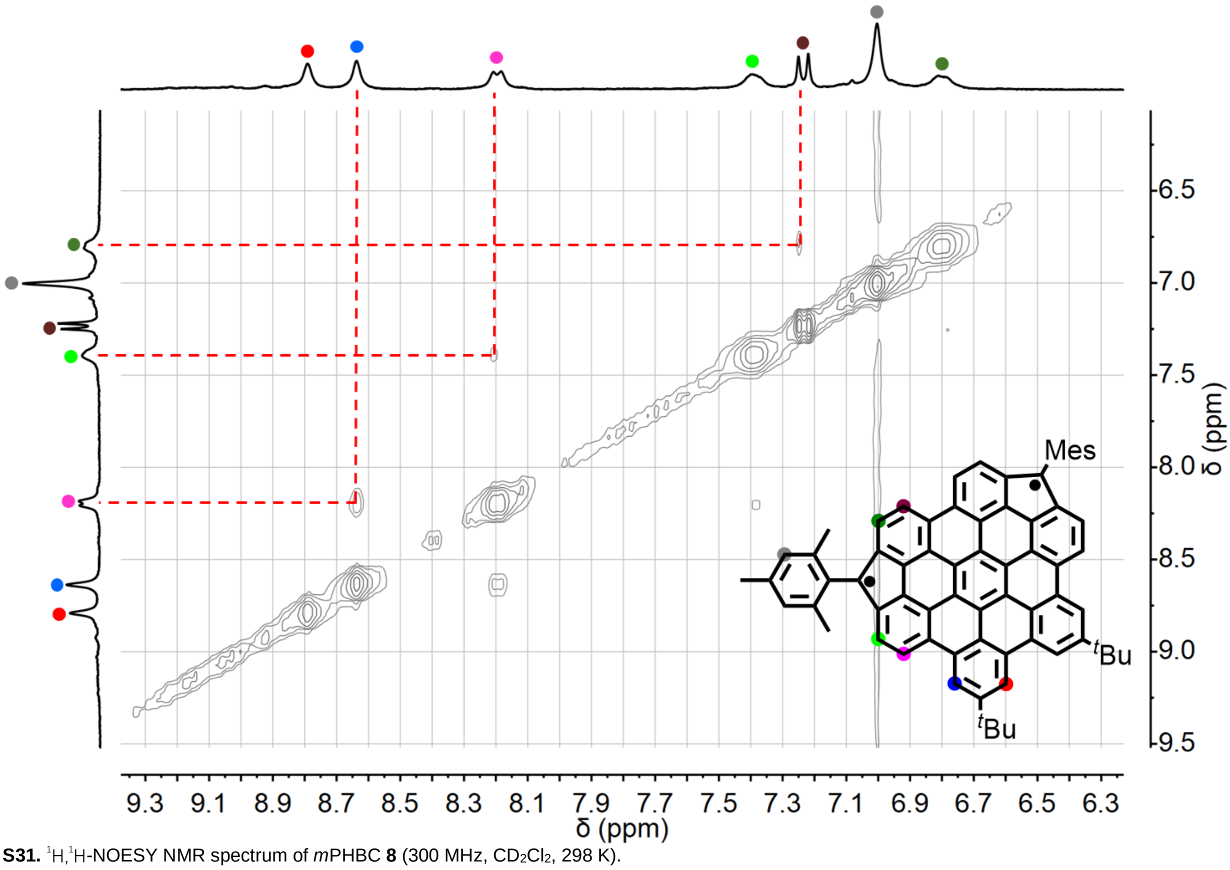

Figure S31. ^{1}H,^{1}H-NOESY NMR spectrum of *m*PHBC **8** (300 MHz, CD_2Cl_2, 298 K).

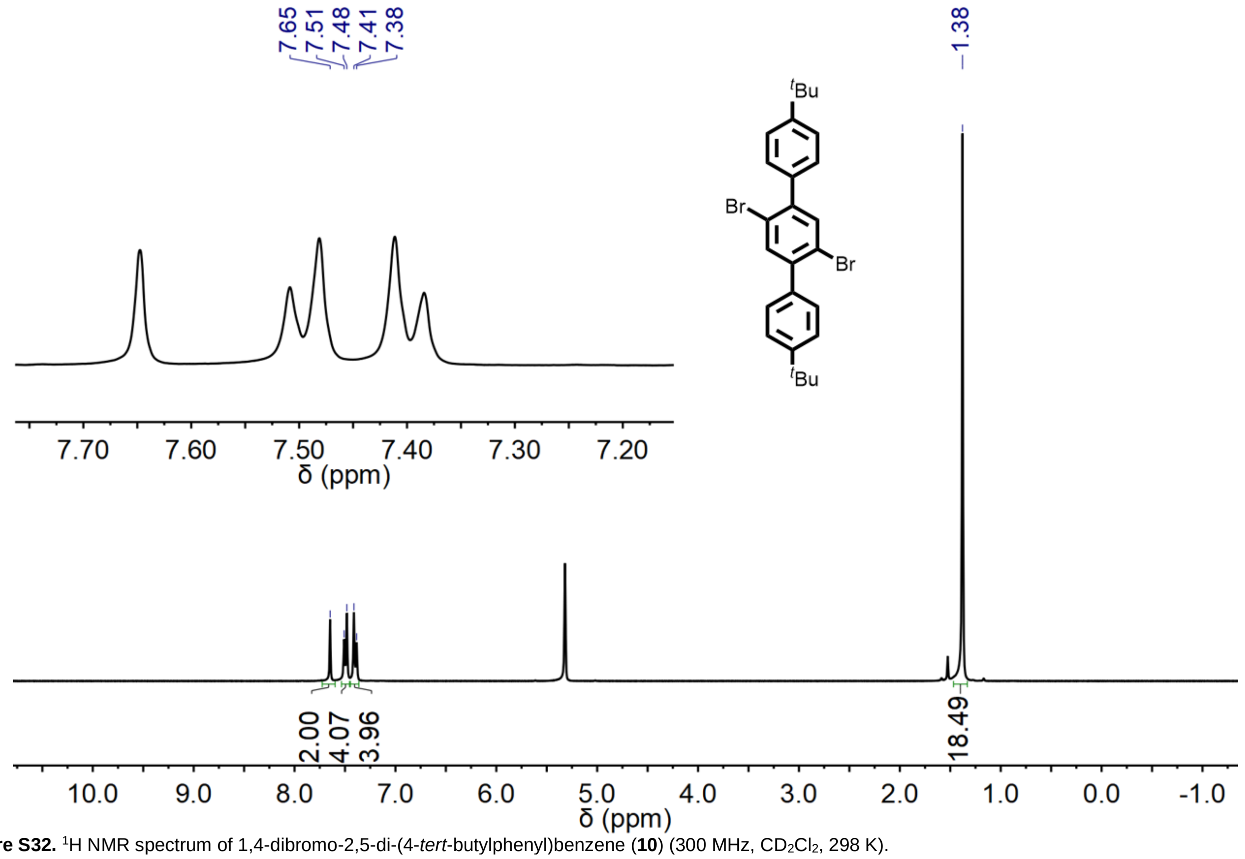

Figure S32. ^{1}H NMR spectrum of 1,4-dibromo-2,5-di-(4-*tert*-butylphenyl)benzene (**10**) (300 MHz, CD_2Cl_2, 298 K).

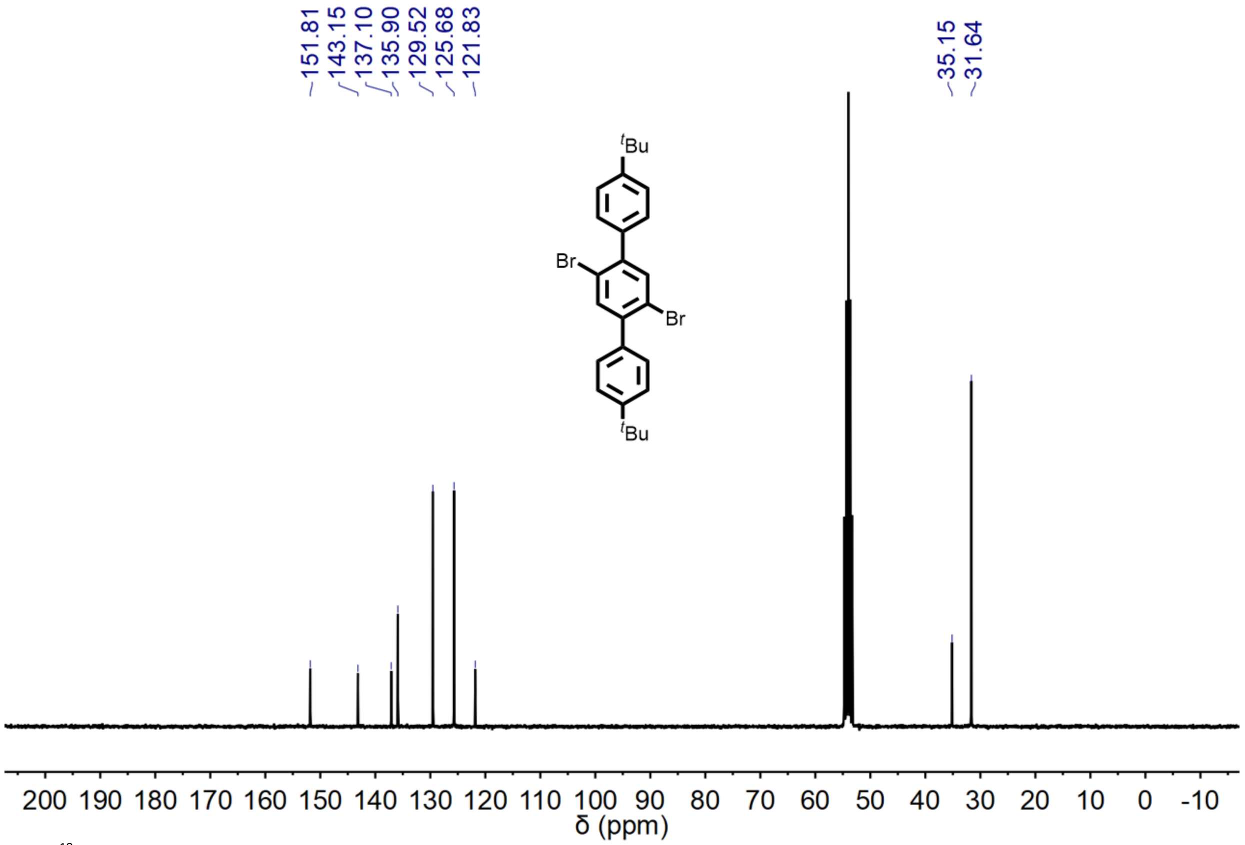

Figure S33. ^{13}C NMR spectrum of 1,4-dibromo-2,5-di-(4-*tert*-butylphenyl)benzene (**10**) (75 MHz, CD_2Cl_2, 298 K).

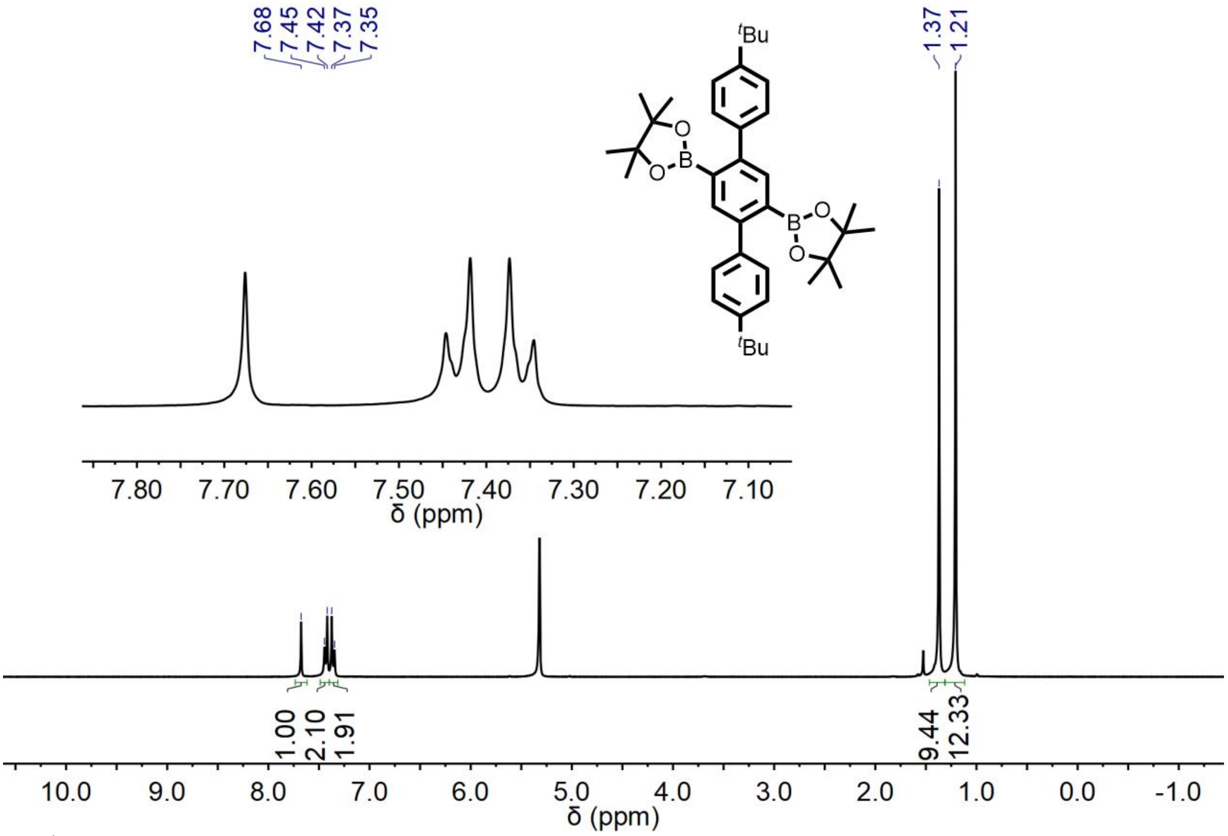

Figure S34. ^{1}H NMR spectrum of 2,5-di-(4-*tert*-butylphenyl)-1,4-bis(4,4,5,5-tetramethyl-1,3,2-dioxaborolan-2-yl)benzene (**11**) (300 MHz, CD_2Cl_2, 298 K).

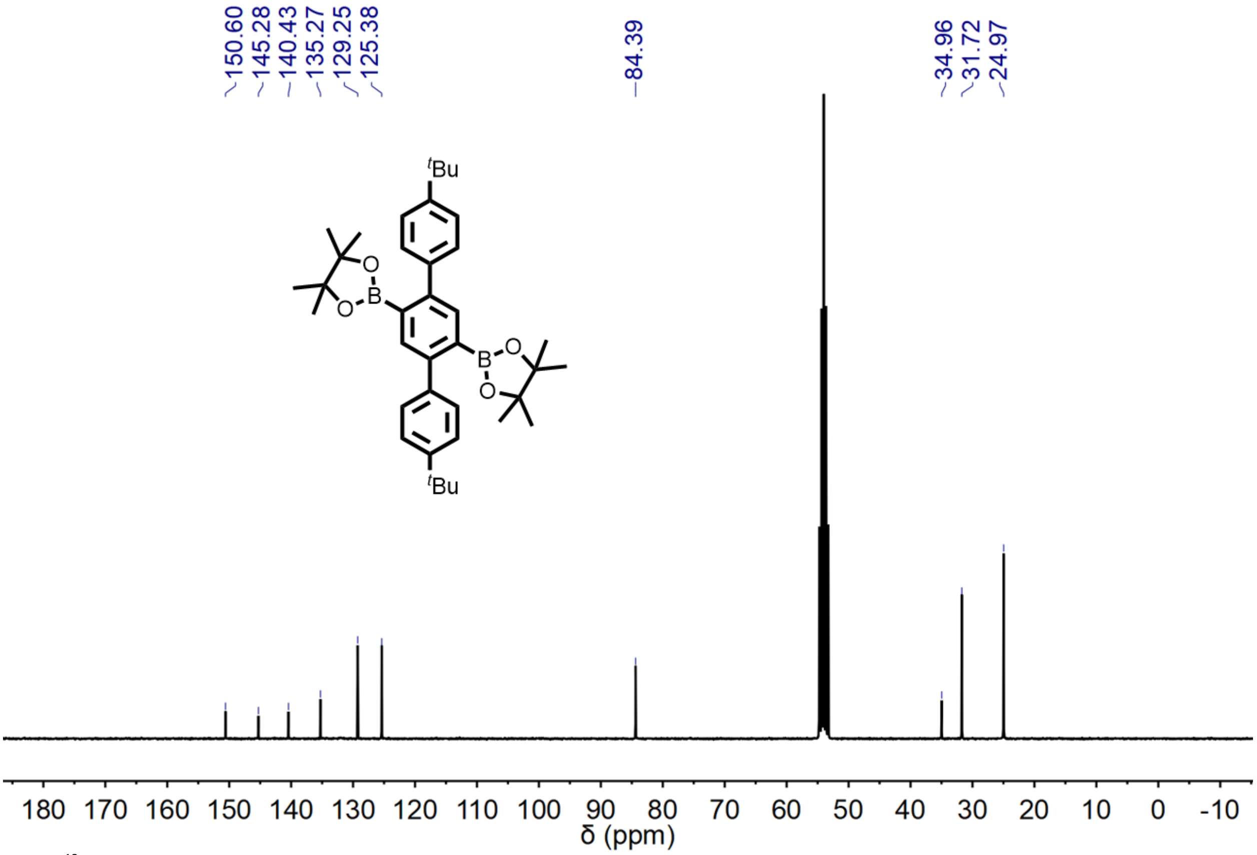

Figure S35. ^{13}C NMR spectrum of 2,5-di-(4-*tert*-butylphenyl)-1,4-bis(4,4,5,5-tetramethyl-1,3,2-dioxaborolan-2-yl)benzene (**11**) (75 MHz, CD_2Cl_2, 298 K).

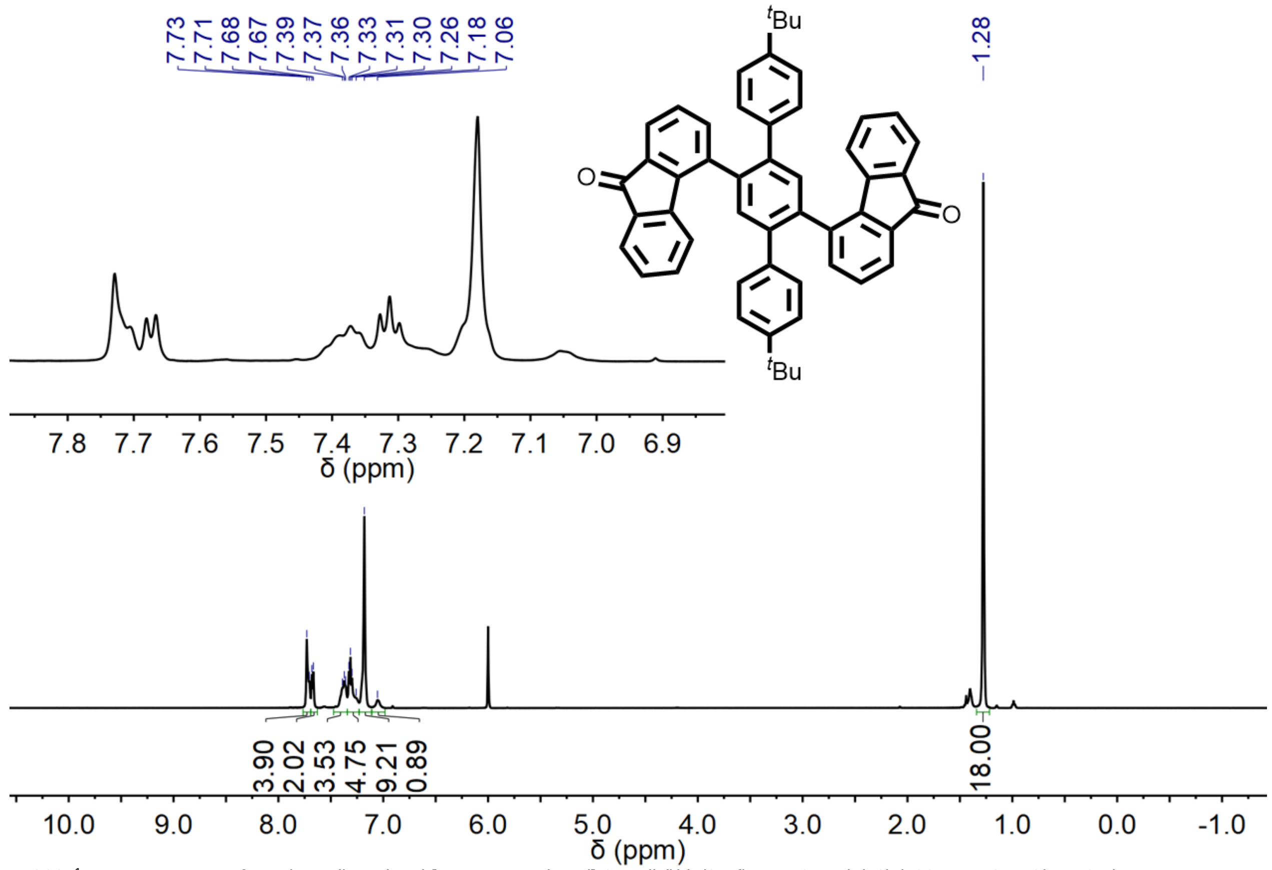

Figure S36. ^{1}H NMR spectrum of 4,4'-(4,4''-di-*tert*-butyl-[1,1':4',1''-terphenyl]-2',5'-diyl)bis(9H-fluoren-9-one) (**12**) (500 MHz, $C_2D_2Cl_4$, 413 K).

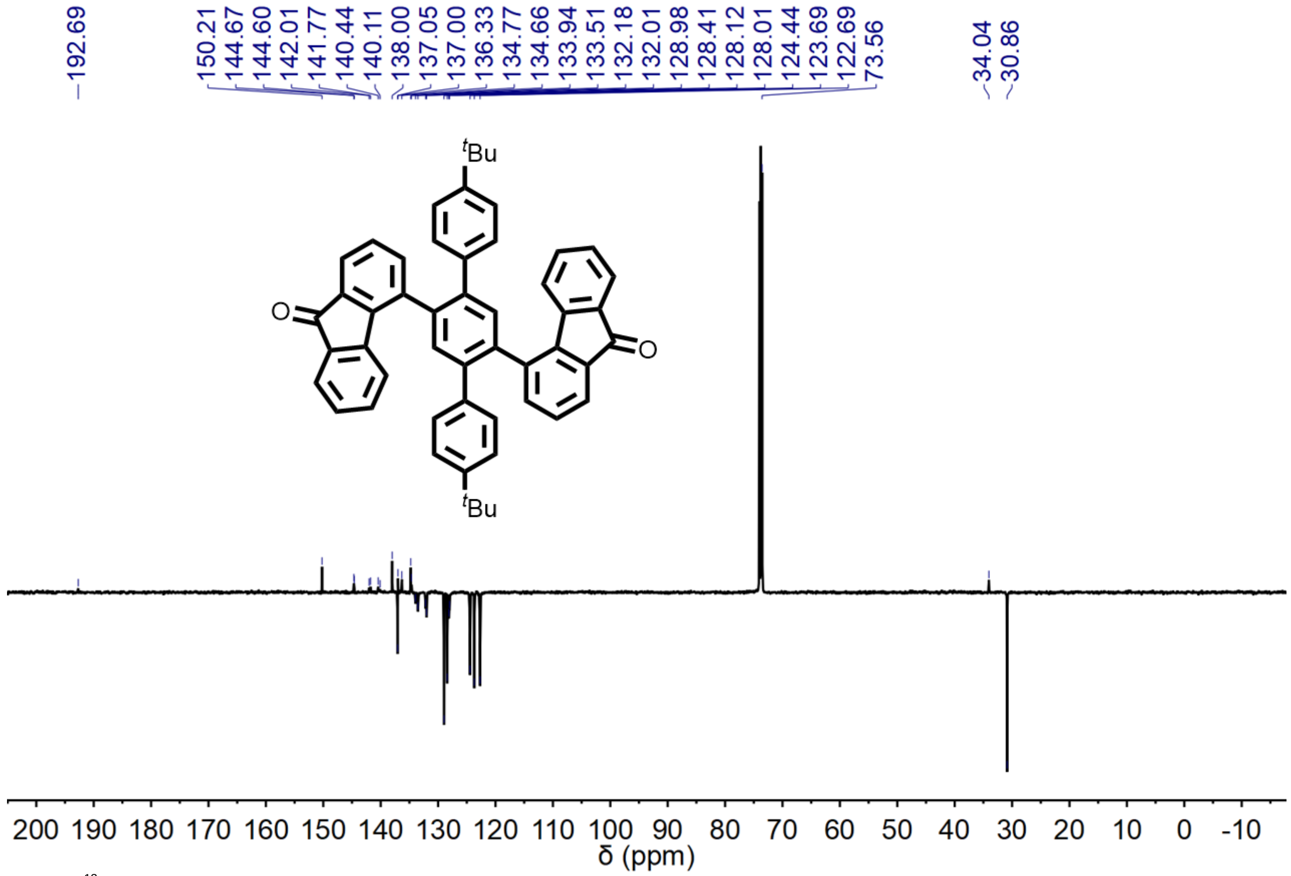

Figure S37. ^{13}C NMR spectrum of 4,4'-(4,4''-di-*tert*-butyl-[1,1':4',1''-terphenyl]-2',5'-diyl)bis(9H-fluoren-9-one) (**12**) (125 MHz, $C_2D_2Cl_4$, 413 K).

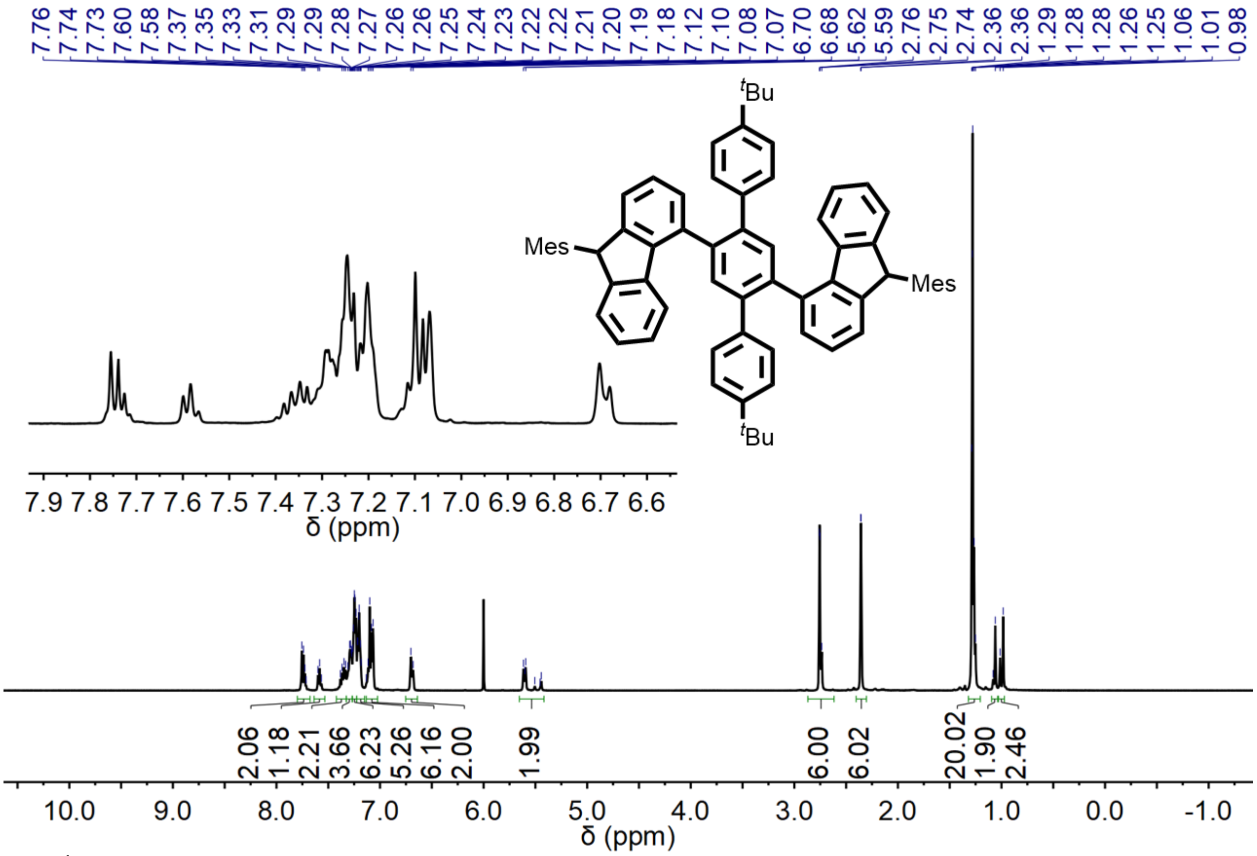

Figure S38. ^{1}H NMR spectrum of 4,4'-(4,4''-di-*tert*-butyl-[1,1':4',1''-terphenyl]-2',5'-diyl)bis(9-mesityl-9H-fluorene) (**13**) (500 MHz, $C_2D_2Cl_4$, 413 K).

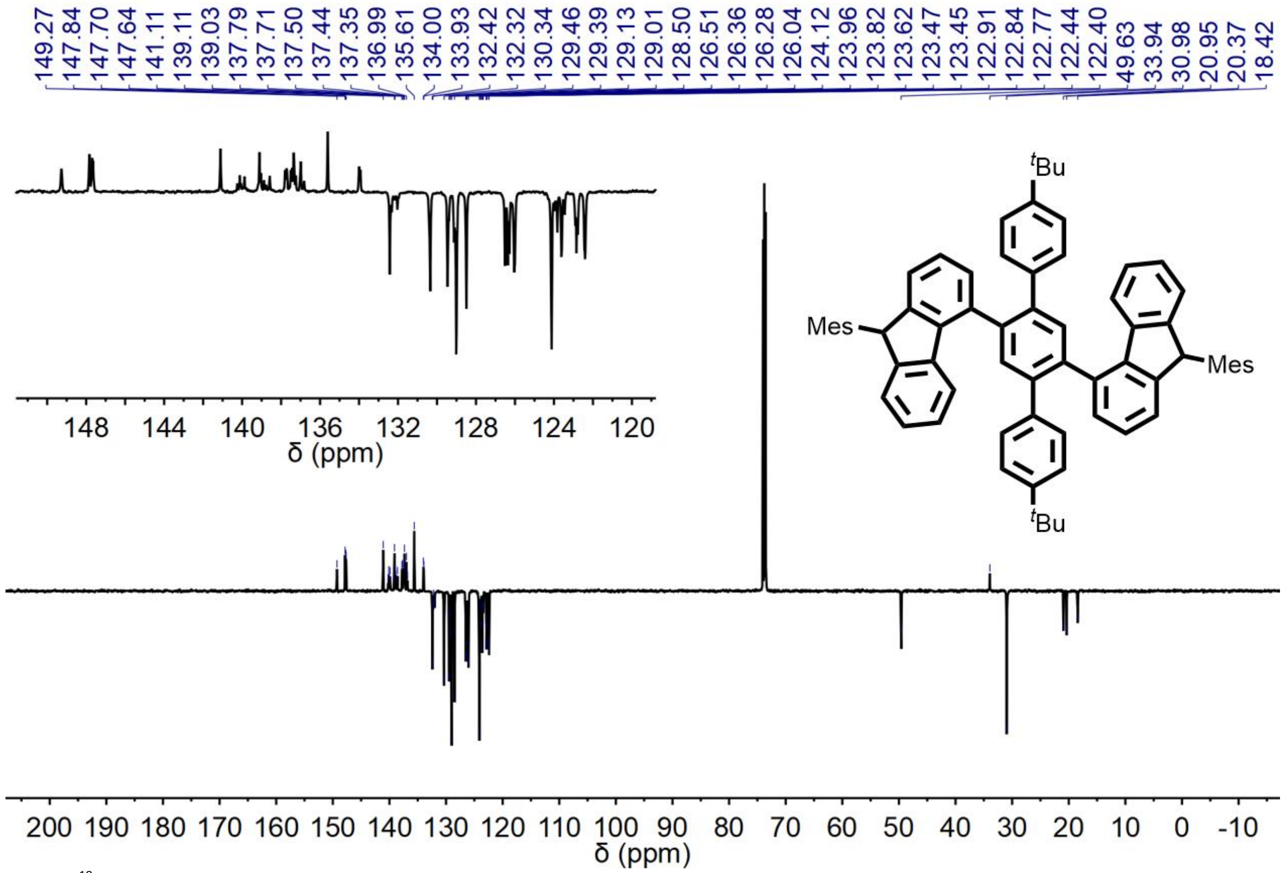

Figure S39. ^{13}C NMR spectrum of 4,4'-(4,4''-di-*tert*-butyl-[1,1':4',1''-terphenyl]-2',5'-diyl)bis(9-mesityl-9H-fluorene) (**13**) (125 MHz, $C_2D_2Cl_4$, 413 K)).

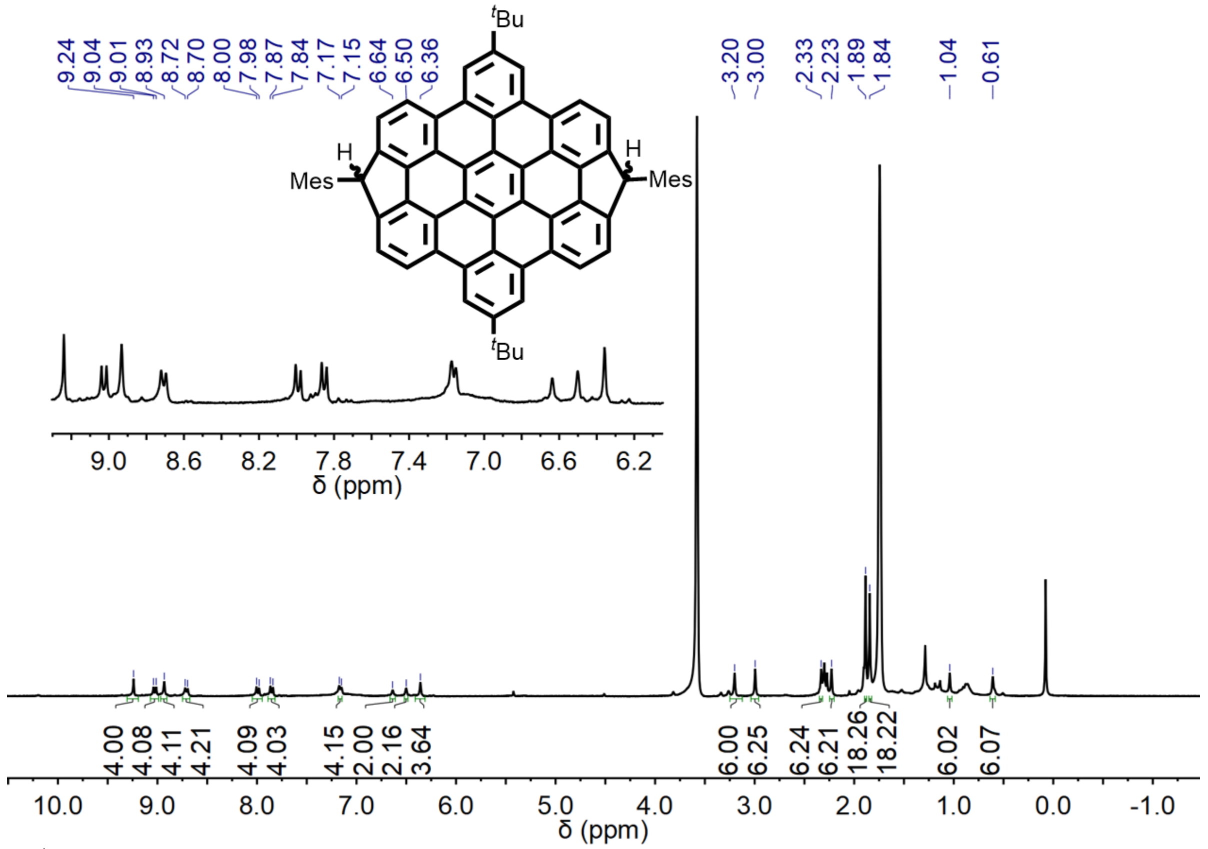

Figure S40. ^{1}H NMR spectrum of 2,10-di-*tert*-butyl-6,14-dimesityl-6,14-dihydrodibenzo[*ef*,*no*]difluoreno[3,4,5,6-*hijkl*:3',4',5',6'-*qrabc*]coronene (**14**) (300 MHz, THF-d_8:CS_2 = 1:1, 298 K). The isomeric mixture was used for further experiments.

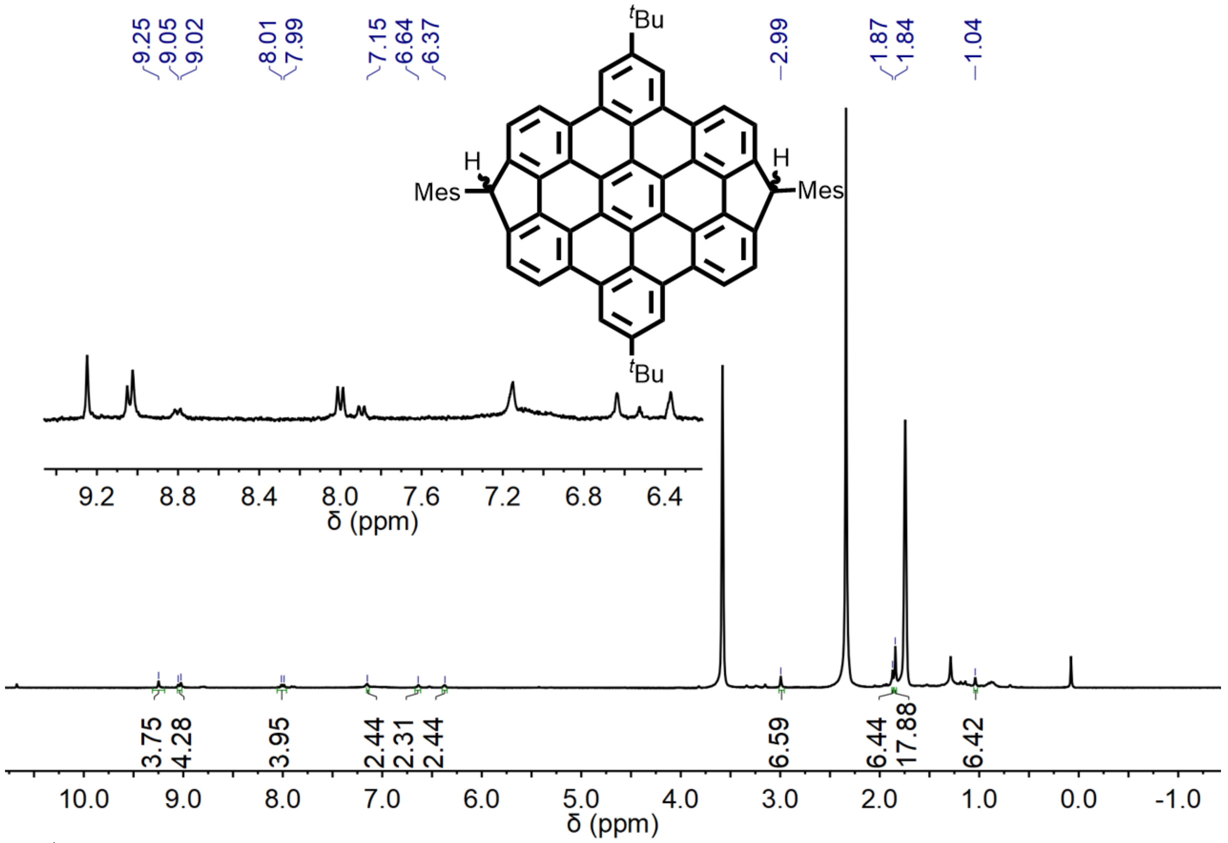

Figure S41. ^{1}H NMR spectrum of 2,10-di-*tert*-butyl-6,14-dimesityl-6,14-dihydrodibenzo[*ef*,*no*]difluoreno[3,4,5,6-*hijkl*:3',4',5',6'-*qrabc*]coronene (**14-I**) (300 MHz, THF-d_8:CS_2 = 1:1, 298 K). The impurity peaks are from the other isomer **14-II**, which could not be completely removed due to the very low solubility of both isomers. The peak shifts are ascribed to the differences in the concentration in each measurement.[7]

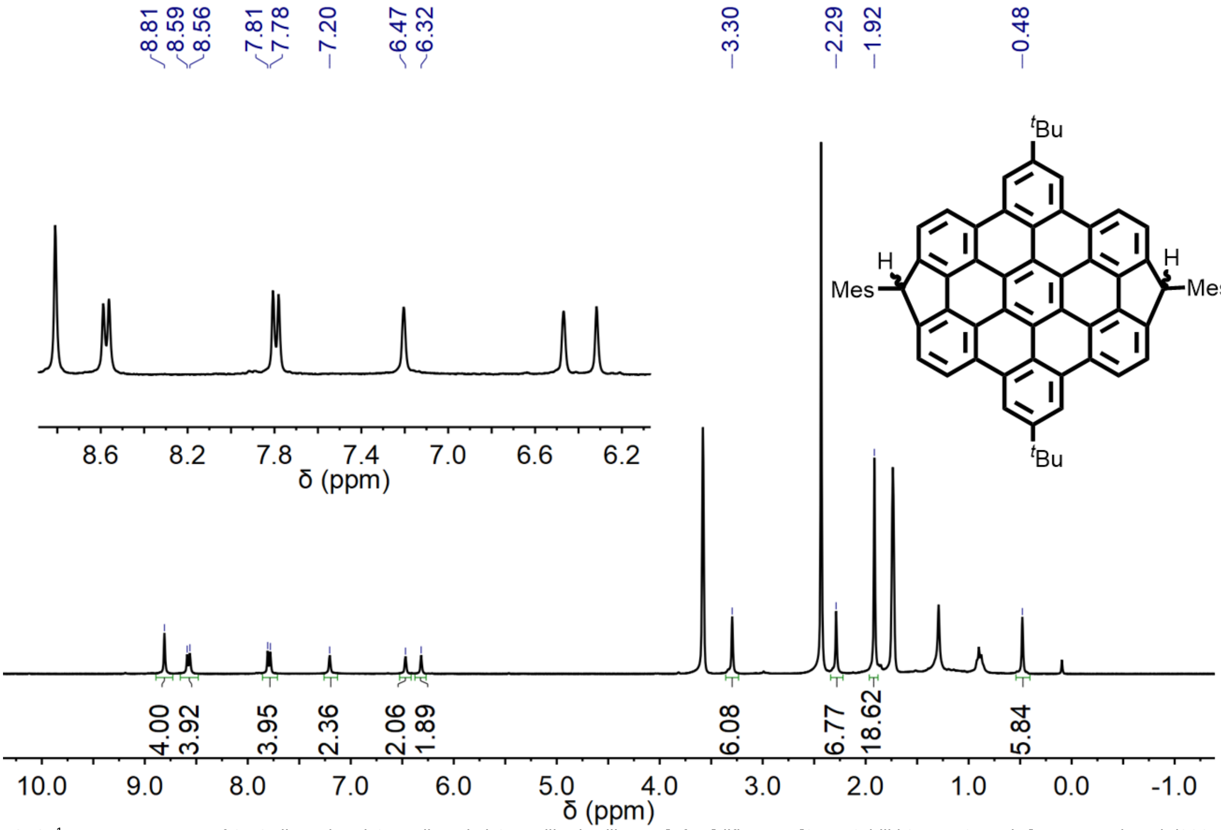

Figure S42. ^{1}H NMR spectrum of 2,10-di-*tert*-butyl-6,14-dimesityl-6,14-dihydrodibenzo[*ef*,*no*]difluoreno[3,4,5,6-*hijkl*:3',4',5',6'-*qrabc*]coronene (**14-II**) (300 MHz, THF-d_8:CS_2 = 1:1, 298 K).

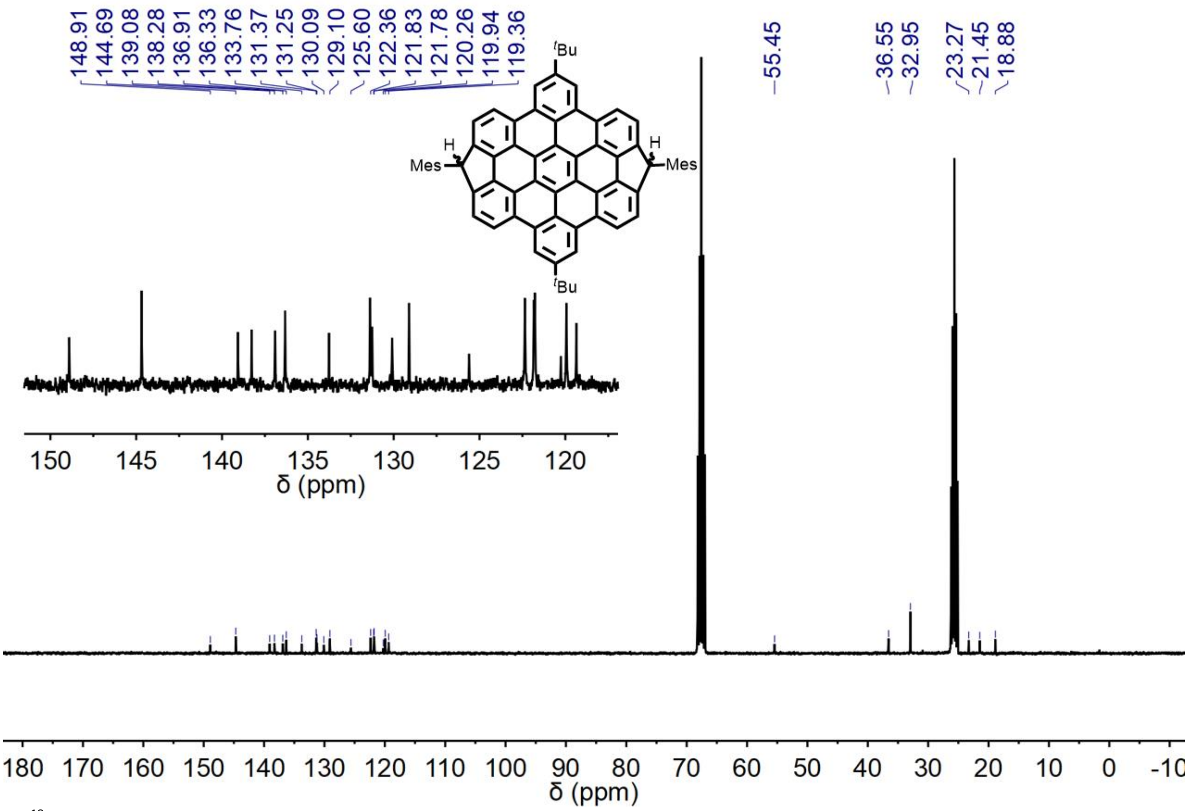

Figure S43. ^{13}C NMR spectrum of 2,10-di-*tert*-butyl-6,14-dimesityl-6,14-dihydrodibenzo[*ef*,*no*]difluoreno[3,4,5,6-*hijkl*:3',4',5',6'-*qrabc*]coronene (**14-II**) (75 MHz, THF-d_8:CS_2 = 1:1, 298 K).

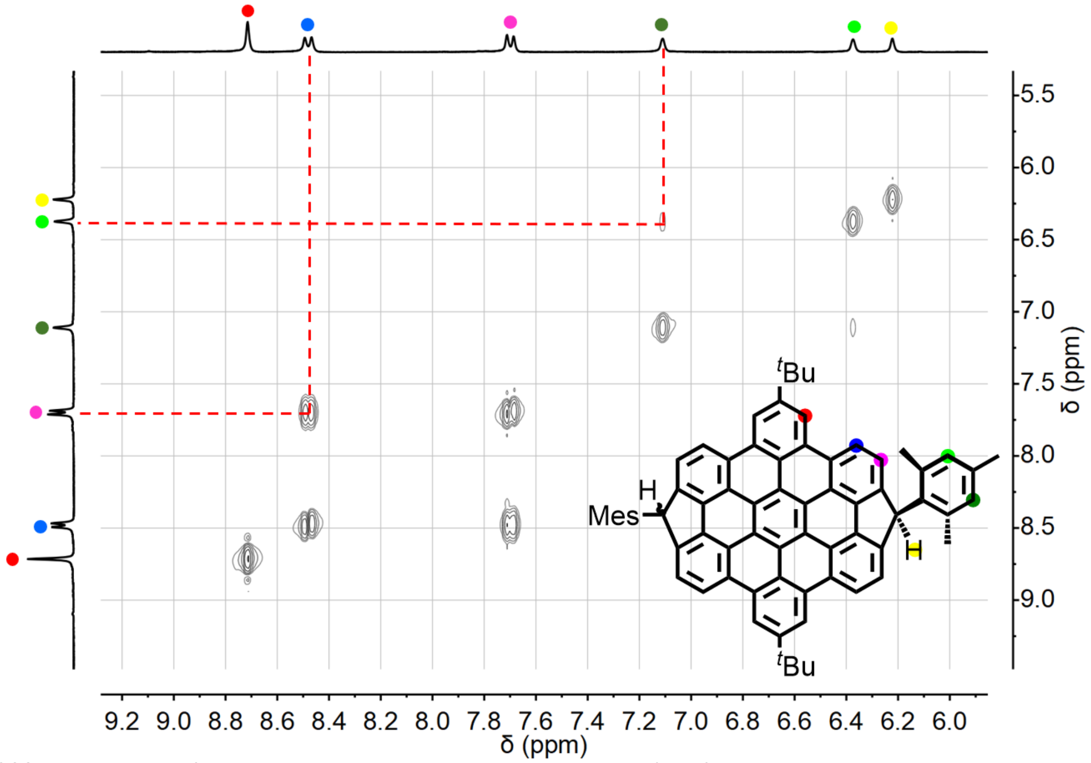

Figure S44. ^{1}H,^{1}H-COSY NMR spectrum of 2,10-di-*tert*-butyl-6,14-dimesityl-6,14-dihydrodibenzo[*ef*,*no*]difluoreno[3,4,5,6-*hijkl*:3',4',5',6'-*qrabc*]coronene (**14-II**) (300 MHz, THF-d_8:CS_2 = 1:1, 298 K).

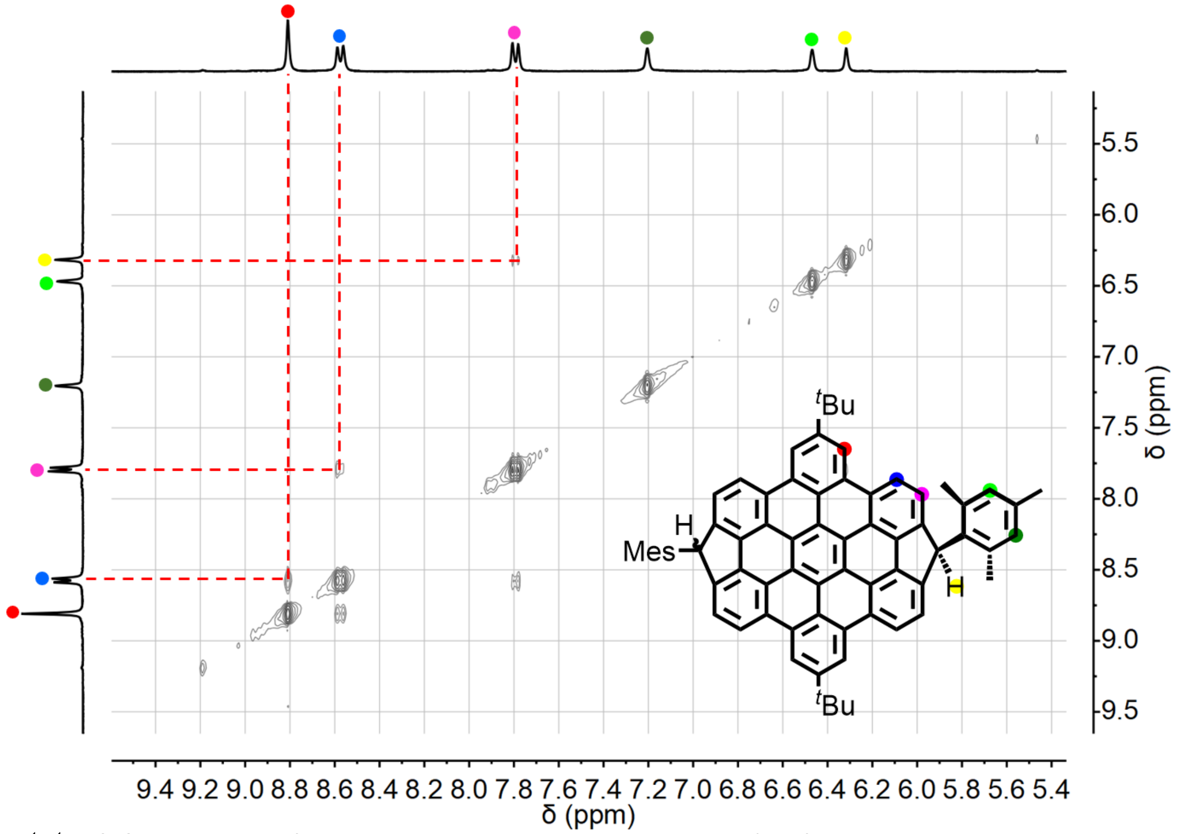

Figure S45. ^{1}H,^{1}H-NOESY NMR spectrum of 2,10-di-*tert*-butyl-6,14-dimesityl-6,14-dihydrodibenzo[*ef*,*no*]difluoreno[3,4,5,6-*hijkl*:3',4',5',6'-*qrabc*]coronene (**14-II**) (300 MHz, THF-d_8:CS_2 = 1:1, 298 K).

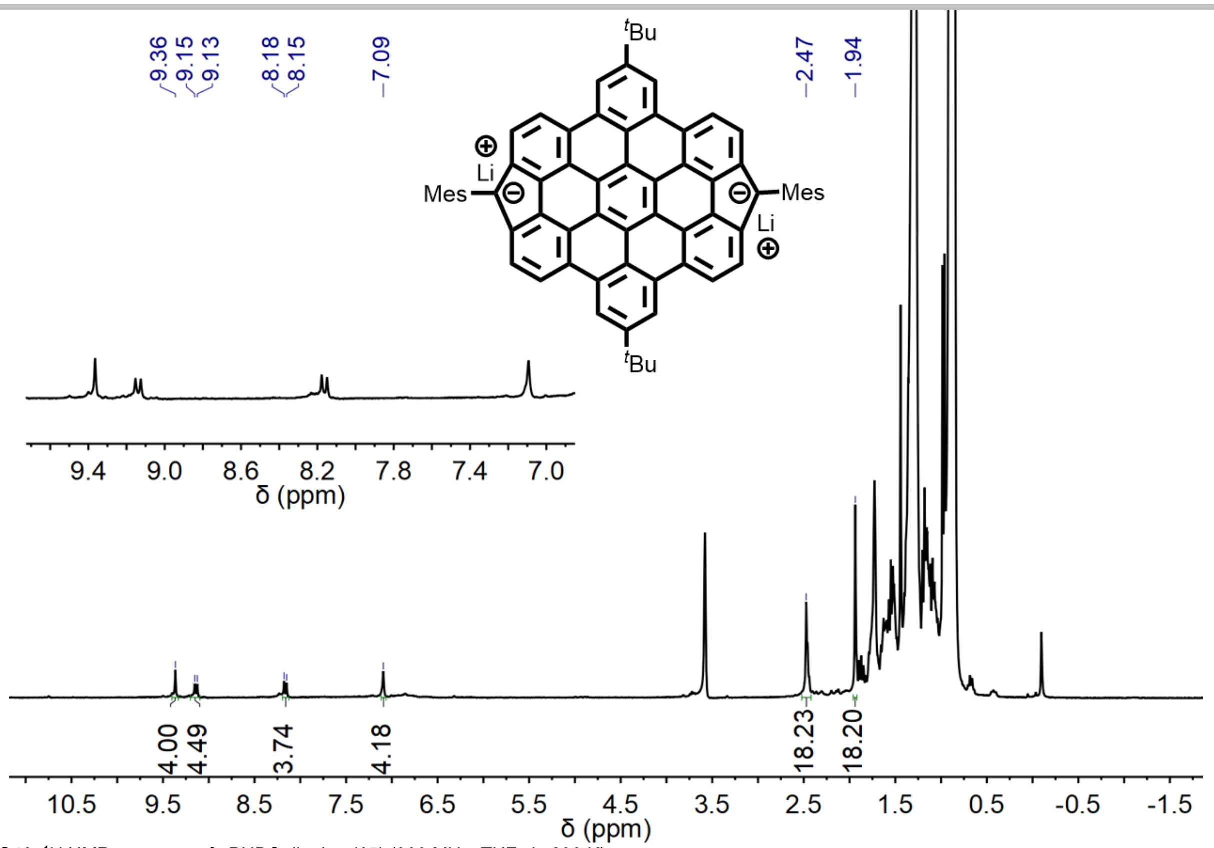

Figure S46. ^{1}H NMR spectrum of *p*PHBC dianion (**15**) (300 MHz, THF-d_8, 298 K).

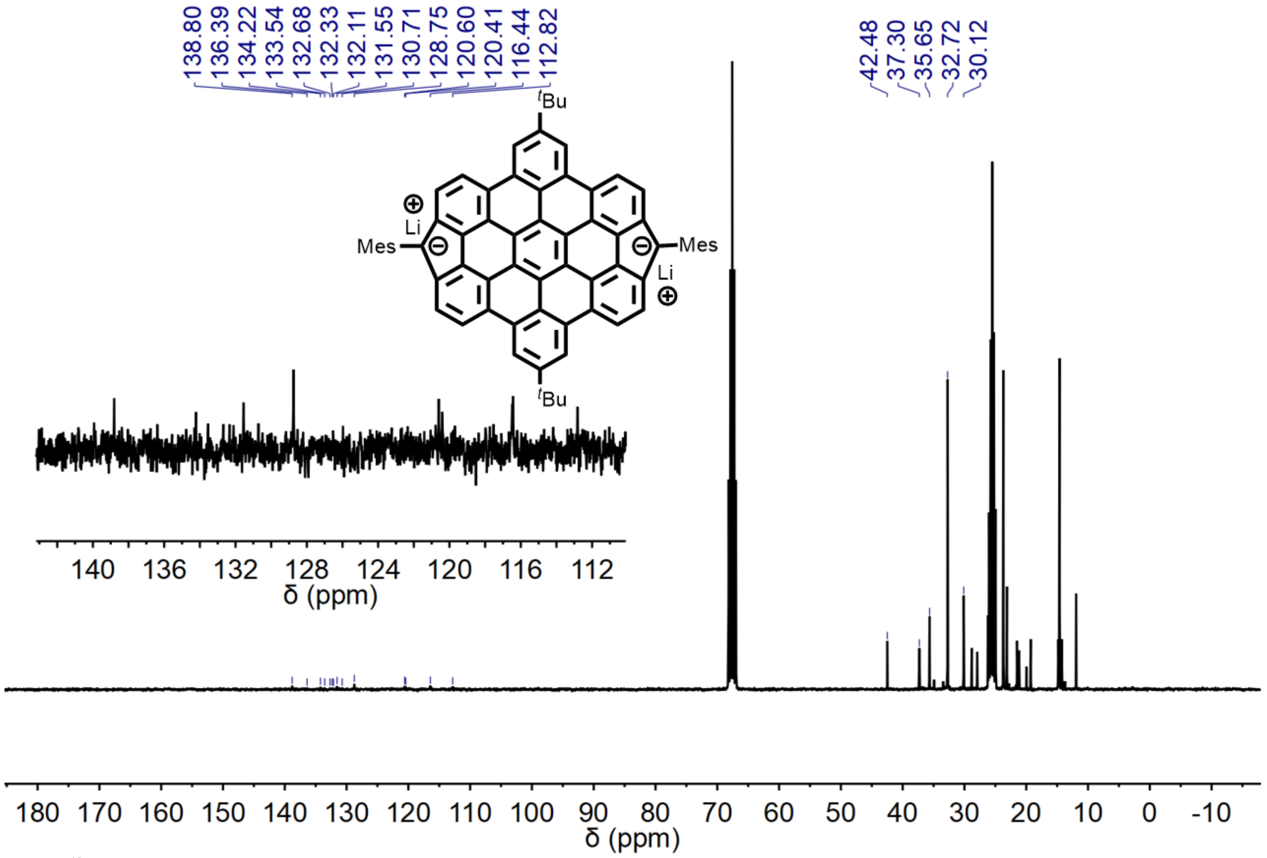

Figure S47. ^{13}C NMR spectrum of *p*PHBC dianion (**15**) (75 MHz, THF-d_8, 298 K).

7. References

[1] M. Di Giovannantonio, O. Deniz, J. I. Urgel, R. Widmer, T. Dienel, S. Stolz, C. Sanchez-Sanchez, M. Muntwiler, T. Dumslaff, R. Berger, A. Narita, X. Feng, K. Müllen, P. Ruffieux, R. Fasel, *ACS Nano* **2018**, *12*, 74-81.

[2] Z. Qiu, S. Asako, Y. Hu, C. W. Ju, T. Liu, L. Rondin, D. Schollmeyer, J. S. Lauret, K. Müllen, A. Narita, *J. Am. Chem. Soc.* **2020**, *142*, 14814-14819.

[3] J. Liu, A. Narita, S. Osella, W. Zhang, D. Schollmeyer, D. Beljonne, X. Feng, K. Müllen, *J. Am. Chem. Soc.* **2016**, *138*, 2602-2608.

[4] R. D. Gaussian 09, M. J. Frisch, G. W. Trucks, H. B. Schlegel, G. E. Scuseria, M. A. Robb, J. R. Cheeseman, G. Scalmani, V. Barone, B. Mennucci, G. A. Petersson, H. Nakatsuji, M. Caricato, X. Li, H. P. Hratchian, A. F. Izmaylov, J. Bloino, G. Zheng, J. L. Sonnenberg, M. Hada, M. Ehara, K. Toyota, R. Fukuda, J. Hasegawa, M. Ishida, T. Nakajima, Y. Honda, O. Kitao, H. Nakai, T. Vreven, J. A. Montgomery, Jr., J. E. Peralta, F. Ogliaro, M. Bearpark, J. J. Heyd, E. Brothers, K. N. Kudin, V. N. Staroverov, T. Keith, R. Kobayashi, J. Normand, K. Raghavachari, A. Rendell, J. C. Burant, S. S. Iyengar, J. Tomasi, M. Cossi, N. Rega, J. M. Millam, M. Klene, J. E. Knox, J. B. Cross, V. Bakken, C. Adamo, J. Jaramillo, R. Gomperts, R. E. Stratmann, O. Yazyev, A. J. Austin, R. Cammi, C. Pomelli, J. W. Ochterski, R. L. Martin, K. Morokuma, V. G. Zakrzewski, G. A. Voth, P. Salvador, J. J. Dannenberg, S. Dapprich, A. D. Daniels, O. Farkas, J. B. Foresman, J. V. Ortiz, J. Cioslowski, and D. J. Fox, Gaussian, Inc., Wallingford CT, 2013.

[5] S. Yamanaka, M. Okumura, M. Nakano, K. Yamaguchi, *J. Mol. Str.* **1994**, *310*, 205-218.

[6] a) Z. Chen, C. S. Wannere, C. Corminboeuf, R. Puchta, P. Schleyer, *Chem. Rev.* **2005**, *105*, 3842-3888; b) H. Fallah-Bagher-Shaidaei, C. S. Wannere, C. Corminboeuf, R. Puchta, P. Schleyer, *Org. Lett.* **2006**, *8*, 863-866.

[7] J. Wu, A. Fechtenkotter, J. Gauss, M. D. Watson, M. Kastler, C. Fechtenkotter, M. Wagner, K. Mullen, *J. Am. Chem. Soc.* **2004**, *126*, 11311-11321.

8. Cartesian Coordinates and Spin Densities

*m*PHBC **8**

C	-0.70666000	-0.83938000	0.00094200
C	-1.40332200	0.39582300	-0.00068500
C	-0.70778000	1.62517500	-0.00072100
C	0.70582700	1.62003000	0.00144700
C	1.39212000	0.38560900	0.00173600
C	0.68609300	-0.84442600	0.00036200
C	1.34920400	-2.11967500	0.00270200
C	2.81854000	0.38091600	0.00218400
C	1.46049200	2.84681300	0.00369900
C	-1.45375800	2.85656700	-0.00336200
C	-2.82911700	0.40251600	-0.00124100
C	-1.37978400	-2.10923700	-0.00131500
C	-2.89256700	2.85570300	-0.00730900
C	-3.55727400	4.08880000	-0.00907700
C	-2.87228800	5.30796400	-0.00765500
C	-1.47190700	5.27877800	-0.00443900
C	-0.73212900	4.08636400	-0.00203200
C	0.75085700	4.07969400	0.00190100
C	1.49812200	5.27243300	0.00388500
C	2.89409900	5.28974500	0.00731400
C	3.57074700	4.06052500	0.00918200
C	2.90237700	2.83441800	0.00767000
C	3.62116500	1.54708400	0.00941200
C	5.02239500	1.32312000	0.02138200
C	5.61723200	0.03907600	0.02183000
C	4.81091600	-1.11698800	0.00192200
C	3.43249900	-0.85692400	-0.00016200
C	2.72132200	-2.06906700	0.00618200
C	3.62554100	-3.15784300	0.01716500
C	2.98993200	-4.43397100	0.00655500
C	1.59271300	-4.53125400	0.00183800
C	0.70372200	-3.40542700	0.00225200
C	-0.74431100	-3.39999100	-0.00084400
C	-1.64212000	-4.51875300	-0.00051400
C	-3.03867900	-4.41038500	-0.00536800
C	-3.66434700	-3.12945400	-0.01603900
C	-2.75151800	-2.04797300	-0.00489800
C	-3.45311600	-0.83034100	0.00124800
C	-4.83338800	-1.07911900	-0.00109900
C	-5.63028300	0.08391600	-0.02142600
C	-5.02545600	1.36284800	-0.02104900
C	-3.62182900	1.57563200	-0.00882300
C	-4.99044300	-2.55435200	-0.01263000
C	4.95599600	-2.59373000	0.01349700
C	3.70596900	6.60008500	0.00865000
C	2.80786900	7.85153300	0.00659900
C	4.59679900	6.65149800	1.27292300
C	4.60164500	6.65085400	-1.25224100
C	-3.60185400	6.66684600	-0.00928700
C	-5.13407100	6.51035800	-0.01187900
C	-3.20398800	7.46706400	1.25383900
C	-3.19956900	7.46628600	-1.27149700
C	6.23312700	-3.33980600	0.03045400
C	6.61471700	-4.07279000	1.17881600
C	7.83021700	-4.76317800	1.17341600
C	8.68042200	-4.76002400	0.06479200
C	8.28745300	-4.02929500	-1.05982400
C	7.08466600	-3.31915400	-1.09980400
C	6.69926900	-2.57700200	-2.35988000
C	5.75552300	-4.09188600	2.42372000
C	9.97153600	-5.54337200	0.07153900
C	-6.27428100	-3.28880900	-0.03012400
C	-7.12651200	-3.25950200	1.09940700
C	-8.33681200	-3.95673900	1.05813100
C	-8.73665000	-4.68270600	-0.06711100
C	-7.88537900	-4.69483800	-1.17489200
C	-6.66258100	-4.01749700	-1.17899200
C	-5.80264700	-4.04501900	-2.42322600
C	-6.73464100	-2.52152900	2.35994700
C	-10.03651300	-5.45143800	-0.07561000
H	-4.63943600	4.08750400	-0.01084500
H	-0.94577700	6.22507500	-0.00360000
H	0.97312600	6.21664000	0.00252600
H	4.65531600	4.05822400	0.01109200
H	5.69604700	2.17444700	0.03447900
H	6.70190200	-0.02649400	0.04135800
H	3.57442000	-5.35004800	-0.00237900
H	1.16085400	-5.52785800	-0.00514300
H	-1.21830600	-5.51880600	0.00645500
H	-3.63018400	-5.32196600	0.00345000
H	-6.71544400	0.02706600	-0.04132800
H	-5.69223700	2.21949200	-0.03452800
H	3.43149000	8.75099800	0.00785200
H	2.17015100	7.89507000	-0.88247800
H	2.16637400	7.89522000	0.89294600
H	5.18679600	7.57474100	1.28571200
H	5.29546500	5.81099800	1.31450400
H	3.98806300	6.62422400	2.18243800
H	3.99637700	6.62274600	-2.16404600
H	5.19142300	7.57426100	-1.26344500
H	5.30071000	5.81052700	-1.29052200
H	-5.60472100	7.49860000	-0.01301700
H	-5.48832700	5.97656800	-0.89974100
H	-5.49135100	5.97716200	0.87513800
H	-3.71010900	8.43885400	1.26348200
H	-2.12702300	7.65356300	1.29755700
H	-3.48606000	6.92789500	2.16394900
H	-3.47887600	6.92677200	-2.18225100
H	-3.70527100	8.43827000	-1.28327400
H	-2.12238300	7.65229900	-1.31175300
H	8.12353900	-5.31190600	2.06582300
H	8.93226100	-4.01513100	-1.93598400
H	7.42073500	-2.76929000	-3.15841500
H	5.70949200	-2.88242900	-2.71552100
H	6.65388700	-1.49467900	-2.20195900
H	5.46676300	-3.08049100	2.72719700
H	4.82592200	-4.65060400	2.27236300
H	6.29131800	-4.55680700	3.25561800
H	10.37409200	-5.64285000	1.08400700
H	9.82235200	-6.55773600	-0.31975300
H	10.73368700	-5.06561300	-0.55155000
H	-8.98227500	-3.93572600	1.93366500
H	-8.18378600	-5.24006400	-2.06776100

H -6.34219100 -4.50493100 -3.25548600
H -5.50408200 -3.03643800 -2.72660500
H -4.87851000 -4.61250700 -2.27107200
H -6.67915600 -1.43961600 2.20245200
H -7.45819400 -2.70741100 3.15811700
H -5.74788000 -2.83627300 2.71582700
H -10.79287300 -4.96696300 0.54933800
H -10.44068900 -5.54310400 -1.08818400
H -9.89870100 -6.46866800 0.31237700

*p*PHBC **16**

C -1.20592100 -0.70816600 -0.01940600
C -1.20010300 0.71887700 0.00783700
C 0.00584300 1.43818100 0.02092200
C 1.20593000 0.70951900 0.00599900
C 1.20010300 -0.71752600 -0.02123000
C -0.00583800 -1.43684200 -0.03374500
C -0.01098000 -2.88386600 -0.05942400
C 2.42317500 -1.45424400 -0.03189100
C 2.43574400 1.43567200 0.01745700
C 0.01098300 2.88515600 0.04928900
C -2.42318300 1.45555800 0.02050200
C -2.43572400 -1.43435500 -0.02888000
C -1.22881400 3.60957500 0.06431000
C -1.18141400 5.01350800 0.09243100
C 0.02034700 5.72701100 0.10568400
C 1.21964500 4.99959500 0.08966100
C 1.25972600 3.60044900 0.06248400
C 2.52964700 2.84982400 0.04779000
C 3.85305600 3.37119100 0.06529900
C 5.01804100 2.57392400 0.04952600
C 4.91573300 1.16419100 0.00659800
C 3.59484800 0.68760400 0.00104600
C 3.58887500 -0.71632600 -0.01478500
C 4.90548900 -1.20453300 -0.01437200
C 4.99522600 -2.61578300 -0.05377800
C 3.82391100 -3.40260700 -0.07146000
C 2.50421000 -2.86951600 -0.05861800
C 1.22881800 -3.60832200 -0.07261300
C 1.18141800 -5.01230000 -0.09839700
C -0.02034200 -5.72581800 -0.11075100
C -1.21964000 -4.99838000 -0.09576300
C -1.25972400 -3.59919400 -0.07084500
C -2.52964300 -2.84857100 -0.05596400
C -3.85307300 -3.37003600 -0.06884500
C -5.01804900 -2.57280000 -0.05094600
C -4.91566000 -1.16298900 -0.01103000
C -3.59480700 -0.68630100 -0.01112700
C -3.58889700 0.71762800 0.00474600
C -4.90555600 1.20573100 0.00998100
C -4.99521900 2.61690500 0.05231000
C -3.82389200 3.40375900 0.06787600
C -2.50420900 2.87076900 0.05047500
C -5.78451200 0.02550500 0.00100100
C 0.06929900 7.26761400 0.13561400
C -1.33450700 7.90162300 0.15760900
C 0.82522200 7.73402200 1.40257700
C 0.80804700 7.78302400 -1.12244900
C -0.06929100 -7.26646400 -0.13846300
C 1.33452000 -7.90051700 -0.15886900
C -0.80864500 -7.78009500 1.11997100
C -0.82459800 -7.73466300 -1.40513100
C 5.78452700 -0.02441700 -0.00207000
C -7.26181400 0.03094100 0.00435700
C -7.97563900 0.51459200 -1.11810900
C -9.37285400 0.50901500 -1.09119500
C -10.09206100 0.04343500 0.01263800
C -9.37053600 -0.42352500 1.11440000
C -7.97334600 -0.44377100 1.13215100
C -7.25336900 -0.92853800 2.37097500
C -7.25878800 1.00068700 -2.35822600
C -11.60200900 0.02036000 0.00459500
C 7.26180500 -0.03121700 0.00313100
C 7.98101400 0.44702100 -1.11825800
C 9.37807200 0.43070600 -1.08931000
C 10.09205500 -0.04045700 0.01558000
C 9.36534800 -0.50211900 1.11612700
C 7.96799100 -0.51160000 1.13186200
C 7.24255200 -0.99072600 2.36970300
C 7.26969800 0.93879100 -2.35931800
C 11.60180400 -0.07478400 0.00975600
H -2.11772700 5.55602300 0.10366800
H 2.15897400 5.54145400 0.09815900
H 3.99700300 4.44680700 0.09606800
H 5.98420500 3.07078300 0.07575100
H 5.95714000 -3.12108200 -0.07490200
H 3.95855400 -4.47947200 -0.09867700
H 2.11773100 -5.55483900 -0.10853400
H -2.15897100 -5.54025300 -0.10329700
H -3.99703500 -4.44573100 -0.09666500
H -5.98428200 -3.06975400 -0.07252100
H -5.95706700 3.12210700 0.07803500
H -3.95852300 4.48054900 0.09796600
H -1.24318800 8.99214700 0.17944400
H -1.91644900 7.63855200 -0.73179700
H -1.90479100 7.60148700 1.04280000
H 0.87384400 8.82814800 1.43550900
H 1.85124000 7.35573600 1.42959600
H 0.31895100 7.38896400 2.30984900
H 0.28857000 7.47433700 -2.03535800
H 0.85764000 8.87760400 -1.11310100
H 1.83294800 7.40499500 -1.17856500
H 1.24319300 -8.99106800 -0.17926200
H 1.90527500 -7.60160900 -1.04417300
H 1.91599700 -7.63622700 0.73048000
H -0.85820300 -8.87468700 1.11216400
H -1.83359100 -7.40203100 1.17504700
H -0.28963500 -7.47008600 2.03269600
H -0.31784000 -7.39094900 -2.31264100
H -0.87328000 -8.82883400 -1.43651500
H -1.85056800 -7.35632900 -1.43321100
H -9.91390300 0.87378500 -1.96171300
H -9.90960000 -0.77494400 1.99158900
H -7.96145100 -1.10392400 3.18526100
H -6.71142500 -1.86321400 2.19275700
H -6.51330800 -0.19878200 2.71534800
H -6.52566200 0.26734000 -2.70972500
H -7.96985100 1.18451800 -3.16804300

H	-6.70941500	1.93075700	-2.17872300
H	-12.01192000	0.82190200	-0.61723700
H	-11.98204700	-0.92788500	-0.39626000
H	-12.00953300	0.13146000	1.01393300
H	9.92318500	0.79139800	-1.95900100
H	9.90042800	-0.85785400	1.99400900
H	7.94849800	-1.17471500	3.18394200
H	6.51013600	-0.25368700	2.71497900
H	6.69080300	-1.91931800	2.18993700
H	6.53026900	0.21190300	-2.71100400
H	6.72861400	1.87391600	-2.18085500
H	7.98310200	1.11572300	-3.16861200
H	12.01870000	0.72841300	-0.60530500
H	12.00843500	0.02559100	1.02055000
H	11.97543000	-1.02269100	-0.39786300

*p*PHBC dianion **15**

C	-1.21155800	-0.70785500	-0.02152100
C	-1.20567500	0.71951400	0.01370100
C	0.00596700	1.44000600	0.02896200
C	1.21158100	0.70996500	0.01095600
C	1.20567700	-0.71740500	-0.02424200
C	-0.00595400	-1.43791000	-0.03905000
C	-0.01129600	-2.88077100	-0.07253500
C	2.43151200	-1.45409100	-0.03744900
C	2.44402500	1.43638500	0.02381100
C	0.01131100	2.88281700	0.06456200
C	-2.43153000	1.45616600	0.02845000
C	-2.44398000	-1.43430800	-0.03289500
C	-1.23612600	3.60570700	0.08285000
C	-1.18599600	5.01290200	0.11702300
C	0.02025600	5.71921500	0.13285700
C	1.22380000	4.99952600	0.11336200
C	1.26731200	3.59662200	0.08021900
C	2.52929500	2.84605500	0.06244300
C	3.86266400	3.36354100	0.08797300
C	5.02489800	2.57246100	0.06757900
C	4.94634800	1.15047300	0.00575400
C	3.60708600	0.68071200	0.00231700
C	3.60108500	-0.70844600	-0.01615500
C	4.93598300	-1.18983700	-0.01470100
C	5.00222300	-2.61270000	-0.07333300
C	3.83356600	-3.39391600	-0.09485200
C	2.50416500	-2.86493000	-0.07293800
C	1.23614300	-3.60369900	-0.08930200
C	1.18601100	-5.01093800	-0.12162300
C	-0.02024000	-5.71726700	-0.13674300
C	-1.22378400	-4.99755400	-0.11812300
C	-1.26729700	-3.59461000	-0.08678900
C	-2.52928000	-2.84404300	-0.06896900
C	-3.86267600	-3.36165700	-0.09073600
C	-5.02489200	-2.57058500	-0.06873900
C	-4.94618000	-1.14852800	-0.00940100
C	-3.60701500	-0.67863100	-0.01046400
C	-3.60113100	0.71052700	0.00806300
C	-4.93612600	1.19178400	0.01116100
C	-5.00219500	2.61457300	0.07243100
C	-3.83351900	3.39579400	0.09236000
C	-2.50414800	2.86693800	0.06658500
C	-5.80746900	0.02534500	0.00171300
C	0.06979400	7.26177400	0.16884100
C	-1.33114600	7.90255800	0.19450600
C	0.82600100	7.72878000	1.43523700
C	0.80716100	7.78780700	-1.08544900
C	-0.06977300	-7.25986700	-0.17092400
C	1.33117000	-7.90068600	-0.19556400
C	-0.80738500	-7.78445300	1.08382800
C	-0.82572500	-7.72834000	-1.43692900
C	5.80747600	-0.02353300	-0.00330800
C	-7.28117400	0.03047000	0.00340200
C	-8.01703300	0.67991100	-1.02641400
C	-9.41545800	0.68002600	-0.99949800
C	-10.14133800	0.04240900	0.00966300
C	-9.41630900	-0.59698800	1.01889300
C	-8.01827700	-0.61011800	1.03865200
C	-7.30894400	-1.25446500	2.20690500
C	-7.30778900	1.32754400	-2.19294300
C	-11.65275500	0.01725600	-0.00536400
C	7.28116900	-0.03102300	0.00144600
C	8.02484000	0.61194300	-1.02668700
C	9.42321000	0.60025500	-0.99652200
C	10.14130800	-0.04317500	0.01444600
C	9.40853300	-0.67623600	1.02214900
C	8.01045700	-0.67761200	1.03862200
C	7.29298500	-1.31568400	2.20531500
C	7.32377400	1.26538400	-2.19494900
C	11.65249300	-0.08094000	0.00326500
H	-2.12453300	5.55293300	0.12970800
H	2.16489700	5.54073300	0.12247100
H	4.00552800	4.44139000	0.13309000
H	5.98688900	3.08099300	0.11628300
H	5.96001600	-3.12957200	-0.11744000
H	3.96762700	-4.47297100	-0.13653900
H	2.12454600	-5.55099400	-0.13344900
H	-2.16488100	-5.53877300	-0.12650300
H	-4.00557100	-4.43960400	-0.13332800
H	-5.98705000	-3.07920400	-0.11341200
H	-5.95981700	3.13135900	0.12068100
H	-3.96755000	4.47474900	0.13666500
H	-1.23950300	8.99516900	0.22092500
H	-1.91406000	7.63752300	-0.69338700
H	-1.90127000	7.59302700	1.07627500
H	0.88302700	8.82488500	1.47404800
H	1.84649400	7.33700500	1.46258300
H	0.31522500	7.38102000	2.33935400
H	0.28220000	7.48321400	-1.99699100
H	0.86473800	8.88455800	-1.07358200
H	1.82658600	7.39714700	-1.14655500
H	1.23952100	-8.99332500	-0.22073400
H	1.90148200	-7.59218800	-1.07757600
H	1.91389900	-7.63461600	0.69214000
H	-0.86494200	-8.88121700	1.07322900
H	-1.82683200	-7.39374700	1.14428000
H	-0.28261900	-7.47878700	1.99512200
H	-0.31473700	-7.38167000	-2.34134700
H	-0.88279300	-8.82448800	-1.47446800
H	-1.84619100	-7.33654500	-1.46494900
H	-9.95408500	1.17749100	-1.80622900

H	-9.95589900	-1.08247200	1.83241300
H	-8.01285700	-1.47085200	3.01861900
H	-6.81154500	-2.19043100	1.93094100
H	-6.51895100	-0.59746800	2.58454100
H	-6.52222100	0.66845000	-2.57609600
H	-8.01281000	1.55144000	-3.00165100
H	-6.80485200	2.25958100	-1.91382700
H	-12.06444500	0.89846000	-0.51049400
H	-12.04679500	-0.86533500	-0.52991600
H	-12.06388400	-0.00749300	1.01061700
H	9.96789200	1.09301900	-1.80207300
H	9.94209600	-1.16597200	1.83709600
H	7.99334300	-1.53893700	3.01823700
H	6.50846400	-0.65149100	2.58178800
H	6.78734500	-2.24677900	1.92797500
H	6.53282900	0.61333600	-2.57907600
H	6.82902700	2.20231000	-1.91744500
H	8.03231600	1.48220100	-3.00251500
H	12.07247300	0.79207800	-0.50913800
H	12.06118600	-0.09948700	1.02039400
H	12.04041600	-0.97170800	-0.51192400

Mulliken charges and spin densities distribution of triplet biradical form of *m*PHBC **8**

1	C	-0.013793	-0.001861
2	C	0.075038	0.004583
3	C	-0.020927	-0.003869
4	C	-0.024327	-0.000823
5	C	0.074062	0.002896
6	C	-0.013655	0.000520
7	C	0.134955	-0.018928
8	C	0.047532	-0.060937
9	C	0.042657	0.032663
10	C	0.040561	0.037329
11	C	0.046263	-0.061475
12	C	0.135299	-0.017626
13	C	0.052510	-0.048454
14	C	-0.148650	0.047646
15	C	0.166928	-0.025338
16	C	-0.138247	0.050254
17	C	0.062072	-0.018824
18	C	0.059638	-0.016635
19	C	-0.151090	0.045965
20	C	0.167943	-0.024156
21	C	-0.138726	0.049971
22	C	0.054494	-0.047037
23	C	0.087018	0.167461
24	C	-0.069598	-0.078489
25	C	-0.088837	0.147923
26	C	0.155206	-0.135676
27	C	-0.121153	0.122546
28	C	-0.164605	0.067812
29	C	0.153770	-0.088526
30	C	-0.089376	0.128304
31	C	-0.073314	-0.018535
32	C	0.070991	0.103351
33	C	0.070834	0.101376
34	C	-0.073392	-0.016751
35	C	-0.089706	0.126837
36	C	0.153812	-0.086512
37	C	-0.164381	0.065753
38	C	-0.121431	0.124782
39	C	0.155126	-0.136259
40	C	-0.088482	0.146964
41	C	-0.068545	-0.078946
42	C	0.088461	0.166646
43	C	-0.169810	**0.602461**
44	C	-0.169627	**0.603634**
45	C	-0.036669	0.001245
46	C	-0.004355	0.000040
47	C	0.009599	-0.000319
48	C	0.009463	-0.000319
49	C	-0.035723	0.001312
50	C	-0.002909	0.000020
51	C	0.008413	-0.000374
52	C	0.008484	-0.000374
53	C	-0.033255	-0.067683
54	C	0.132598	0.040579
55	C	-0.116515	-0.013765
56	C	0.153401	0.029530
57	C	-0.116456	-0.013853
58	C	0.132372	0.040430
59	C	-0.023528	0.001410
60	C	-0.023510	0.001400
61	C	-0.029962	0.000304
62	C	-0.033171	-0.067561
63	C	0.132306	0.040390
64	C	-0.116483	-0.013841
65	C	0.153402	0.029520
66	C	-0.116535	-0.013766
67	C	0.132639	0.040547
68	C	-0.023553	0.001397
69	C	-0.023539	0.001409
70	C	-0.030012	0.000304

Mulliken charges and spin densities distribution of triplet biradical form of *p*PHBC **16**

1	C	0.074493	0.007092
2	C	0.076168	0.006010
3	C	-0.102883	-0.011173
4	C	0.074551	0.007088
5	C	0.076108	0.006015
6	C	-0.102888	-0.011177
7	C	0.110543	0.074574
8	C	0.046922	-0.053418
9	C	0.048511	-0.053445
10	C	0.110524	0.074574
11	C	0.046877	-0.053416
12	C	0.048557	-0.053446
13	C	0.026573	-0.065667
14	C	-0.142014	0.087142
15	C	0.164339	-0.046728
16	C	-0.132484	0.093488
17	C	0.029182	-0.066166
18	C	0.102035	0.163055

19	C	-0.074123	-0.075499
20	C	-0.091004	0.148238
21	C	0.156193	-0.133017
22	C	-0.135366	0.100810
23	C	-0.135327	0.102654
24	C	0.155971	-0.133766
25	C	-0.090816	0.147255
26	C	-0.073059	-0.075823
27	C	0.103476	0.161870
28	C	0.026577	-0.065667
29	C	-0.142044	0.087140
30	C	0.164339	-0.046727
31	C	-0.132516	0.093485
32	C	0.029186	-0.066167
33	C	0.102026	0.163050
34	C	-0.074128	-0.075488
35	C	-0.091129	0.148220
36	C	0.156098	-0.133008
37	C	-0.135250	0.100796
38	C	-0.135448	0.102668
39	C	0.156088	-0.133777
40	C	-0.090690	0.147277
41	C	-0.073056	-0.075836
42	C	0.103489	0.161881
43	C	-0.172574	**0.604408**
44	C	-0.035483	0.002400
45	C	-0.003206	0.000038
46	C	0.009365	-0.000683
47	C	0.009381	-0.000696
48	C	-0.035474	0.002400
49	C	-0.003223	0.000038
50	C	0.009358	-0.000696
51	C	0.009362	-0.000684
52	C	-0.172548	**0.604411**
53	C	-0.032658	-0.067280
54	C	0.132338	0.039804
55	C	-0.116516	-0.013453
56	C	0.153184	0.028590
57	C	-0.116510	-0.013413
58	C	0.132568	0.039707
59	C	-0.023558	0.001384
60	C	-0.023527	0.001417
61	C	-0.030097	0.000289
62	C	-0.032665	-0.067277
63	C	0.132261	0.039790
64	C	-0.116475	-0.013449
65	C	0.153177	0.028593
66	C	-0.116545	-0.013421
67	C	0.132648	0.039728
68	C	-0.023535	0.001368
69	C	-0.023555	0.001430
70	C	-0.030094	0.000289
